# Highly luminescent scintillating hetero-ligand MOF nanocrystals with engineered Stokes shift for photonic applications

**DOI:** 10.1038/s41467-022-31163-0

**Published:** 2022-06-17

**Authors:** J. Perego, Charl X. Bezuidenhout, I. Villa, F. Cova, R. Crapanzano, I. Frank, F. Pagano, N. Kratochwill, E. Auffray, S. Bracco, A. Vedda, C. Dujardin, P. E. Sozzani, F. Meinardi, A. Comotti, A. Monguzzi

**Affiliations:** 1 Dipartimento di Scienza dei Materiali, Università degli Studi Milano-Bicocca, via R. Cozzi 55, 20125 Milano, Italy; 2 FZU Institute of Physics, Academy of Sciences of the Czech Republic, Prague, Czech Republic; 3 CERN, Geneva, Switzerland; 4 Ludwig Maximilian University of Munich, Geschwister-Scholl-Platz 1, Munich, Germany; 5 Dipartimento di Fisica “Giuseppe Occhialini”, Università degli Studi Milano-Bicocca, Piazza della Scienza 3, 20126 Milano, Italy; 6 University of Vienna, Vienna, Austria; 7 Institut Lumière Matière, UMR5306 Université Lyon 1-CNRS, Université de Lyon, 69622 Villeurbanne cedex, France

**Keywords:** Organic-inorganic nanostructures, Molecular self-assembly, Optical materials and structures

## Abstract

Large Stokes shift fast emitters show a negligible reabsorption of their luminescence, a feature highly desirable for several applications such as fluorescence imaging, solar-light managing, and fabricating sensitive scintillating detectors for medical imaging and high-rate high-energy physics experiments. Here we obtain high efficiency luminescence with significant Stokes shift by exploiting fluorescent conjugated acene building blocks arranged in nanocrystals. Two ligands of equal molecular length and connectivity, yet complementary electronic properties, are co-assembled by zirconium oxy-hydroxy clusters, generating crystalline hetero-ligand metal-organic framework (MOF) nanocrystals. The diffusion of singlet excitons within the MOF and the matching of ligands absorption and emission properties enables an ultrafast activation of the low energy emission in the 100 ps time scale. The hybrid nanocrystals show a fluorescence quantum efficiency of ~60% and a Stokes shift as large as 750 meV (~6000 cm^−1^), which suppresses the emission reabsorption also in bulk devices. The fabricated prototypal nanocomposite fast scintillator shows benchmark performances which compete with those of some inorganic and organic commercial systems.

## Abstract (web summary)

The development of highly luminescent materials such as large Stokes shift fast emitters is desirable for their potential application in photonics. Here the authors engineer hetero-ligand metal-organic frameworks nanoparticles to achieve high emission yield, large Stokes shift and realize a prototypal fast scintillator.

## Introduction

**Fig. 1:**
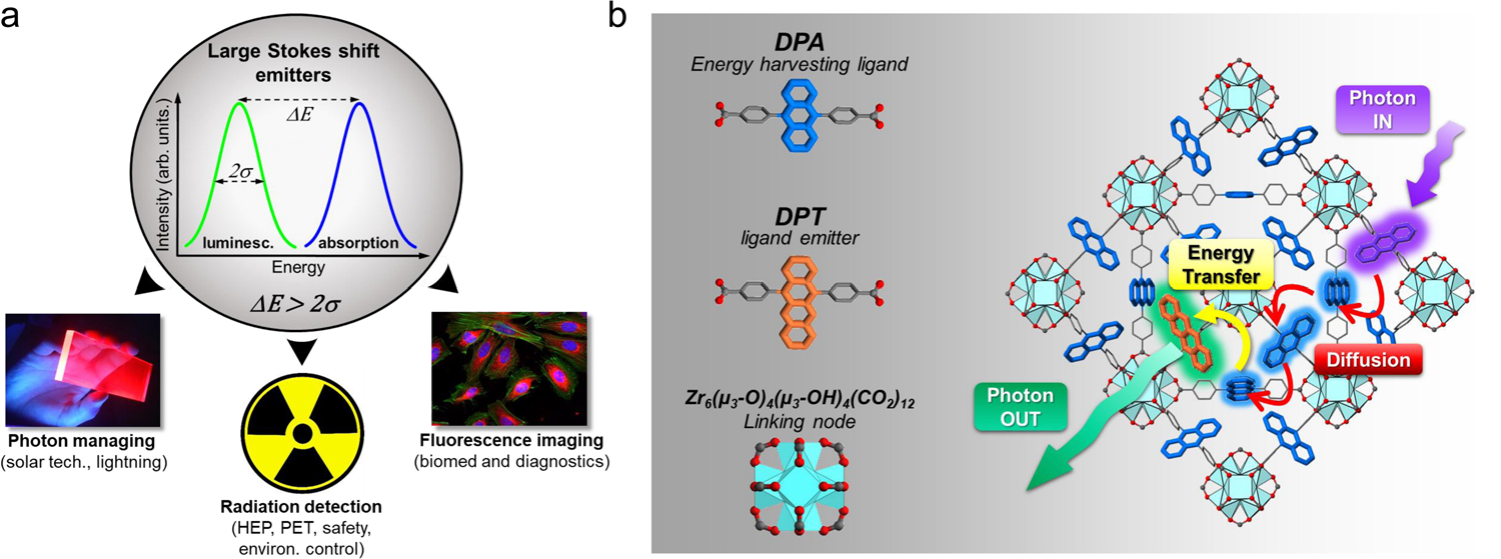
Large Stokes shift emitters concept, applications and realization with engineered *hetero*-ligand MOF nanocrystals based on fluorescent ligands. **a** Definition of large Stokes shift emitter. The energy difference between absorption and luminescence maxima *ΔΕ* is larger than luminescence half-height bandwidth *2σ*, thus avoiding reabsorption. **b** (left) Molecular structure of the MOF nanocrystals building blocks, namely the 9,10-diphenyl-antracenedicarboxylate fluorescent ligand (DPA), the 5,12-diphenyl-tetracenedicarboxylate fluorescent co-ligand (DPT) and the linking node zirconium oxo-hydroxy cluster. (right) Sketch of the energy flux after generation of a DPA singlet exciton, in this case upon absorption of a UV photon of energy *hν*_*in*_. The singlet exciton diffuses within the crystalline framework until it reaches the co-ligand DPT. The latter is excited by non-radiative energy transfer and then recombines radiatively by emitting a green photon of energy *hν*_*out*_ with a Stokes shift *ΔΕ* = *hv*_*in*_ − *hv*_*out*_.

The Stokes shift is an important property of luminescent materials, defined as the energy difference (*ΔE*) between the absorption band maximum and the emission spectrum maximum^1^. The value of *ΔE* is a key parameter in photonic devices and applications because, at a first approximation, it enables to directly estimate if an emitter would be affected by significant reabsorption of the generated light. For example, if the *ΔE* value is lower or similar to the bandwidth of the absorption and emission spectra, the consequent intrinsic extensive inner-filter effect can heavily limit the lighting performance of bulk photonic devices, and, in the worst cases, it can also affect the kinetics of the luminescence generation^2–4^. Conversely, if *ΔE* is fairly larger than the spectral bandwidths, in other words the overlap between the absorption and emission bands is minimized or completely negligible, the system can be considered a large Stokes shift emitter that works as wavelength shifter with no inner filter effects (Fig. 1a). These reabsorption-free materials are highly desirable for several applications. For example, in fluorescence imaging large Stokes shift optical probes allow obtaining high contrast images with limited excitation stray light, avoiding the use of expensive filtering components or time-consuming image post-processing^5, 6^. For solar applications, large Stokes shift emitters are undoubtedly the most promising materials to realize luminescent solar concentrators without reabsorption of the condensed radiation^7^. Similarly, the sensitivity of scintillating detectors for ionizing radiation would greatly benefit from the use of fast emitters with no reabsorption^8^ showing good light output intensity without effects on the scintillation pulse timing, as required by the most advanced medical imaging techniques such as time-of-flight positron emission tomography (ToF-PET)^9^ and high-rate high-energy physics (HEP) experiments. The extensive recent literature in the field of semiconductor nanocrystals testifies this very actual interest in large Stokes shift emitters. In these materials, for example, the *ΔE* can be tuned by doping semiconductors with electronic impurities^10^, resulting in the appearance of intragap states from which red shifted luminescence is produced. A notable *ΔE* value as large as 1 eV can be achieved^11^, but a current unsolved drawback is the slow luminescence kinetics that limit their use for fast timing applications in nanosecond time scale and below^12–14^. Moreover, in photonic devices where fast timing is foreseen, traditional wavelength shifters exploiting radiative energy transfer cannot be employed, because of the consequent slowing down of emitted light pulse.

In the search for fast emitters with remarkably large Stokes shift, we selected Metal-Organic Frameworks (MOFs), which constitute a solid platform to build materials wherein active struts perform tailored functions. Synthetic procedures based on self-assembly processes enable the controlled framing of struts in the porous crystalline architecture and the regulation of distances among linkers^15, 16^. The impressive versatility of MOFs promoted several applications such as gas storage^17, 18^, catalysis and dynamic materials^19–23^, and triggered the most recent advances in the field of luminescent MOFs^24–29^. This hot topic gave birth to a new class of optically active nanomaterials with tailorable electronic properties for photonics and optoelectronics, sensing, and biomedicine^26, 30–32^. MOFs are also excellent candidates to be used in light-emitting devices, due to their structural diversity and tunable emission. A key advantage is the possibility to design their framework composition and structure which control both optical and energy-transport properties^33^, such as those required for managing site- specific photoreactions^34^, or multi-excitonic processes^35^. Therefore, optimized luminescent MOF nanocrystals can represent the next generation of luminescent materials with a potential impact comparable to their inorganic counterpart colloidal semiconductor nanocrystals.

Among conjugated molecules, polycyclic aromatic hydrocarbons of the acene family have attracted great interest for various applications in photon managing such as photon upconversion and singlet fission because of their peculiar electronic properties^36–39^. Here we present the fabrication of wavelength shifting MOF nanocrystals with fine-tuned composition, wherein tetracene-bearing fluorescent moieties were co-assembled with anthracene-based linkers to engineer the system emission properties and obtain significant energy down conversion of the emitted photons with respect to the absorption, thus maximizing the emission Stokes shift. The strategy of increasing the number of fused aromatic rings in the ligand core, yet maintaining a constant spacing between the coordinating groups, is successful in providing a series of customized hetero-ligand Zr-MOFs, with excellent fluorescence efficiency and negligible reabsorption. MOF nanocrystals are obtained by co-assembling the green-fluorescent chromophore 5,12-diphenyl-tetracenedicarboxylate (DPT) and the blue-emitting ligand 9,10-diphenyl-anthracenedicarboxylate (DPA) with Zr oxy-hydroxy nodes (Fig. 1b). By exploiting the diffusion within the crystalline framework of singlet molecular excitons generated on DPA ligands, the incorporated DPT co-ligands are excited by means of non-radiative energy transfer and subsequently recombine radiatively producing photons with a *ΔE* as large as 750 meV (~6000 cm^−1^). Matching the emission frequency of the anthracene with the absorption of the tetracene units enables an efficient energy transfer (ET) of 97% and photoluminescence quantum yield (QY) of ~60% even with a low DPT loading of 8% with respect to DPA (denoted Zr-DPT:DPA-8%). Such a low loading enables to preserve the structural features of the parent homo-ligand nanocrystals. We demonstrate the potential technological transfer of the hetero-ligand fluorescent nanomaterials by realizing a prototypal fast polymeric nanocomposite scintillator that shows enhanced performances with respect to the homo-ligand nanocrystals, thus achieving benchmark values competing with those of several organic and inorganic commercial systems.

## Results

### Synthesis and structural properties of hetero-ligand MOFs

**Fig. 2:**
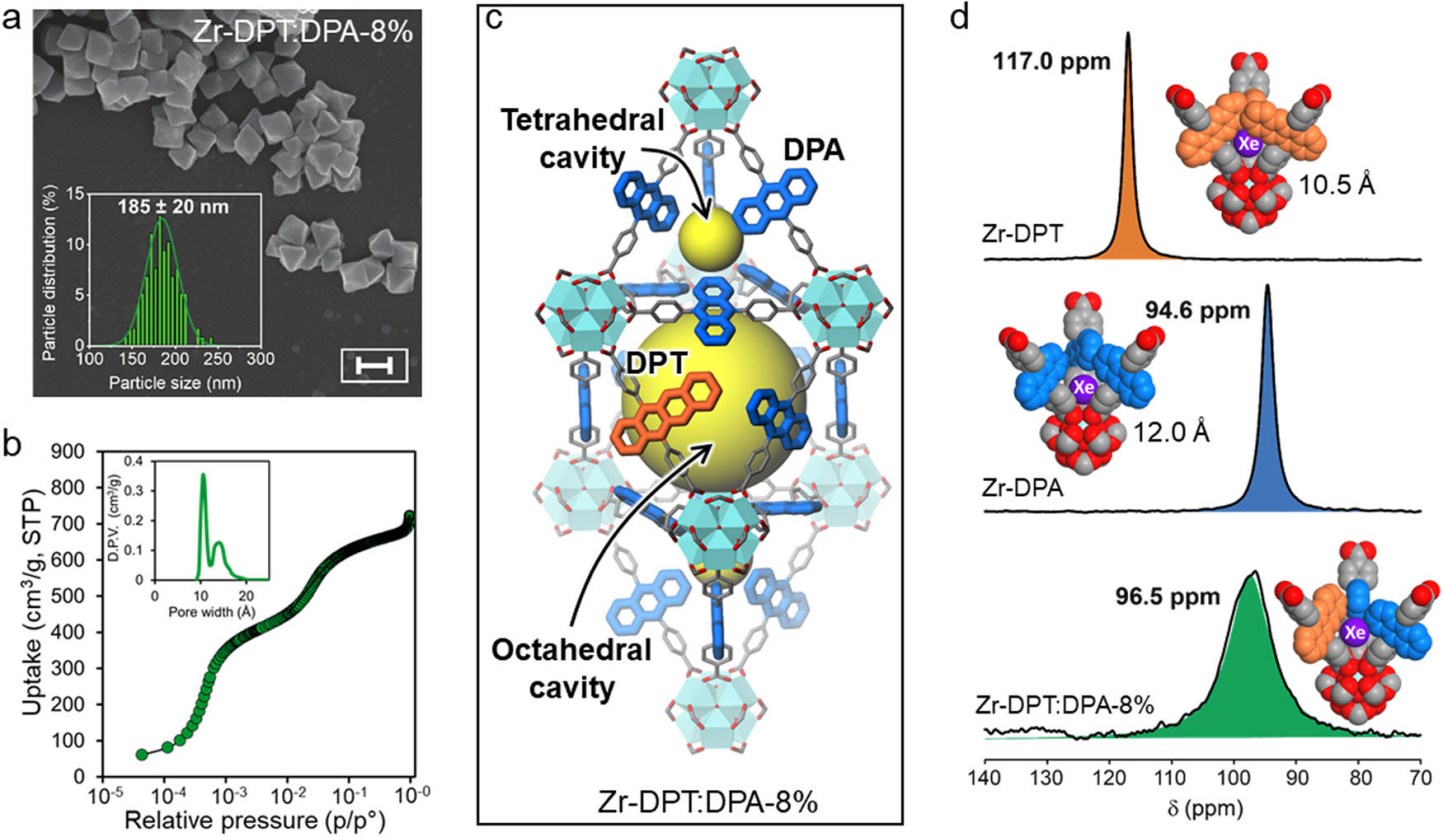
Structural properties of *hetero*-ligand zirconium-based metal-organic framework (MOF) nanocrystals. **a** Scanning electron microscopy image of MOF nanocrystals realized with a content of molar DPT fraction of 8% (Zr-DPT:DPA-8%). The inset reports the distribution of MOF size (Scale bar: 200 nm). **b** N_2_ adsorption isotherm collected at 77 K. The inset showed the pore size distribution with two distinct cavities of 10.8 Å and 14.5 Å. **c** Crystal structure of Zr-DPT:DPA highlighting the tetrahedral and octahedral cavities (yellow spheres). **d** Hyperpolarized laser-assisted ^129^Xe NMR spectra of *hetero*-ligand nanocrystals compared with *homo*-ligand MOFs based on DPA and DPT. The spectra are fitted with a Lorentzian line-shape function (colored areas) for quantitative analysis.

We designed and prepared the new conjugated tetracene-containing ligand (DPT) to be co-assembled with the anthracene-based linker (DPA) by a solvothermal process (Methods and Supplementary Information): the two molecules DPA (QY = 0.96) and DPT (QY = 0.80) were chosen because of their complementary absorption/emission properties that make them an ideal donor (DPA) and acceptor (DPT) pair for non-radiative energy transfer (Supplementary Information). The two rod-like ligands with identical end-to-end length and connectivity were co-assembled by zirconium oxy-hydroxy clusters, generating a series of isostructural hetero-ligand MOF nanocrystals with modulated composition ranging from 0.1 to 8% of DPT molar fraction (Zr-DPT:DPA-x%, Fig. 1b). For comparison, the homo-ligand MOFs were synthesized using separately the single ligands (Zr-DPA and Zr-DPT, respectively). The composition of hetero-ligand Zr-MOFs is in agreement with the feeding ratio, as shown by ^1^H NMR of digested samples. Connectivity, purity, and thermal stability were demonstrated by FT-IR, ^13^C MAS NMR and TGA analysis (Supplementary Figs. 1–40). Scanning electron microscopy (SEM) images of the Zr-DPT:DPA specimens reveal a homogenous population of nanocrystals with octahedral morphology. Figure 2a depicts the SEM image of the Zr-DPT:DPA-8% sample, which consists of a nanocrystal ensemble with average size of 185 ± 20 nm.

Upon activation at 130 °C under vacuum, the hetero-ligand Zr-MOF nanocrystals exhibit high crystallinity and a cubic crystal structure (Fm-3m) with **fcu** topology, as established by PXRD Rietveld refinement, which corresponds to that of parent Zr-DPA and Zr-DPT MOFs (Supplementary Figs. 13–17). Twelve ligands coordinate to each Zr-based node (Zr_6_(μ_3_-O)_4_(μ_3_-OH)_4_(CO_2_)_12_ cluster) and yield a framework containing interconnected octahedral and tetrahedral cavities (Fig. 2c). Thus, the ligands are arranged at a sufficiently short center-to-center distance of 11.7 Å that enables both fast exciton diffusion and non-radiative energy transfer (Supplementary Information). Consistently with the crystal structure, N_2_ adsorption isotherms at 77 K (Fig. 2b and Supplementary Figs. 18, 37–40) showed remarkable surface areas up to 3000 m^2^/g: step-wise profiles were evidenced at low partial pressures in a logarithmic plot, caused by the sequential filling of the well-differentiated tetrahedral and octahedral cavities (pore size of 10.8 Å and 14.5 Å, respectively, in the Zr-DPT:DPA-8%).

Exploration of the pores by xenon atoms entering the framework was directly reported by hyperpolarized ^129^Xe NMR in natural isotopic abundance. Such laser-assisted technique exploits the excitation transfer from optically pumped Rb vapors to ^129^Xe nuclei. Excited xenon fluxes from the fringe magnetic field to the porous substance at the center of the magnet and collects information on the confining sites of the material with a large downfield shift from the free-gas resonance^40^. Pore symmetry and size can be measured with extraordinary sensitivity. The homogeneity of the samples can be proved by xenon running fast along the galleries within the NMR timescale if a single signal is shown. In fact, the homo-ligand Zr-DPA and Zr-DPT MOFs show a single well-distinct sharp signal each, at δ = 94.6 and δ = 117.0 ppm, respectively (Fig. 2d): this reflects more restricted pores in Zr-DPT, caused by the flag-like tetracene aromatic moiety protruding laterally into the nano-channels, with a steric hindrance larger than that of anthracene unit. Remarkably, the hetero-ligand Zr-DPT:DPA-8% MOF exhibits a single peak and a chemical shift at δ = 96.5 ppm, which corresponds precisely to the expected weighted-average of the two chemical shifts of the homo-ligand samples. No residual peaks of the homo-ligand MOFs are present, demonstrating the excellent structural homogeneity of the co-assembled frameworks.

### Photoluminescence properties of hetero-ligand MOFs

**Fig. 3:**
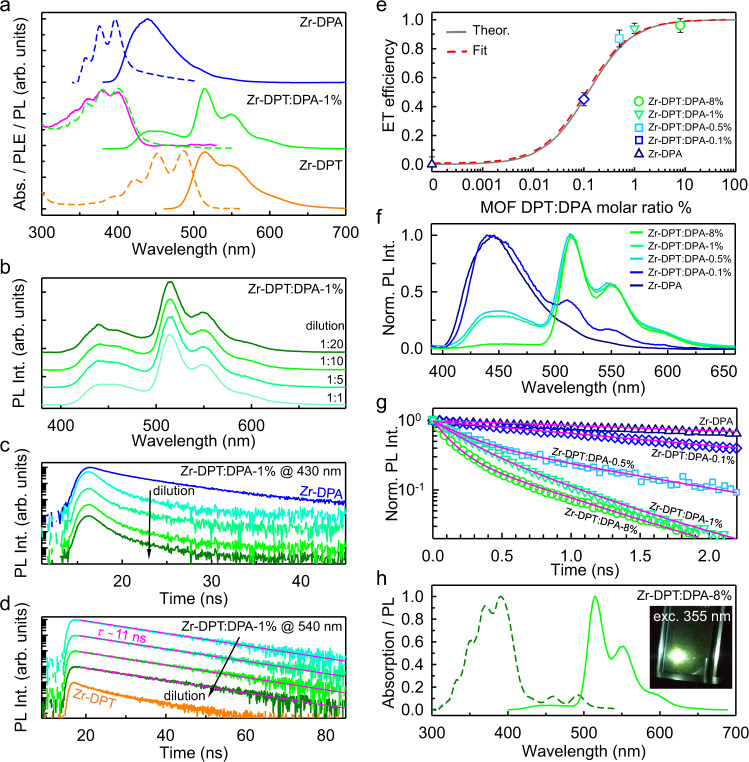
Photophysical properties of *hetero*-ligand MOF nanocrystals. **a** Normalized absorption (dashed line) and photoluminescence (PL, solid line) spectra of the *hetero*-ligand Zr-DPT:DPA-1% nanocrystals (0.1 mg mL^−1^) in tetrahydrofuran (THF) compared with *homo*-ligand MOFs based on DPA (Zr-DPA, 0.1 mg mL^−1^) and DPT (Zr-DPT, 0.1 mg mL^−1^). The excitation wavelength is 355 nm. The pink solid line is the excitation photoluminescence spectrum of the Zr-DPT:DPA-1% dispersion recorded at 540 nm. **b** PL spectrum of the Zr-DPT:DPA-1% dispersion at different dilution ratios under 355 laser excitation and **c**, **d** PL intensity decay with time recorded at 430 nm (**c**) and 540 nm (**d**) at the same dilution ratios of Zr-DPT:DPA-1% in comparison with that of Zr-DPA and Zr-DPT reference nanocrystals, respectively. **e** Energy transfer efficiency *ϕ*_*ET*_ between DPA and DPT co-ligands as a function of their relative molar ratio in the MOF framework. The theoretical *ϕ*_*ET*_ (solid line) is calculated considering a diffusion-mediated Förster energy transfer with interaction radius of 2.8 nm derived from ligands properties. The fit of experimental data (dashed line) results a characteristic interaction radius of 3.0 nm. Error bars show the standard deviation calculated for reported values. **f** Normalized PL spectra of the *hetero*-ligand nanocrystals dispersions series (0.1 mg mL^−1^) and **g** time-resolved PL spectra recorded at 430 nm. Solid lines are the fit of the data with multi-exponential decay function. **h** Absorption (dashed line) and PL (solid line) spectrum of Zr-DPT:DPA-8% nanocrystals in THF (0.1 mg mL^−1^, optical path 1 cm). The excitation wavelength is 355 nm. The inset is a digital picture of the dispersion in the quartz cuvette under 355 nm excitation.

The photophysical properties of the obtained MOF nanocrystals are investigated by means of photoluminescence spectroscopy. Figure 3a shows the optical absorption and continuous wave photoluminescence spectra of the hetero-ligand Zr-DPT:DPA-1% in tetrahydrofuran dispersion (0.1 mg mL^−1^). We observe a main absorption band in the near-UV spectral matching the profile of the Zr-DPA reference sample, because the absorption of the few DPT substituents is negligible. Upon photoexcitation at 355 nm, the hetero-ligand nanocrystals show a broad luminescence in the visible spectrum. The most intense emission peaked at 515 nm and its vibronic replicas at 550 nm and 590 nm match the photoluminescence profile of the isolated DPT ligand (Supplementary Figs. 7, 43, and 45) and of the Zr-DPT MOF as a control sample MOFs. This result suggests that the green luminescence is generated by the radiative recombination of singlet excitons on DPT co-ligands populated by energy transfer from directly excited DPAs ligands, as demonstrated by the excitation photoluminescence spectrum recorded at 540 nm that follows the DPA absorption profile (Fig. 3a). The weak residual blue luminescence peaked at 430 nm mirrors an energy transfer yield (*ϕ*_*ET*_) lower than unity. Nevertheless, the presence of this residual emission is crucial investigating the antenna effect sketched in Fig. 1b that occurs in the framework. Figure 3b depicts the normalized photoluminescence spectra of the Zr-DPT:DPA-1% dispersion at incrementing dilution ratios. The relative intensity of the green and blue emission components is unchanged. This is a crucial result. The data clearly indicate that *ϕ*_*ET*_ is independent from the total DPT concentration in the dispersion, thus demonstrating that each nanocrystal works as an individual emitter whose green luminescence is activated by intra-crystal energy transfer. Conversely, if DPAs and DPTs would experience energy transfer as two separated entities, i.e., in a standard bicomponent solution, the *ϕ*_*ET*_ value should decrease following the dilution level that reduces the concentration of the energy acceptor DPT and therefore the transfer probability^1, 41^. Consequently, the relative intensity of the green *vs*. blue component should be reduced as well.

The occurrence of intra-crystal energy transfer is confirmed by time resolved experiments. Figure 3c shows the photoluminescence intensity decay with time at 430 nm as a function of the dilution level. The signal decay in the hetero-ligand MOF is faster than in the Zr-DPA reference, indicating an efficient energy transfer (vide infra)^1^, but still no change in the decay kinetics is observed at different dilutions, thus demonstrating that the DPA-DPT interaction is unaffected by dilution. Similarly, Fig. 3d shows that the green photoluminescence intensity at 540 nm decays with time as a single exponential function with a characteristic lifetime of τ ~ 11 ns (Supplementary Table 7,) regardless of the dilution level. This is another key result, indeed, the observed lifetime is almost identical to that one of the DPT molecule (11.5 ns, Supplementary Fig. 41), and is significantly longer with respect to the reference homo-ligand DPT-MOFs (7.7 ns, Supplementary Table 7). These findings suggest that co-assembled DPT ligands are effectively incorporated and framed as non-interacting single molecules^42^ within the nanocrystal architecture and preserve their excellent luminescence properties pivotal for the fabrication of photonic devices.

Once assessed the validity of the synthetic strategy, we investigate quantitatively the energy transfer mechanism. As sketched in Fig. 1b, the activation of DPT luminescence occurs by energy transfer during the random diffusion within the framework of an excited DPA singlet exciton, which is created upon light absorption or free-charge recombination in scintillation^43^. Before spontaneous recombination, the singlet moves from the original position by an average diffusion length *L*^44–46^. This implies that if a DPT moiety is placed at a distance shorter than *L* from the position where the DPA exciton is created, the energy transfer can most likely occur before singlet recombination, thus without energy dispersion. The DPA-DPT interaction is of the Förster type, which characteristic interaction radius of *R*_*fs*_ = 2.8 nm is calculated from the dyes absorption and emission properties (Supplementary Fig. 42 and Supplementary Eq. (4) adapted to the DPT-DPA pair). Considering the structure and size of MOF^47^, the theoretical energy transfer rate *k*_*ET*_ and efficiency *ϕ*_*ET*_ are therefore calculated as a function of the nanocrystal composition under the assumption of an exciton diffusion-mediated energy transfer process in the rapid diffusion limit (Supplementary Information, Section 5)^41, 48^. The solid line in Fig. 3e depicts the theoretical ϕ_ET_ vs. the DPT ligand fraction in the hetero-ligand MOFs, expressed as the nominal DPT:DPA relative molar ratio employed for the synthesis. The plot shows that an excellent *ϕ*_*ET*_ ~ 0.9 (90%) is reached with a DPT content as low as 1%. This suggests that the proposed strategy to achieve large Stokes shift is already effective with low levels of DPT substitution in the DPA based MOF. In this way the risk of affecting the MOF structural properties is minimized. The predicted *ϕ*_*ET*_ is compared with the one measured in the series of nanocrystals. Figure 3f shows the normalized photoluminescence spectra of the MOF samples dispersed in tetrahydrofuran under 355 nm excitation (Supplementary Fig. 44). The blue emission from DPA ligands is almost completely switched off in the Zr-DPT:DPA-8% sample, which works as an effective wavelength shifter. The experimental *ϕ*_*ET*_ value is calculated from the time resolved photoluminescence data shown in Fig. 3g. The intensity of the blue luminescence at 430 nm decays as multi-exponential function (Supplementary Table 7), whose average lifetime shortens progressively by increasing the DPT amount, according to the corresponding increment of *k*_*ET*_. The circles in the main panel mark the observed *ϕ*_*ET*_, which increases up to 96% in the Zr-DPT:DPA-8% sample. The fit of data with a diffusion-mediated energy transfer kinetics results in an experimental interaction radius ${R}_{{fs}}^{* }$ = 3.0 nm for the DPA/DPT pair, in agreement with the proposed model. It is worth noting that the decay of the negligible residual blue emission intensity shows a multiexponential behavior (Fig. 3g, Supplementary Table S7, Supplementary Fig. 44) that indicates the presence of different population of emitters. The fast component (67% of the total signal) has a lifetime *τ*_*fast*_ = 120 ps ≈ [*k*_*ET*_]^−1^ and accounts for the fraction of nanocrystals fully active as wavelength shifters (*ϕ*_*ET*_ = 1, Supplementary Eq. (9)). The photoluminescence quantum efficiency of fully active nanocrystals can be therefore estimated as large as QY = 0.59 ± 0.07 by correlating the photoluminescence yield measured for the ensemble in dispersion QY^ens^ = 0.56 ± 0.06 (Fig. 3h, Methods) and the time resolved photoluminescence data (Supplementary Information, Section 5). Therefore, a huge photoluminescence Stokes shift of 750 meV with highly efficient emission is achieved while preserving the fast time response that outperforms the one obtained in doped semiconductor quantum dots^49, 50^. The slower emission component is ascribed to a sub-population of nanocrystals in the ensemble containing statistically a lower amount of DPT. However, despite being incomplete, the *ϕ*_*ET*_ for this sub-population is as large as 0.9 (Supplementary Table 7 and Supplementary Eq. (9)), thus minimizing the energy losses. This means that the DPT level in this population of MOFs should be at least 2% (Fig. 3e), in agreement with the presence of a fraction of nanocrystals with dopant content at the lower limits of the distribution observed from HP Xe NMR. Nevertheless, the average doping level of 8% matches the global photoluminescence properties of the MOF ensemble. It is worth noting that both fast energy transfer and high-yield Stokes shifted fluorescence are achieved by using low DPT amounts, thanks to the accurate design of hetero-crystals made by co-ligands with identical length and connectivity but strictly complementary optical properties owing to the change of their conjugation length. In such a way, the fast singlet excitons diffusion of the homo-ligand MOFs is unaffected by the presence of the acceptor moiety in the hetero-ligand structure, enabling ultrafast activation of the low energy emitters. The results obtained, therefore, highlight once more the potential of composition-tuned MOFs to develop efficient loss-free energy harvesting and transport systems that can mimic, for example, also natural photosynthetic mechanisms where fast energy migration is required.

### Radioluminescence and scintillation of MOFs nanocomposites

**Fig. 4:**
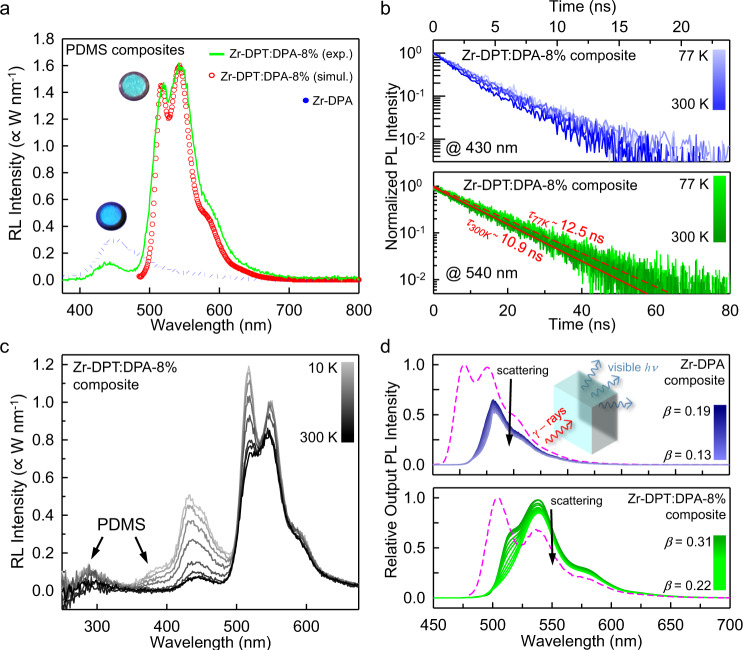
Luminescence properties of large Stokes shift MOF-based nanocomposites under X-ray exposure. **a** Experimental and simulated radioluminescence (RL) spectrum of nanocomposites loaded with *hetero*-ligand Zr-DPT:DPA-8% and *homo*-ligand Zr-DPA nanocrystals fabricated by using polydimethylsiloxane (PDMS) as host polymer matrix (0.5% wt, 1 mm thickness) under X-ray exposure (digital pictures). **b** Zr-DPT:DPA-8% nanocomposite photoluminescence (PL) intensity decay recorded at 430 nm (top) and 540 nm (bottom) as function of the temperature under pulsed laser excitation at 405 nm. **c** RL spectrum of the Zr-DPT:DPA-8% nanocomposite as a function of the temperature. **d** Simulated output emission spectrum and efficiency (*β*) as a function of the scattering level (from 0 cm^−1^ to 20 cm^−1^) in a 10 × 10 × 5 mm scintillator. Dashed lines depict the single molecule spectrum of the DPA (top) and DPT (bottom) chromophores for reference.

Given their excellent luminescence properties, we tested the hetero-ligand MOF nanocrystals as emitters in bulk scintillating devices^51^ typically employed as a detector of ionizing radiation where large Stokes shift is usually required to maximize the extraction of scintillation light^8^. Figure 4a shows the radioluminescence spectrum under soft X-ray exposure of a composite scintillator (thickness 0.1 cm, diameter 1 cm) fabricated by loading Zr-DPT:DPA-8% nanocrystals in a polydimethylsiloxane (PDMS) matrix. The relative amount of MOFs vs. PDMS is 0.5% in weight (Methods, Supplementary Figs. 46–49). The radioluminescence spectrum (solid line) is dominated by a structured green emission peaked at 530 nm with a weak residual emission at 430 nm, suggesting that the scintillation light is produced by radiative recombination of DPT singlet excitons. The absence of reabsorption is demonstrated by the possibility to clearly observe the first vibronic replica in the emission spectrum at 515 nm, as in the diluted dispersion case, despite the high concentration of embedded nanocrystals (Fig. 3d). This result is in excellent agreement with the simulated emission spectrum (circles) calculated considering the light propagation in the device (Methods). Radioluminescence measurements under continuous irradiation up to around one hundred Gy demonstrate the emission stability and the absence of long-time phosphorescence due to delayed carrier recombination (Supplementary Fig. 51). No significant variation of the radioluminescence intensity can be observed by heating the sample up to 50 °C, demonstrating a good thermal stability (Supplementary Fig. 47). After 30 days of exposure to the atmospheric moisture, a mere 10% reduction of the emission intensity is observed, demonstrating a good resistance to molecular oxygen despite the tendency of tetracene to photobleaching (Supplementary Fig. 51).

The nanocomposite shows a five-fold improved radioluminescence intensity with respect to a reference composite made with homo-ligand Zr-DPA (dotted line) MOFs. Its scintillation yield *ϕ*_*scint*_, defined as the number of emitted photons for each MeV of deposited energy for ionizing radiation, is assessed at around 5000 ± 300 ph MeV^−1^ (Supplementary Fig. 53). This value demonstrates the success of the proposed strategy to enhance the scintillation performance of composite materials based on fluorescent MOF nanocrystals, which show a *ϕ*_*scint*_ comparable to that of commercial plastic and inorganic scintillators^52^.

We further investigated the composite emission by time resolved photoluminescence and radioluminescence experiments as a function of the temperature. At room temperature the composite photoluminescence intensity at 540 nm decays with a characteristic lifetime of 10.9 ns (Fig. 4b, bottom) matching that of the single molecules (Supplementary Fig. 41). This finding is crucial to point out an important feature of the hetero-ligand emitters. In the case of homo-ligand MOFs, the use of high loading levels induces a shortening of the photoluminescence lifetime and efficiency reduction, because of a partial aggregation of poorly dispersed crystals that can limit also the surface passivation effect of the host matrix^43^. Conversely, in hetero-ligand MOFs this effect appears absent, suggesting that DPT ligands are successfully incorporated as separated and protected units, whose emission ability is insensitive to the nanocrystal environment and aggregation-induced losses^43, 53^. The absence of significant reabsorption is further highlighted by low temperature experiments. By cooling the composite down to 10 K, we still observe the first vibronic replica together with a simultaneous increment of the global green emission intensity (+19%, Fig. 4c). This increment is ascribed to the suppression of the intramolecular vibrational quenching mechanism at low temperature, as indicated by the emission lifetime that increases up to 12.5 ns at 10 K (+15%, Fig. 4b, bottom), thus demonstrating the absence of reabsorption-related losses. A more peculiar dynamic is observed for the radioluminescence blue component peaked at 430 nm. At 10 K, we observe a refinement of the vibronic structure in the residual DPA emission, as well as the expected slight lifetime increment (Fig. 4b, top)^54^. However, we observe the simultaneous growth of an overlapped component at 410 nm, which is the main contributor of the blue emission intensity increment, and a UV emission peaked at 280 nm. As showed in the Supplementary Fig. 54, both these components are competitive emission channels ascribed to the host PDMS. We also notice that these electronic transitions are completely dark at 300 K, thus we speculate that they can represent one of the main dissipative pathways that limit *ϕ*_*scint*_ due to a non complete energy transfer from the host matrix to the embedded nanocrystals^55^.

The scintillation of large Stoke shift nanocomposites is investigated in a bulky cylindrical specimen (diameter 1 cm, height 0.5 cm) loaded with Zr-DPT:DPA-8% (0.5% weight) irradiated with a pulsed X-ray beam (Methods). The expected emission output is reported in Fig. 4d, obtained by simulating the propagation of photons in the composites including the scattering and reabsorption/reemission of traveling photons. The model reproduces a scintillation measurement where a photodetector is coupled by an index-matching on the largest face of the scintillator. The scattering is simulated as an artificial constant absorption background that is superimposed on the material absorption spectrum (Methods). The parameter *β* is the geometrical detection efficiency, i.e., the fraction of scintillation photons that reach the photodetector with respect to the total number. The obtained results show that the efficiency of the scintillation light outcoupling at low scattering levels (*β* = 0.31) is significantly improved with respect to the composite based on the *homo*-ligand MOF Zr-DPA (*β* = 0.19). Notably, by exploiting the large Stokes shift nanoscintillators even in very extreme conditions with a scattering as large as 20 cm^−1^, i.e., in the case of fabrication issues that result in an almost completely non-transparent item, the *β* value assesses to 0.22, thus highlighting the improvement achieved with the proposed material engineering strategy.

**Fig. 5:**
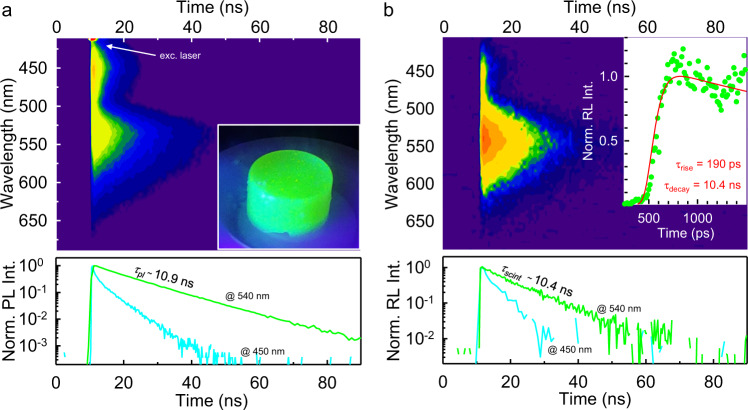
Scintillation properties of large Stokes shift MOF-based nanocomposites. **a** 2D map of the time resolved photoluminescence (PL) of the composite under 405 pulse laser excitation. The inset is a digital picture of the composite under UV lamp exposure. The bottom panel shows the decays in time of the PL intensity recorded at 540 nm and 450 nm. **b** 2D map of the composite scintillation emission under pulsed X-rays exposure. The bottom panel highlight the decay in time of the scintillation pulse intensity recorded at 540 nm and 450 nm. The top panel inset shows the scintillation pulse recorded in the ultrafast time scale with the instrumental response function (IRF) and the fit of the data with a pulse function convoluted with the IRF with a given average decay time *τ*_*decay*_ of 10.4 ns.

Figure 5a reports the 2D photoluminescence map of the specimen. Both the spectrum and the photoluminescence intensity decay under pulsed excitation at 405 nm exhibit the same features of the nanocrystal dispersion, i.e., a main emission peak at 540 with lifetime *τ*_*pl*_ = 10.9 ns and a residual faster and weaker blue emission showing a multi-exponential decay behavior that reaches the ns time scale. The 2D scintillation map is showed in Fig. 5b. The emission spectrum is analogous to the photoluminescence, with an intense scintillation band peaked at 540 nm and a weak blue emission at 430 m and the same recombination kinetics. More precisely, the green scintillation characteristic lifetime *τ*_*scint*_ = 10.4 ns matches well the *τ*_*pl*_ value, thus demonstrating that the MOFs preserve their excellent emission properties also if used as nanoscintillators. Taking a closer look at the ultrafast time scale, we observe that the green scintillation flash is well reproduced by a pulse function with an average decay time *τ*_*decay*_ = 10.4 = *τ*_*scint*_ ns convoluted with the instrumental response function (Methods, inset of Fig. 5b). The scintillation pulse shows a rise time *τ*_*rise*_ of 190 ps, defined as the time variation between 10% and 90% of the maximum pulse intensity. Considering the speed of the energy transfer observed, an accurate estimate of the real rise time is therefore partially hindered by the instrumental response. However, no slow rise time is observed, thus demonstrating that the green scintillation is activated by the fast non-radiative energy transfer, with no contribution from the residual slow blue emitters.

These findings demonstrate the success of the designed strategy of mixed acenes in MOFs and, despite the scintillation rise time cannot be accurately quantified, they suggest that even faster activation kinetics can be achieved. For example, by employing complementary ligands with better energetic resonance or by developing high-diffusivity nanocrystals by the fine tuning of the intermolecular orientation in the MOF framework, it would be possible to further enhance the energy transfer rate, thus achieving the activation of the Stokes shifted luminescence in times below the 100 ps threshold. These emitters will be therefore the ideal building blocks to realize high-optical quality bulk composite systems exploiting optimized loading strategies, which will enable to increase the amount of embedded nanoscintillators avoiding aggregation and therefore limiting the scattering of the scintillation light also in large area devices.

## Discussion

In conclusion, we successfully engineered the composition of co-assembled hetero-ligand MOF nanocrystals obtaining efficient and fast emitters with a large Stokes shift. Thanks to the fast diffusion-mediated non-radiative energy transfer mechanism occurring in the highly ordered crystalline framework between the different species of fluorescent ligands, we achieved a significant Stokes shift as large as 750 meV. This makes these new nanoemitters ideal candidates for reabsorption-free photonic applications that require fast timing response, thus surpassing the current limitation of large Stokes shift semiconductor nanostructures. The excellent emission quantum yield observed is a direct consequence of the success of the synthetic strategy employed to couple complementary, fluorescent acene-based building blocks in a MOF architecture. Indeed, the results indicate that the controlled incorporation of the energy acceptor ligands not only does not perturb the structural properties and stability of the MOFs, but also protect the anthracene-emitters from surface-related luminescence quenching observed in homo-ligand MOF. Moreover, this strategy allows to protect the emitting ligands from the external environment also in a composite preferred form that exploits a transparent polymer host matrix, overcoming the energy losses related to phase segregation and aggregation. The potential impact of these new nanoemitters is highlighted by the investigation of their scintillation properties. The composite plastic scintillator based on hetero-ligand MOFs shows a better scintillation performance with respect to the previously investigated homo-ligand nanocrystals, which already demonstrated interesting features for the development of a new family of fast scintillators. Considering that the proposed strategy can be in principle applied to different ligand pairs with resonant electronic energies to tune both the emission Stokes shift and the spectral working ranges (according to specific application requests), the obtained results strongly support the future development of ligand-engineered MOF nanocrystals for photonic and photo-chemical applications that require energy harvesting, site-specific collecting as well as photon frequency manipulation.

## Methods

### Synthesis of Zr-DPT

MOF nanocrystals were synthetized under solvothermal conditions modulated by acetic acid. The conditions were optimized to generate a highly crystalline sample. Briefly, ZrCl_4_, 5,12-bis(4-carboxyphenyl) tetracene (DPT) and acetic acid were dispersed in DMF (see SI for further details). The resulting mixture was heated at 120 °C for 22 h and the orange powder was filtered and washed with fresh solvent before activation at 130 °C under high vacuum before characterization. The synthesis of DPT ligand is reported in Supporting Information.

### Synthesis of Zr-DPT:DPA-x%

Nanocrystalline samples of Zr-DPT:DPA-x% were synthetized under solvothermal conditions modulated with acetic acid. ZrCl_4_ and a proper amount of 5,12-bis(4-carboxyphenyl)tetracene (DPT) and 9,10-bis(4-carboxyphenyl) anthracene (see SI for further details) were dispersed in a mixture of DMF and acetic acid. The mixture was heated at 120 °C for 22 h and the yellowish powder was filtered and washed with fresh solvent before activation at 130 °C under high vacuum.

### Synthesis of Zr-MOF:PDMS composites

PDMS nanocomposites were prepared by dispersing MOF nanocrystals in a prepolymer mixture that was poured in a proper mould and cured at 60 °C to obtain self-standing nanocomposites. The nanocomposites were obtained by the reaction of vinyl-terminated polydimethylsiloxane with polydimethylsiloxane-*co*-methylhydrosiloxane by thermal curing. The cross-linking reaction starting from the polymer terminals preserved the flexibility of the polymer chains and produced very low glass transition^56^.

### Structure analysis and microscopy

The structure and composition of Zr-MOF nanocrystals and nanocomposites were determined by means of powder X-ray diffraction (PXRD) structure refinement, nuclear magnetic resonance (NMR) spectroscopy, Fourier - transform infrared (FT-IR) spectroscopy, thermogravimetric analysis (TGA), adsorption properties, helium picnometry, scanning electron microscopy (SEM) and transmission electron microscopy (TEM). Details on the instrumental setup and the measurement protocols are reported in the Supplementary Information file. The crystal structures were refined by the Rietveld method combined with molecular mechanics and plane-wave DFT calculations (see Supplementary Information).

### Photoluminescence studies

All experiments have been performed on activated nanocrystals dispersions in tetrahydrofuran (THF). Absorption spectra has been recorded with a Cary Lambda 900 spectrophotometer at normal incidence using quartz Suprasil cuvettes with 0.1 cm of optical path and an integrating sphere to eliminate scattering effects. Steady state photoluminescence (PL) spectra were acquired with a Variant Eclipse fluorimeter (bandwidth 1 nm) using quartz Suprasil cuvettes with 1 cm of optical path. Time-resolved photoluminescence experiments in the nanosecond time scale have been made by using as excitation source a pulsed laser LED at 340 nm (3.65 eV, EP-LED 340 Edinburgh Instruments, pulse width 80 ps) coupled to FLS1000 Edinburgh setup in Time-Correlated Single Photon Counting (TCSPC) acquisition mode. Quartz Suprasil cuvettes with 0.1 cm of optical path has been used to study nanocrystals dispersions. The nanocomposites were excited with a pulsed laser at 405 nm (3.06 eV, EPL-405 Edinburgh Instruments, pulse width 90 ps) to avoid direct excitation of the host polymer matrix, coupled to FLS1000 Edinburgh setup in Time-Correlated Single Photon Counting (TCSPC) acquisition mode. Time resolved experiments in the picoseconds time scale (Fig. 3c) have been made by using a doubled Ti:Sapph pulsed laser at 370 nm (3.65 eV, Coherent Mira 900, pulse width 150 fs) coupled to a streak camera Hamamatsu Synchroscan M1955, which temporal resolution is below 1 ps. The PL quantum yields of DPT and MOF 8% dispersion have been measured by relative methods as described in the Supplementary Information file. Measurements on composites as a function of the temperature were performed on films 0.1 cm thickness and diameter 1 cm, mounting the sample in closed circle He cryostat with direct optical access.

### Radioluminescence studies

Steady state RL measurements were carried out at room temperature using a homemade apparatus featuring, as a detection system, a liquid nitrogen-cooled, back-illuminated, and UV-enhanced charge coupled device (CCD) Jobin-Yvon Symphony II, combined with a monochromator Jobin-Yvon Triax 180 equipped with a 100 lines/mm grating. All spectra are corrected for the spectral response of the detection system. RL excitation was obtained by unfiltered X-ray irradiation through a Be window, using a Philips 2274 X-ray tube with tungsten target operated at 20 kV. At this operating voltage, a continuous X-ray spectrum is produced by a Bremsstrahlung mechanism superimposed to the L and M transition lines of tungsten, due to the impact of electrons generated through thermionic effect and accelerated onto a tungsten target. The dose rate was 0.2 Gy/s, evaluated by comparison with a calibrated ^90^Sr-^90^Y beta radioactive source and using optically stimulated luminescence emission from quartz crystalline powder (100 – 200 μm grains). In order to record the PL measurements, the same acquisition system of RL measurements has been coupled to a 405 nm pulsed diode laser (EPL-405 Edinburgh Instruments) through a quartz optical fiber bundle allowing the illumination of the sample in the X-ray chamber.

### Scintillation studies

Pulsed X-rays with energies up to 25 keV were generated with a repetition rate of 1 MHz by a picosecond diode laser at 405 nm (Delta diode from Horiba) focused on a X-ray tube (model N5084 from Hamamatsu). In the case of optical excitation, the same laser (405 nm) was used. The resulting photons were collected by Kymera spectrograph (ANDOR) and detected by a hybrid PMT 140-C from Becker & Hickl GmbH. For decay-time measurements, the photons were histogramed using a PicoHarp300 time-correlated single-photon counting (32 ps time/bin) and for the time resolved spectra a MCS6A multiple-channel time analyzer was used (800 ps time/bin) Sub-nanosecond scintillation emission kinetics of the samples were measured with a Time Correlated Single Photon Counting (TCSPC) setup. As excitation source a pulsed X-Ray beam (X-ray Tube XRT N5084, Hamamatsu), with a continuous energy spectrum between 0 and 40 keV and a mean energy of 9.15 keV, produced with a Pulse Diode Laser (PDL 800-B, PicoQuant) were used. The scintillation light was collected in reflection by a Hybrid Photomultiplier Tube (HPM 100-07, Becker & Hickel), operating in TCSPC mode, and processed by an Amplifier and Timing Discriminator (model 9237, ORTEC). This processed HPM output signal was used as stop signal for a Time to Digital Converter (TDC xTDC4, chronologic), while the start signal was given by the external trigger of the PDL. An optical band-pass filter (450 nm with a FWHM of 40 nm) was used, chosen accordingly to the emission spectrum of the samples, to cut observed air excitation by X-Ray. The scintillation pulse was fitted with a convolution between the Impulse Response Function (IRF) of the whole system with a full width at half maximum (FWHM) of 180 ps and the intrinsic scintillation rate^9^.

### Light propagation modeling

Simulations of the scintillating nanocomposite performances were carried out using a Monte Carlo ray-tracing method previously presented^43^. The photon propagation follows geometrical optics laws where the interference is neglected. Each photon can be absorbed and re-emitted by a chromophore, isotropically scattered, and reflected or transmitted at the interfaces, where the Fresnel coefficients have been used to compute the reflection probability. The simulated scintillator contains the same number of nanocrystals employed to fabricate the described sample. The absorption, scattering, transmission, or reflection events are chosen according to random Monte Carlo drawing. The simulations were performed using the experimental absorption/luminescence spectrum and emission efficiency of nanocrystals (Φ_PL_ = 67% for Zr-DPT:DPA-8% and Φ_PL_ = 27% for Zr-DPA). The scattering is supposed to induce light attenuation corresponding to an absorption coefficient ranging from 0 to 20 cm^−1^.

## Supplementary information

supplementary information file

Description of Additional Supplementary Files

Supplementary Data File 1

## Data Availability

All the data reported in the plots and other findings of this study reported in the main paper and in the Supplementary Information are available from the corresponding author upon request. The raw data on Zr-DPT:DPA-0.x% structures reported in Supplementary Fig. 36 are available in Supplementary Data File 1. The X-ray crystallographic coordinates for the Zr-DPT and Zr-DPA structures reported in this study have been deposited at the Cambridge Crystallographic Data Centre (CCDC), under deposition numbers 2131110 and 2000249 respectively. These data can be obtained free of charge from The Cambridge Crystallographic Data Centre via www.ccdc.cam.ac.uk/data_request/cif.
