# Supplementary material for: Highly luminescent scintillating hetero-ligand MOF nanocrystals with engineered Stokes shift for photonic applications
Source: Nat Commun. 2022 Jun 17;13:3504. doi: 10.1038/s41467-022-31163-0 (PMC9205964; doi:10.1038/s41467-022-31163-0)
Supplement: Supplementary file 1 — supplementary information file [file 41467_2022_31163_MOESM1_ESM.pdf]

Supplementary Information for:

Highly luminescent scintillating hetero-ligand MOF nanocrystals with engineered Stokes shift for photonic applications.

J. Perego[1], Charl X. Bezuidenhout[1], I. Villa[1,4], F. Cova[1], R. Crapanzano[1], I. Frank[3,5], N. Kratochwill[3,6], F. Pagano[3,2], E. Auffray[3], S. Bracco[1], A. Vedda[1], C. Dujardin[7], P. E. Sozzani[1], F. Meinardi[1], A. Comotti[1] and A. Monguzzi[1]**

[1] Dipartimento di Scienza dei Materiali, Università degli Studi Milano-Bicocca, via R. Cozzi 55, 20125 Milano – ITALY

[2] Dipartimento di Fisica "Giuseppe Occhialini", Università degli Studi Milano-Bicocca, Piazza della Scienza 3, 20126 Milano – ITALY

[3] CERN, Geneva, Switzerland

[4] FZU Institute of Physics, Academy of Sciences of the Czech Republic, Prague – Czech Republic

[5] Ludwig Maximilian University of Munich, Geschwister-Scholl-Platz 1, Munich, Germany

[6] University of Vienna, Vienna, Austria

[7] Univ. Lyon, Université Claude Bernard Lyon1, CNRS, Institut Lumière Matière, F-69622, France

E-mail: angiolina.comotti@unimib.it , angelo.monguzzi@unimib.it

INDEX

1. EXPERIMENTAL METHODS page 3

2. Ligand synthesis page 4

2.1 Synthetic procedure for 5,12-bis(4-carboxyphenyl) tetracene (DPT) page 6

2.2 Liquid NMR characterization page 8

2.3 Infrared spectroscopy page 13

2.4 Photophysical properties of ligands page 14

3. *Homo*-ligand Zr-MOFs page 15

3.1 Synthetic procedure for Zr-DPT page 15

3.2 Infrared spectroscopy page 15

3.3 ^{13}C CP MAS and ^{1}H MAS solid state NMR of Zr-DPT page 16

3.4 SEM images and particle size distribution page 19

3.5 Powder x-ray diffraction and crystal structure of Zr- DPT page 20

3.6 Nitrogen adsorption isotherm at 77 K and sorption properties page 24

4. *Hetero*-ligand MOFs: Zr-DPT:DPA-x% page 25

4.1 Synthetic procedure for Zr-DPT:DPA-x% page 25

4.2 Digital image of MOF samples page 25

4.3 ^{1}H NMR of digested samples page 26

4.4 Infrared spectroscopy of Zr-DPT:DPA-x% page 27

4.5 ^{13}C CP MAS, ^{1}H MAS and 2D ^{1}H-^{13}C PMLG HETCOR solid state NMR page 28

4.6 Thermogravimetric analysis (TGA) page 30

4.7 SEM images and particle size distribution page 31

4.8 Powder x-ray diffraction and crystal structure of Zr-DPT-DPA-x% page 33

4.9 Nitrogen adsorption isotherms at 77 K and sorption properties page 37

5. Exciton diffusion/energy transfer modeling and supplementary data page 40

6. Zr-DPT:DPA-8%:PDMS nanocomposite page 46

6.1 Synthetic procedure for Zr-DPT:DPA-8%:PDMS composite page 46

6.2 Digital image of samples under ambient and UV light page 46

6.3 Differential scanning calorimetry (DSC) of Zr-DPT:DPA-8%:PDMS composite page 47

6.4 Infrared spectroscopy of Zr-DPT:DPA-8%:PDMS composite page 47

6.5 ^{13}C CP MAS spectrum of the nanocomposite Zr-DPT:DPA-8%:PDMS page 48

7. NANOCOMPOSITES SCINTILLATION PROPERTIES: supplementary data page 49

8. SUPPLEMENTARY REFERENCES page 52

1. EXPERIMENTAL METHODS

Nuclear magnetic resonance (NMR) spectroscopy. ^{1}H-NMR spectra were recorded on a AVANCE NEO Bruker instrument (400 MHz) at 298.1 K. MOFs samples were digested with deuterated trifluoroacetic acid (TFA_d, 0.15 mL) and the solution was diluted with deuterated dimethyl sulfoxide (DMSO_d_6, 0.8 mL).

Fourier transform infrared (FTIR) spectroscopy. FTIR spectra measurements were performed with a Jasco FT/IR 4100 equipped with an ATR PRO450-S module. MOFs samples were evacuated under high vacuum at 130°C before analysis to ensure the complete removal of adsorbed water vapor and guest species. Spectra were collected between 600 cm^{-1} and 4000 cm^{-1} with resolution of 2.0 cm^{-1}.

Thermogravimetric analysis (TGA). TGA were performed using a Mettler Toledo Star System 1 equipped with a gas controller GC10. The experiments were conducted applying a ramp from 30°C to 1000°C and a scan rate of 10°C/min in dry air.

Scanning electron microscopy (SEM). Scanning electron microscopy (SEM) images were collected using a Zeiss Gemini 500 microscope, operating at 5 KV and a working distance of 2.9 mm. Samples were casted on a silicon slide from 2-propanol dispersion, dried under high vacuum and sputtered with chromium before the analysis. Particle size distributions were measured from SEM images using ImageJ software. The linear dimensions of nanocrystals have been manually evaluated for more than 100 particles to determine the particle size distributions.

Gas adsorption properties. N_2 adsorption isotherms at 77 K were collected up to 1 bar using a Micromeritics analyzer ASAP2020 HD. Samples were previously outgassed overnight at 130 °C under high vacuum (10^{-3} mmHg) to remove the adsorbed species. Low temperature (77 K) nitrogen adsorption isotherms were fitted using Langmuir and BET models and surface areas were calculated in the range from 0.015 to 0.1 p/p°. Pore size distributions (PSD) were calculated according to non-local density functional theory (NLDFT) model.

Solid-state NMR Spectroscopy. ^{13}C solid-state NMR experiments were carried out with a Bruker Avance 300 instrument operating at a static field of 7.04 T equipped with high-power amplifiers (1 kW) and a 4 mm double resonance MAS probe. ^{13}C ramped-amplitude cross polarization (CP) experiments were performed at a spinning speed of 12.5 kHz using a recycle delay of 5 s and a contact time of 2 ms. The 90° pulse for proton was 2.9 μs. Quantitative ^{13}C single pulse (^{13}C SPE) MAS NMR experiment was performed at a spinning speed of 12.5 kHz with a recycle delay of 30 s and a 90° pulse of 3.6 μs length. Crystalline polyethylene was taken as an external reference at 32.8 ppm from TMS. Quantitative ^{1}H single pulse (^{1}H SPE) MAS NMR experiment was performed at a spinning speed of 12.5 kHz with a recycle delay of 20 s and a 90° pulse of 2.5 μs. ^{1}H chemical shift was referenced to adamantane.

Phase-modulated Lee−Goldburg (PMLG) heteronuclear ^{1}H-^{13}C correlation (HETCOR) experiments coupled with fast magic angle spinning allowed the recording of the 2D spectra with a high resolution in both hydrogen and carbon dimensions. Narrow hydrogen resonances, with line widths on the order of 1−2 ppm, were obtained with homonuclear decoupling during t_1; this resolution permits a sufficiently accurate determination of the proton species in the system. The 2D ^{1}H - ^{13}C PMLG HETCOR spectra were run with an LG period of 18.9 μs. The efficient transfer of magnetization to the carbon nuclei was performed by applying the RAMP-CP sequence. Quadrature detection in t_1 was achieved by the time proportional phase increments method (TPPI). The carbon signals were acquired during t_2 under proton decoupling by applying the two-pulse phase modulation scheme (TPPM). The 2D ^{1}H-^{13}C PMLG HETCOR NMR spectrum of Zr-DPT:DPA-8% was collected at 298 K under magic-angle spinning (MAS) conditions at 12.5 kHz with a contact time of 2 ms.

Hyperpolarized ^{129}Xe NMR Spectroscopy. Hyperpolarized ^{129}Xe NMR experiments were performed by a home-built apparatus with a continuous-flow delivery of hyperpolarized xenon gas with a Bruker Avance 300 spectrometer operating at a Larmor Frequency of 83.02 MHz for ^{129}Xe. A diode array laser delivering 6 W at 795 nm was applied, circular polarization was achieved using a beam splitting cube and quarter wave plate. A stream of gas mixture containing 2% xenon, 2% nitrogen and 96% helium at

2 atm was used and the gas flow rate was maintained at 20 L/h. The samples were outgassed overnight at 120°C in vacuum, then were pressed in a glass tube before being inserted into the coil. A pulse duration of 7 μs was applied, with a recycle delay of 0.5 s. The ^{129}Xe NMR chemical shifts were referenced to xenon gas set at 0 ppm.

Powder x-ray diffraction (PXRD) structure refinement. PXRD measurements were accomplished with a Rigaku powder diffractometer using Cu-Kα radiation, 40 kV, 30 mA over a range of 2θ = 2.0-80.0 with a step size of 0.02° and a scan speed of 0.3 °/s. The Zr-DPT model for the Rietveld refinement was generated from Zr-DPA structure, [1] considering an extra phenyl ring to complete the tetracene moiety. This extra ring can be in four position and was thus modeled with an occupancy of 0.25 over four sites. All Rietveld structural refinements of the X-ray data were performed using the TOPAS-Academic64 V6 software package. [2,3] For the final Rietveld refinement, the structure was modelled as a disordered system with a symmetry of *Fm-3m*. The Zr-DPA structure[1] was also used as input structure for the PXRD refinement of the systems with low percentages of DPT ligand present. During the Rietveld refinement the structure was modelled as a disordered system with a symmetry of *Fm-3m*. The background was fitted and refined using a Chebyshev polynomial with 20 coefficients in the range of the PXRD trace from 2° to 80° 2theta and the application of baseline shift refinement. Other corrections include: Specimen Displacement, Divergence Sample Length, Absorption with Sample Thickness Shape Intensity and Specimen Tilt. The peaks were fitted using a pseudo-Voigt profile. Preferred orientation was considered using an eighth order Spherical Harmonics refinement. The refinement was performed for the PXRD traces of **Zr-DPA:DPT-0.1%**, **Zr-DPA-DPT-0.5%**, **Zr-DPA-DPT-1%** and **Zr-DPA-DPT-8%**. This illustrates quantitatively that the structures with various percentages of DPT are still consistent with the original Zr-DPA crystal structure.

General Computational Details for Materials Studio Software Suite. [4] The Zr-DPA structure[1] was used as input structure for the PXRD refinement. The unit-cell dimensions and all atomic coordinates were optimized as part of a periodic system in *P*-1 using the CASTEP module of the Materials Studio software suite. The optimizations were performed using the GGA PBE functional with Grimme's DFT-D dispersion correction, and thresholds for geometry optimization and SCF convergence were chosen as 2×10^{-6} eV. The combination of the two models were taken for the final optimization in the *Pc* space group. For the Rietveld refinement, the structure was modelled as a disordered system in the *Fm*-3*m* space group.

2. Ligand synthesis

All reagents were purchased from Merck and used without further purification. Tetrahydrofuran (THF) was distilled from sodium/benzophenone under nitrogen atmosphere before use. Gaseous CO_2 (4.5 purity) was employed for the ligand synthesis.

Linker synthesis. The synthesis of organic linker 9,10-bis(4-carboxyphenyl) anthracene (DPA) was performed as reported below following the literature procedure[1].

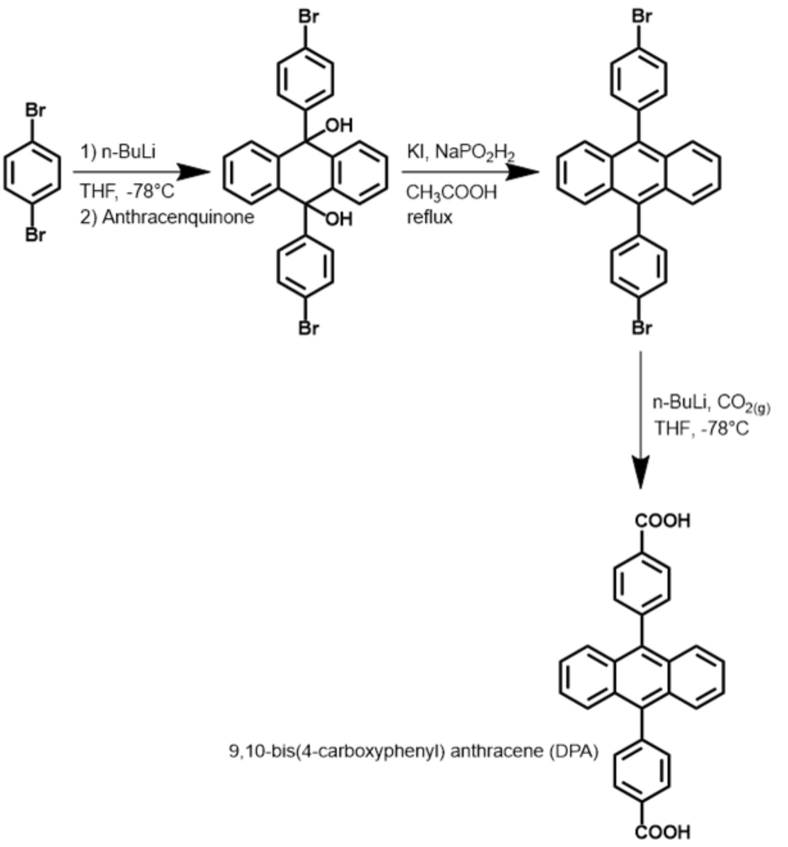

Synthetic procedure for 9,10-bis(4-carboxyphenyl) anthracene (DPA).

Synthesis 9,10-bis(4-carboxyphenyl) anthracene (DPA). Briefly, n-butyllithium (4.8 mL; 12.0 mmol; 2.5 M in hexane) was added dropwise to a solution of 1,4-dibromobenzene (2.83 g; 12.0 mmol) in dry THF (100 mL) at -78°C under nitrogen atmosphere. The resulting solution was stirred for 30 minutes at -78°C and then a dispersion of anthraquinone (1.19 g; 5.70 mmol) in 150 mL of dry THF was added *via* cannula under inert atmosphere using a Schlenk line. The resulting dispersion was stirred at -78 °C for other 2 hours and allowed to warm to room temperature overnight. The solvent was removed by rotatory evaporation and the solid was recovered with Et_2O and washed with deionized water. The organic fractions were collected and the solvent was removed under vacuum to yield an off-white powder. The product was recrystallized from hot toluene to give colourless crystals (2.11 g). Yield: 34%. The diol (2.1 g; 4 mmol) was added to a round bottom flask containing KI (4.1 g; 24.7 mmol), $NaPO_2H_2{\cdot}H_2O$ (4.6 g; 52.3 mmol) and 40 mL of acetic acid and the mixture was heated to reflux for 2 hours. The precipitation of a yellow powder was observed. The dispersion was cooled down to room temperature and the solid was collected by suction filtration and washed with acetic acid (20 mL) and then deionized water (50 mL) and dried in air overnight. The product was recrystallized from CH_2Cl_2 to obtain slightly greenish crystals (1.67 g). Yield: 85%. 9,10-bis(4-bromophenyl)anthracene (0.7 g; 1.43 mmol) was dissolved in 70 mL of dry THF and the solution was cooled to -78 °C. n-butyllithium (2 mL ; 5 mmol; 2.5 M in hexane) was added dropwise under inert atmosphere and the solution was further stirred for 45 minutes at -78°C. Gaseous carbon dioxide was bubbled in the resulting solution while the mixture was slowly allowed to warm to room temperature. After 3 hours the bubbling was interrupted, THF (40 mL) and 1M HCl aqueous solution (20 mL) were added to precipitate a yellowish solid (pH ~ 1). The powder was filtered and washed with deionized water. The product was further crystallized from THF/hexane mixture to give off-white crystals (0.5 g). Yield: 84%. ^1H NMR (400MHz, DMSO_d_6): δ (ppm) = 13.15 (2H, s), 8.23 (4H, d), 7.61 (4H, d), 7.55 (4H, m), 7.45 (4H, m).

2.1 Synthetic procedure for 5,12-bis(4-carboxyphenyl) tetracene (DPT)

The organic linker 5,12-bis(4-carboxyphenyl) tetracene was synthetized according to the following scheme.

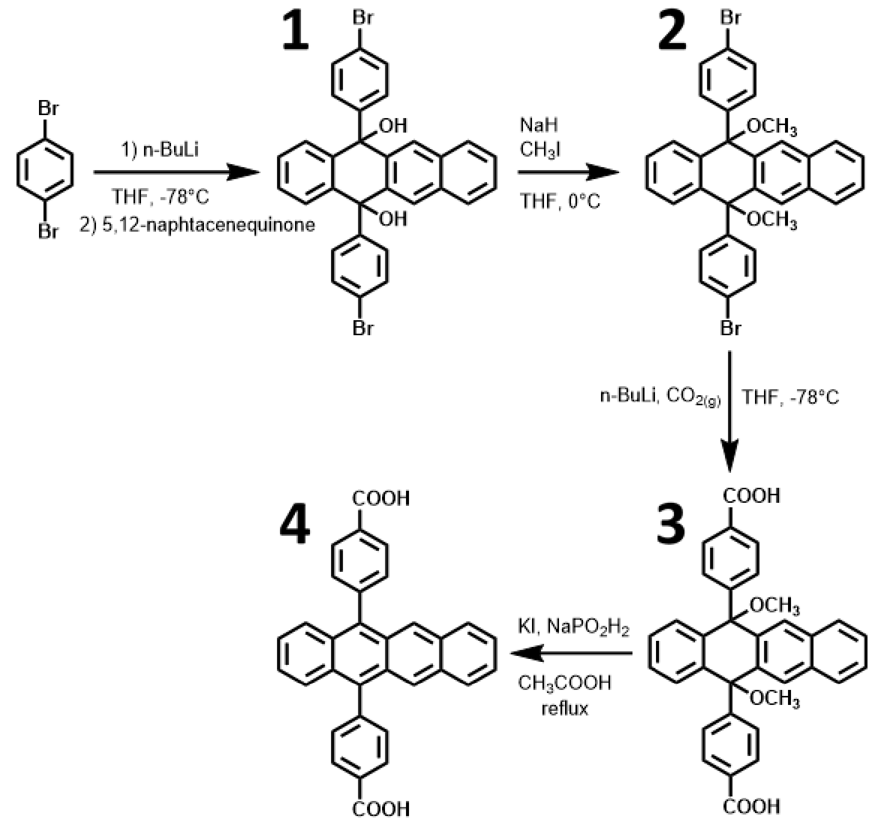

Synthetic procedure for 5,12-bis(4-carboxyphenyl) tetracene (DPT, **4**).

Synthesis of 5,12-bis(4-bromophenyl)-5,12-dihydronaphtacene (1). *n*-butyllithium (4.8 mL; 12.0 mmol; 2.5 M in hexane) was added dropwise to a solution of 1,4-dibromobenzene (2.83 g; 12.0 mmol) in dry tetrahydrofuran (100 mL) at -78°C (dry ice/acetone bath) under nitrogen atmosphere. The resulting solution was stirred for 30 minutes at the same temperature and then a dispersion of 5,12-naphtacenequinone (1.47 g; 5.70 mmol) in 150 mL of dry tetrahydrofuran was added *via* cannula. The resulting dispersion was stirred at -78 °C for 2 hours and allowed to warm to room temperature overnight. The solvent was removed by rotatory evaporation and the solid was recovered with diethyl ether and washed with water. The organic fractions were collected and the solvent was removed under vacuum to yield an off-white powder. The product was recrystallized from hot toluene to give colorless crystals (2.05 g). Yield: 30 %. The crystalline powder was treated at 90°C under vacuum overnight before IR and NMR characterizations. ^{1}H-NMR (400 MHz, DMSO_d$_6$): δ (ppm) = 6.44 ppm (4H, d), 6.61 ppm (2H, s), 6.91 ppm (4H, d), 7.52 ppm (2H, m), 7.57 ppm (2H, m), 8.01 ppm (2H, m), 8.06 ppm (2H, m), 8.46 ppm (2H, s).

Synthesis of 5,12-bis(4-bromophenyl)-5,12-diomethoxy-naphtacene (2). 5,12-bis(4-bromophenyl)-5,12-dihydronaphtacene (2 g; 3.5 mmol) was dissolved in dry tetrahydrofuran (30 mL) under nitrogen atmosphere. The clear solution was cooled down to 0°C with an ice/water bath and sodium hydride

(60%$_{wt}$ dispersion in mineral oil; 560 mg; 14 mmol) was added under vigorous stirring. After 30 minutes iodomethane (1 mL; 16 mmol) was added dropwise at 0°C and the reaction was stirred for 18 hours at room temperature under inert atmosphere. Then, the reaction was quenched with 2 mL of water. The white precipitate was filtered and washed with fresh THF (30 mL) and deionized water (30 mL) to remove all inorganic byproducts. The white product was dried in air and collected. Liquid ^{1}H NMR spectrum displayed high sample purity and the white product was used for the next reaction without further purification (1.85 g). Yield: 88 %. The crystalline powder was treated at 90°C under vacuum overnight before IR and NMR characterizations. ^{1}H-NMR (400 MHz, $CDCl_3$): δ (ppm) = 2.98 ppm (6H, s), 7.22 ppm (4H, d), 7.31 ppm (4H, d), 7.36 ppm (2H, m), 7.45 ppm (2H, m), 7.59 ppm (2H, m), 7.81 ppm (2H, m), 8.06 ppm (2H, s).

Synthesis of 5,12-bis(4-carboxyphenyl)-5,12-dimethoxynaphtacene (3). 5,12-bis(4-bromophenyl)-5,12-diomethoxy-naphtacene (1 g; 1.67 mmol) was dissolved in 100 mL of dry tetrahydrofuran and the solution was cooled to -78 °C with a dry ice/acetone bath. *n*-butyllithium (2 mL; 5 mmol; 2.5 M in hexane) was added dropwise under inert atmosphere in 10 minutes and the solution was further stirred for 45 minutes at -78°C. Gaseous carbon dioxide was bubbled in the resulting solution while the mixture was slowly allowed to warm to room temperature. After 3 hours the bubbling was stopped, tetrahydrofuran (20 mL) and 1M HCl aqueous solution (20 mL) were added to precipitate the solid as an off-white powder. The powder was filtered and washed with water to remove residual inorganic salts. The product was further crystallized from tetrahydrofuran/hexane to give a white powder (0.5 g). The crystalline powder was treated at 50°C under vacuum overnight before IR and NMR characterizations. Yield: 57%. ^{1}H-NMR (400 MHz, DMSO_d$_6$): δ (ppm) = 2.92 (6H, s), 7.47 (10H, m), 7.85 (4H, d), 7.95 (2H, m), 8.08 (2H, s), 12.86 (2H, s).

Synthesis of 5,12-bis(4-carboxyphenyl) tetracene (DPT) (4). 5,12-bis(4-carboxyphenyl)-5,12-dimethoxy naphtacene (1 g; 1.88 mmol) was dispersed in 40 mL of acetic acid in a round bottom flask containing KI (4.1 g; 24.7 mmol), $NaPO_2H_2 \cdot H_2O$ (4.6 g; 52.3 mmol) and the mixture was heated to reflux for 2 hours. The precipitation of an orange powder was observed. The dispersion was cooled down to room temperature and the solid was collected by filtration and washed with acetic acid (30 mL) and water (30 mL) and dried in air overnight. The product was recrystallized from tetrahydrofuran/hexane (0.685 g). The crystalline powder was treated at 50°C under vacuum overnight before IR and NMR characterizations. Yield: 73%. ^{1}H-NMR (400 MHz, DMSO_d$_6$): δ (ppm) = 7.38 (4H, m), 7.53 (2H, m), 7.69 (4H, d), 7.91 (2H, m), 8.25 (2H, s), 8.28 (4H, d), 13.18 (2H,s). ^{13}C NMR (DMSO_d$_6$) δ (ppm) = 125.01, 125.69, 125.95, 126.19, 127.95, 128.19, 128.44, 129.80, 130.47, 130.78, 131.49, 135.99, 143.05, 167.32.

2.2 Liquid NMR characterization

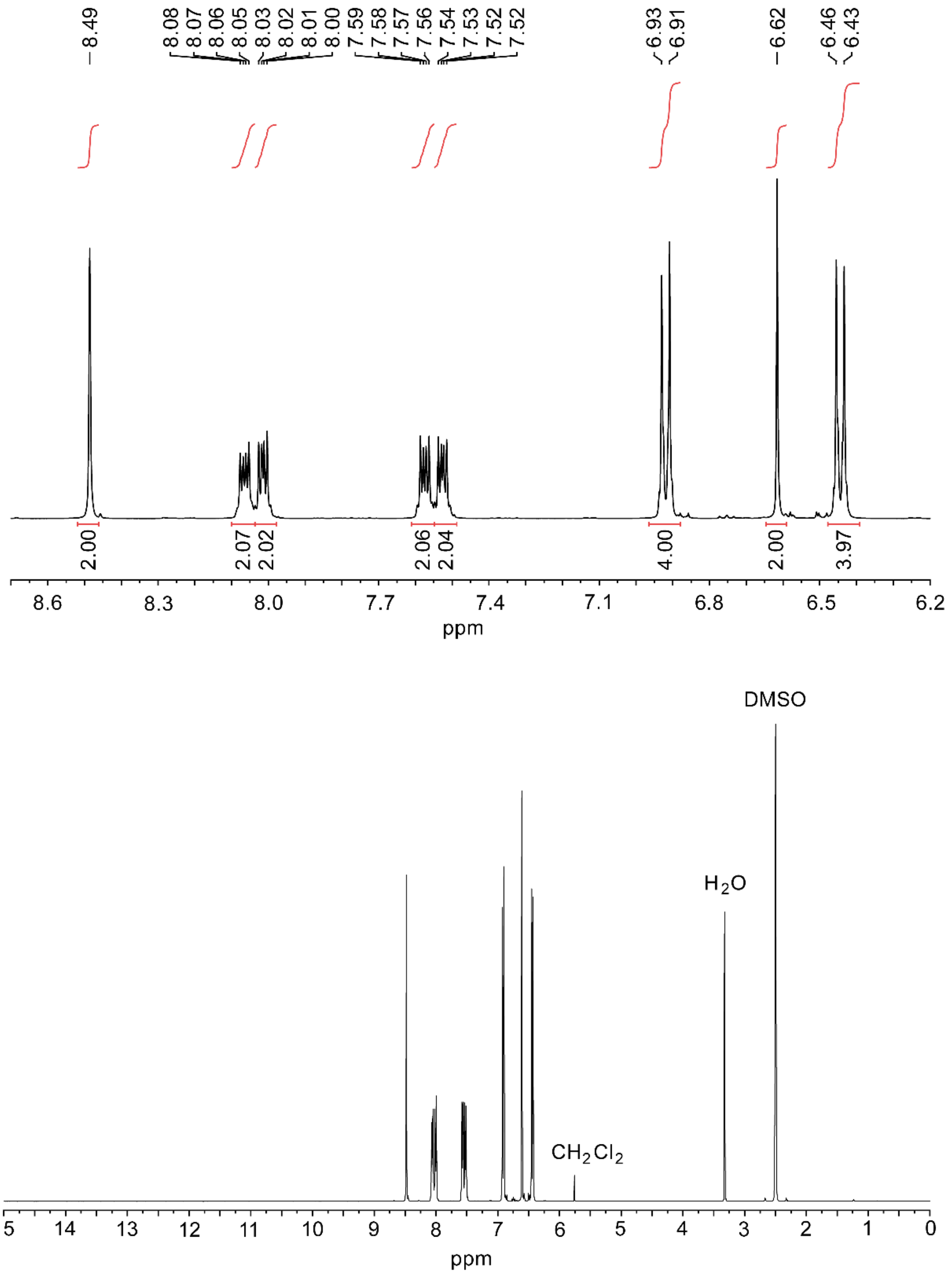

Supplementary Figure 1 | Top: enlargement of the aromatic region of ^1H liquid NMR of 5,12-bis(4-bromophenyl)-5,12-dihydronaphtacene (**1**) in the range between 6.2 ppm and 8.7 ppm. Bottom: ^1H liquid NMR of 5,12-bis(4-bromophenyl)-5,12-dihydronaphtacene (**1**) between 0 ppm and 15 ppm. The spectrum was collected on a AVANCE NEO Bruker instrument (400 MHz) at 298.1 K in DMSO_d6.

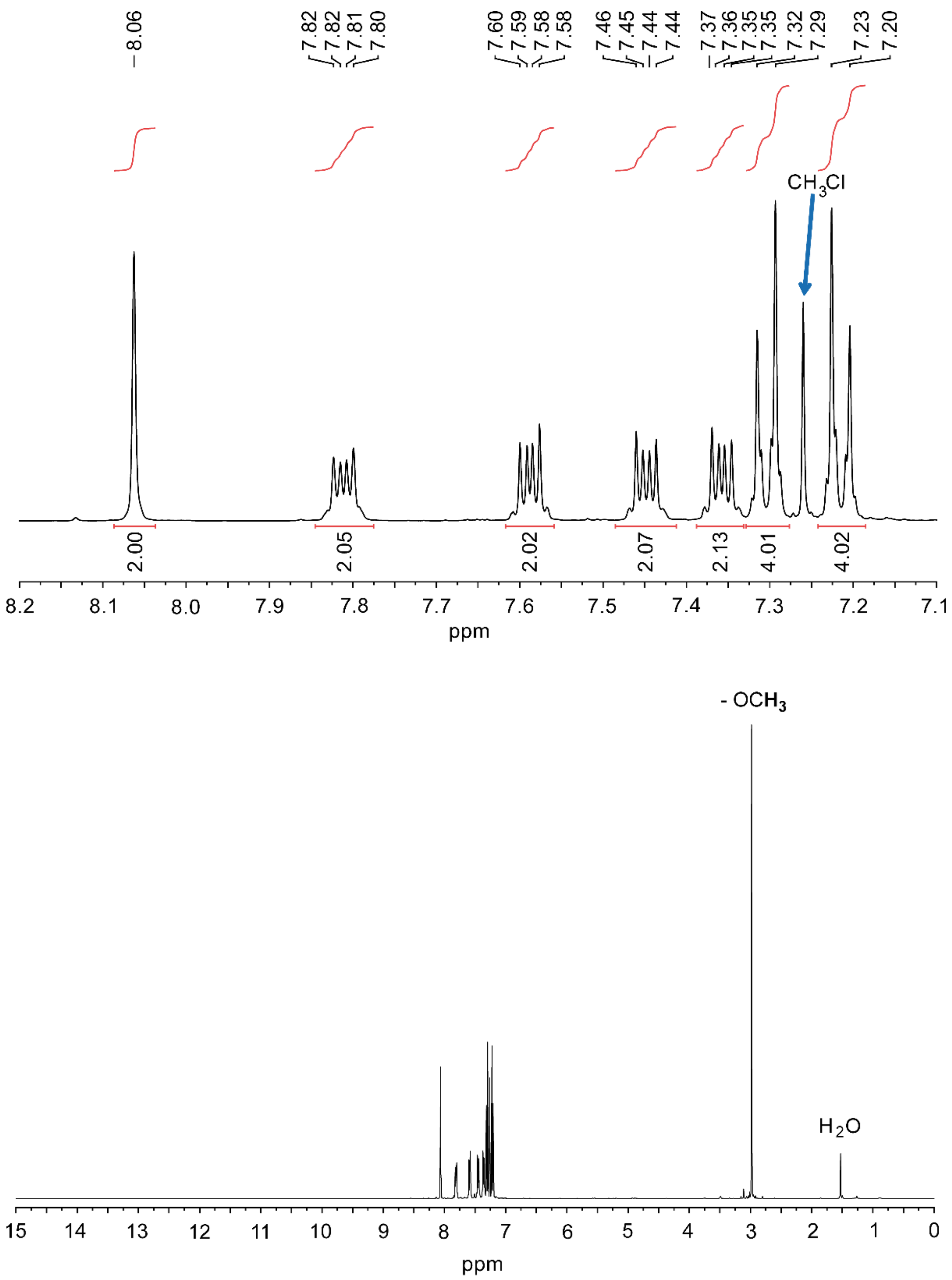

Supplementary Figure 2 | Top: enlargement of the aromatic region of ^{1}H liquid NMR of 5,12-bis(4-bromophenyl)-5,12-diomethoxy-naphtacene (**2**) in the range between 7.1 ppm and 8.2 ppm. Bottom: ^{1}H liquid NMR of 5,12-bis(4-bromophenyl)-5,12-diomethoxy-naphtacene (**2**) between 0 ppm and 15 ppm. The spectrum was collected on a AVANCE NEO Bruker instrument (400 MHz) at 298.1 K in $CDCl_3$.

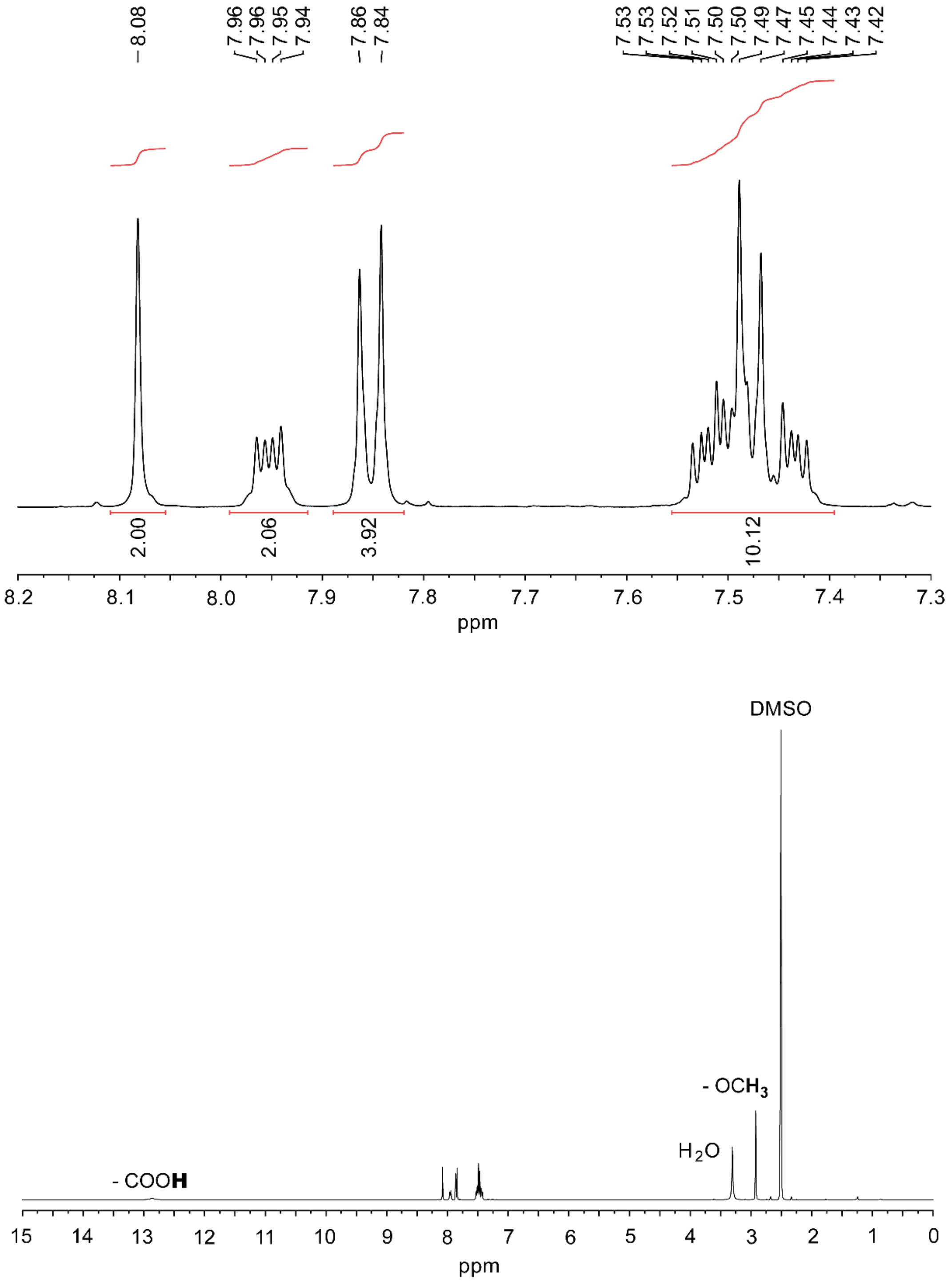

Supplementary Figure 3 | Top: enlargement of the aromatic region of ^{1}H liquid NMR of 5,12-bis(4-carboxyphenyl)-5,12-dimethoxynaphtacene (**3**) in the range between 7.3 ppm and 8.2 ppm. Bottom: ^{1}H liquid NMR of 5,12-bis(4-carboxyphenyl)-5,12-dimethoxynaphtacene (**3**) in the range between 0 ppm and 15 ppm. The spectrum was collected on a AVANCE NEO Bruker instrument (400 MHz) at 298.1 K in DMSO_d6.

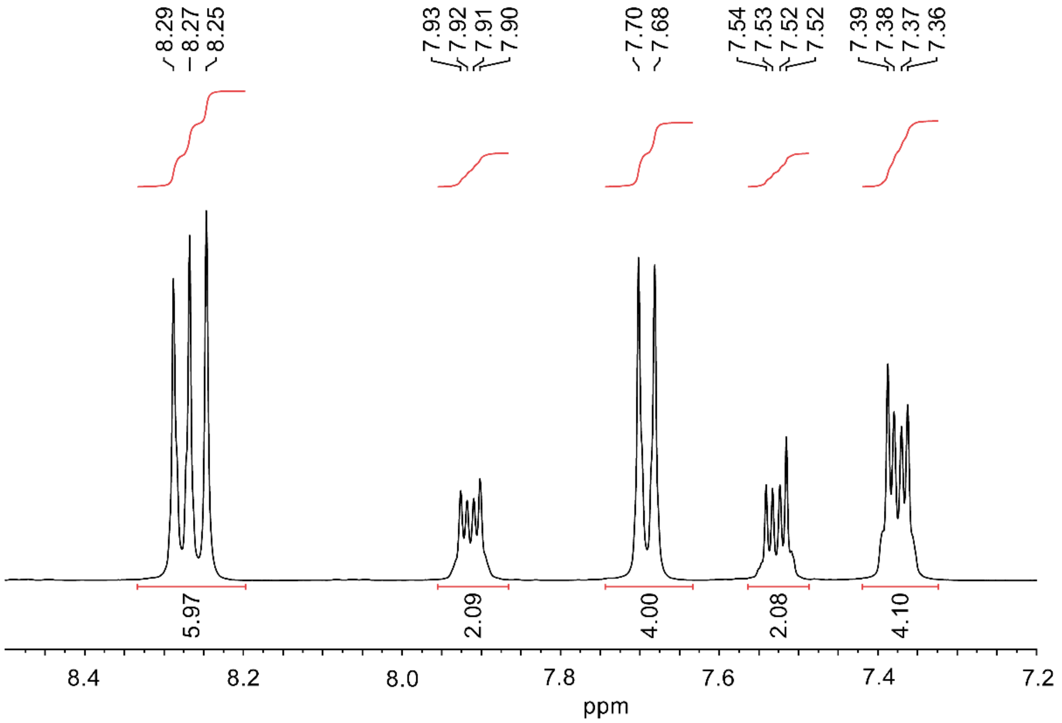

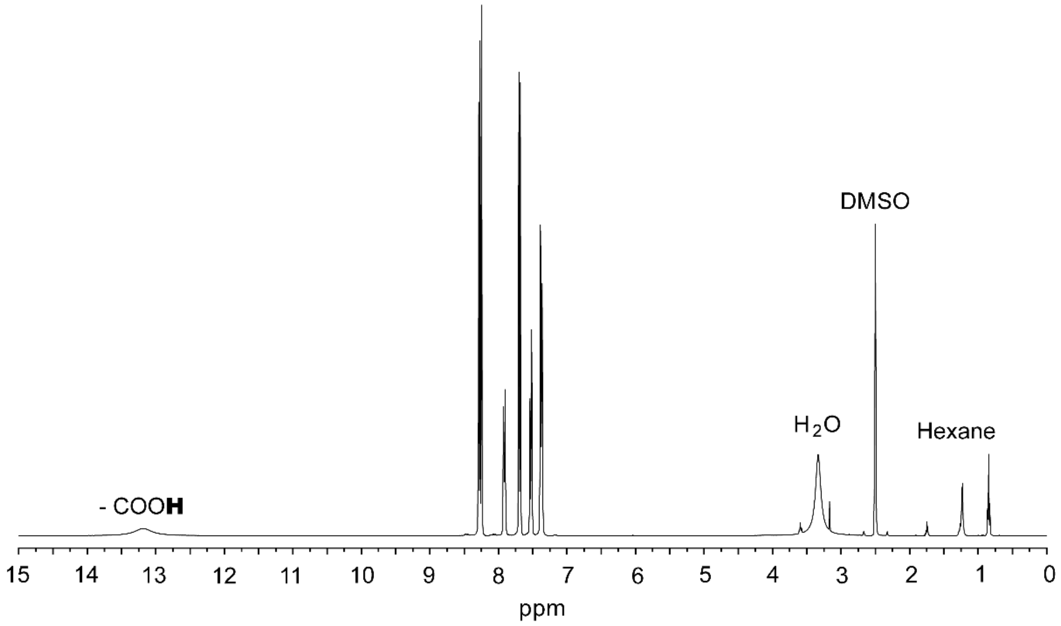

Supplementary Figure 4 | Top: enlargement of the aromatic region of ^{1}H liquid NMR of 5,12-bis(4-carboxyphenyl) tetracene (**4**) in the range between 7.2 ppm and 8.5 ppm. Bottom: ^{1}H liquid NMR of 5,12-bis(4-carboxyphenyl) tetracene(**4**) in the range between 0 ppm and 15 ppm. The spectrum was collected on a AVANCE NEO Bruker instrument (400 MHz) at 298.1 K in DMSO_d_6.

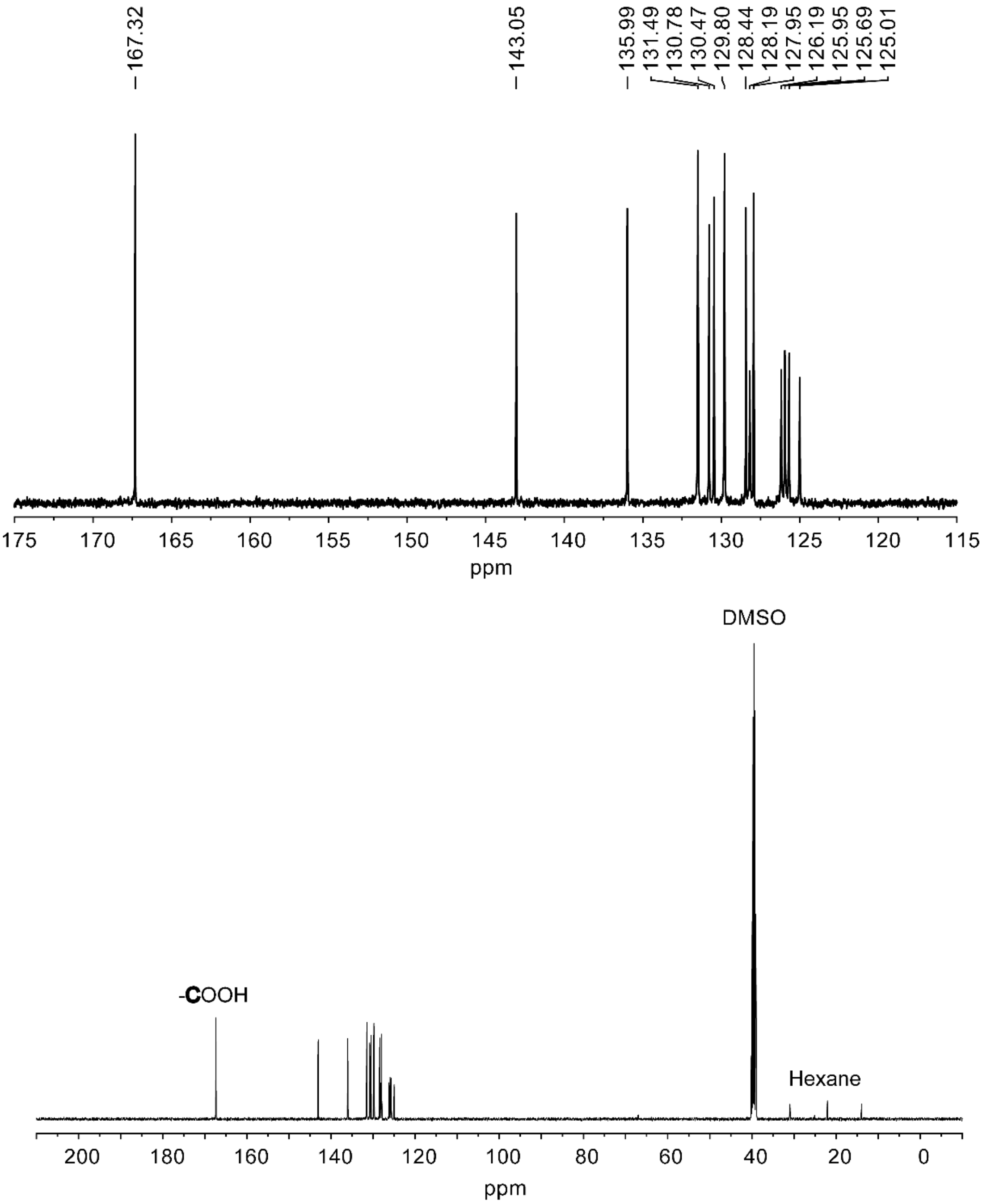

Supplementary Figure 5 | Top: enlargement of the aromatic region of ^{13}C liquid NMR of 5,12-bis(4-carboxyphenyl) tetracene (**4**) in the range between 115 ppm and 175 ppm. Bottom: ^{13}C liquid NMR of 5,12-bis(4-carboxyphenyl) tetracene(**4**) in the range between -10 ppm and 210 ppm. The spectrum was collected on a AVANCE NEO Bruker instrument (400 MHz) at 298.1 K in DMSO_d_6.

2.3 Infrared spectra

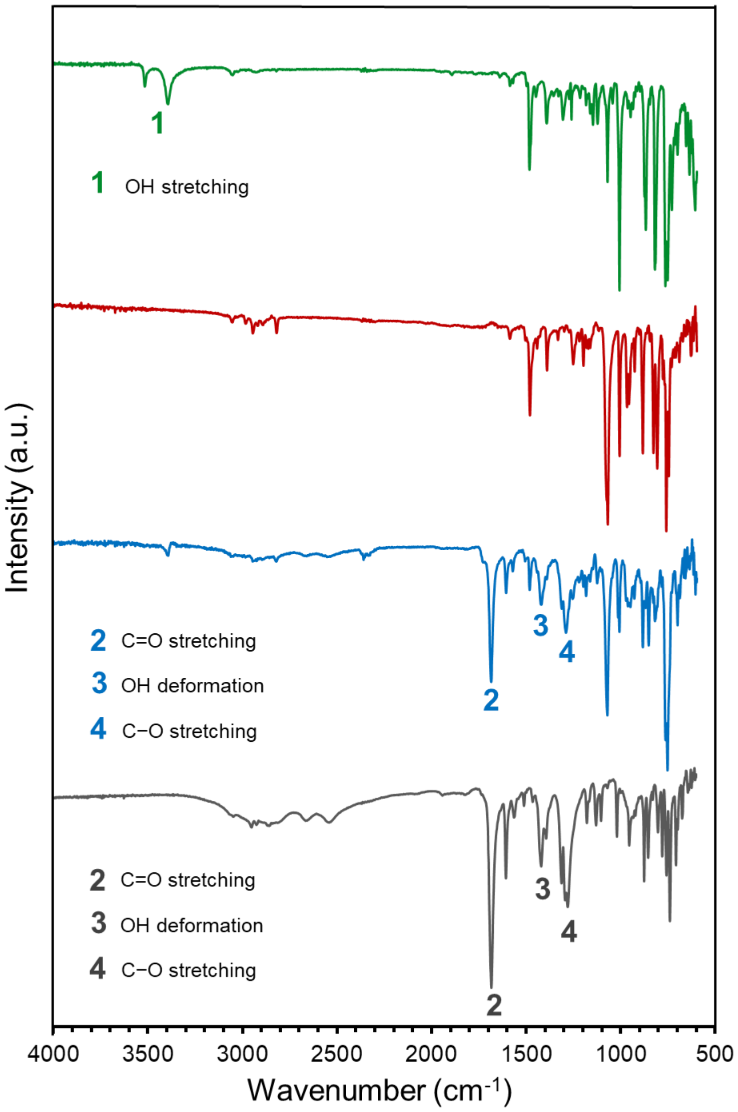

Supplementary Figure 6 | Infrared spectra of 5,12-bis(4-bromophenyl)-5,12-dihydronaphtacene (**1**) (Top, dark green), 5,12-bis(4-bromophenyl)-5,12-diomethoxy-naphtacene (**2**) (red), 5,12-bis(4-carboxyphenyl)-5,12-dimethoxynaphtacene (**3**) (light blue) and 5,12-bis(4-carboxyphenyl) tetracene (**4**) (Bottom, grey). [1]

2.4 Photophysical properties of ligands

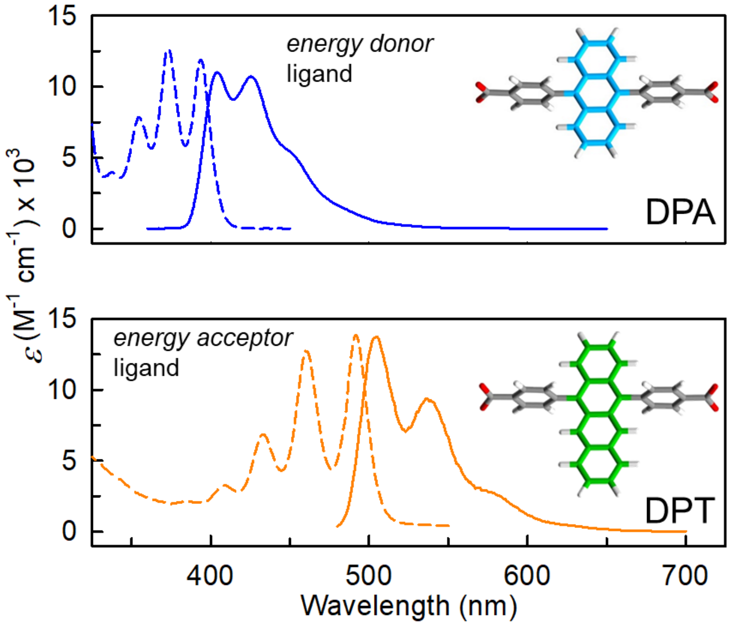

Supplementary Figure 7 | Absorption and photoluminescence (PL) spectra of DPA (top) and DPT (bottom) chromophores. Their molecular structures are reported as insets.

Figure S7 shows the molecular structures and optical/photoluminescence properties of the selected conjugated ligands. The DPA is a fluorescence standard with nearly unitary QY.[6] Its dicarboxylated derivative maintains its photoluminescence properties and it can be combined with zirconium oxy-hydroxy clusters to fabricate stable MOF nanocrystals. The DPT shows a QY = 0.80 (Methods) and has been chosen as energy acceptor because its absorption/emission properties are complementary to the ones of DPA. The photoluminescence spectrum of this latter, peaked at 430 nm, is strongly resonant with the DPT first ground state absorption peaked at 490 nm. Therefore, the DPT is an ideal energy acceptor system for non-radiative energy transfer from DPA.[7] The DPT photoluminescence in the visible spectral range, peaked at 515 nm, is completely off resonance with respect the DPA absorption, thus avoiding the possibility of emission re-absorption.

3. *Homo*-ligand Zr-MOFs

3.1 Synthetic procedure

Procedure for MOF synthesis (Zr-DPT). The synthetic procedure was optimized to generate highly crystalline MOFs. Zirconium chloride (58.2 mg; 0.25 mmol) and 5,12-bis(4-carboxyphenyl) tetracene (117.1 mg; 0.25 mmol) were dispersed in dry DMF (25 mL) in a 100 mL glass vial and acetic acid was added (1.72 mL; 30 mmol). A stream of dry nitrogen was bubbled through the reaction mixture for 10 minutes to remove air from the vials. The vial was closed under inert atmosphere and the mixture was sonicated for 1 minutes at room temperature and then heated at 120 °C for 22 hours. The closed vial was removed from the oven and cooled down to room temperature and the powdery material was filtered on 0.1 µm PTFE membrane, repeatedly washed with a total amount of 50 mL DMF and 50 mL tetrahydrofuran. The sample was activated at 130°C overnight under high vacuum (10^{-2} torr) before further measurements.

Procedure for MOF synthesis (Zr-DPA). Zr-DPA nanocrystals were prepared according to literature.[1] Briefly, Zirconium chloride (116.5 mg; 0.50 mmol) and 9,10- bis(4-carboxyphenyl)anthracene (209 mg; 0.50 mmol) were dispersed in a mixture of dry DMF (50 mL) and deionized water (50 µL) in a 100 mL glass vial and acetic acid was added (1.43 mL). The mixture was sonicated for 1 minutes at room temperature and then heated at 120 °C for 22 hours. The vial was cooled down to room temperature and the slightly yellowish solid was filtered on 0.1 µm PTFE membrane, washed with DMF and tetrahydrofuran and dried. The sample was activated at 130 °C overnight under high vacuum before further measurements.

3.2 Infrared spectroscopy

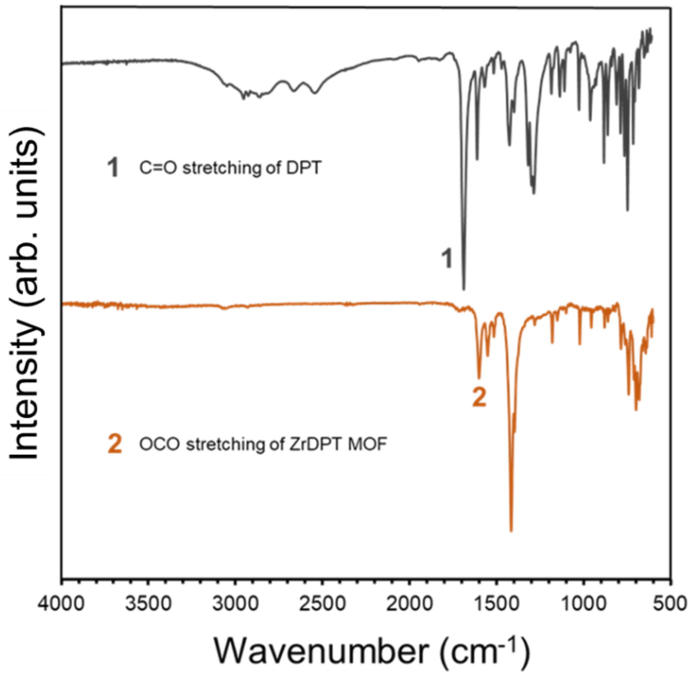

Supplementary Figure 8 | Infrared spectra of DPT chromophore (Top, grey), and Zr-DPT (Bottom, orange). The OCO stretching showed a shift to lower wavenumber (from 1683 cm^{-1} to 1600 cm^{-1}) due to coordination with the zirconium ions-based cluster.

3.3 ^{13}C CP MAS NMR and ^{1}H MAS solid state NMR of Zr-DPT

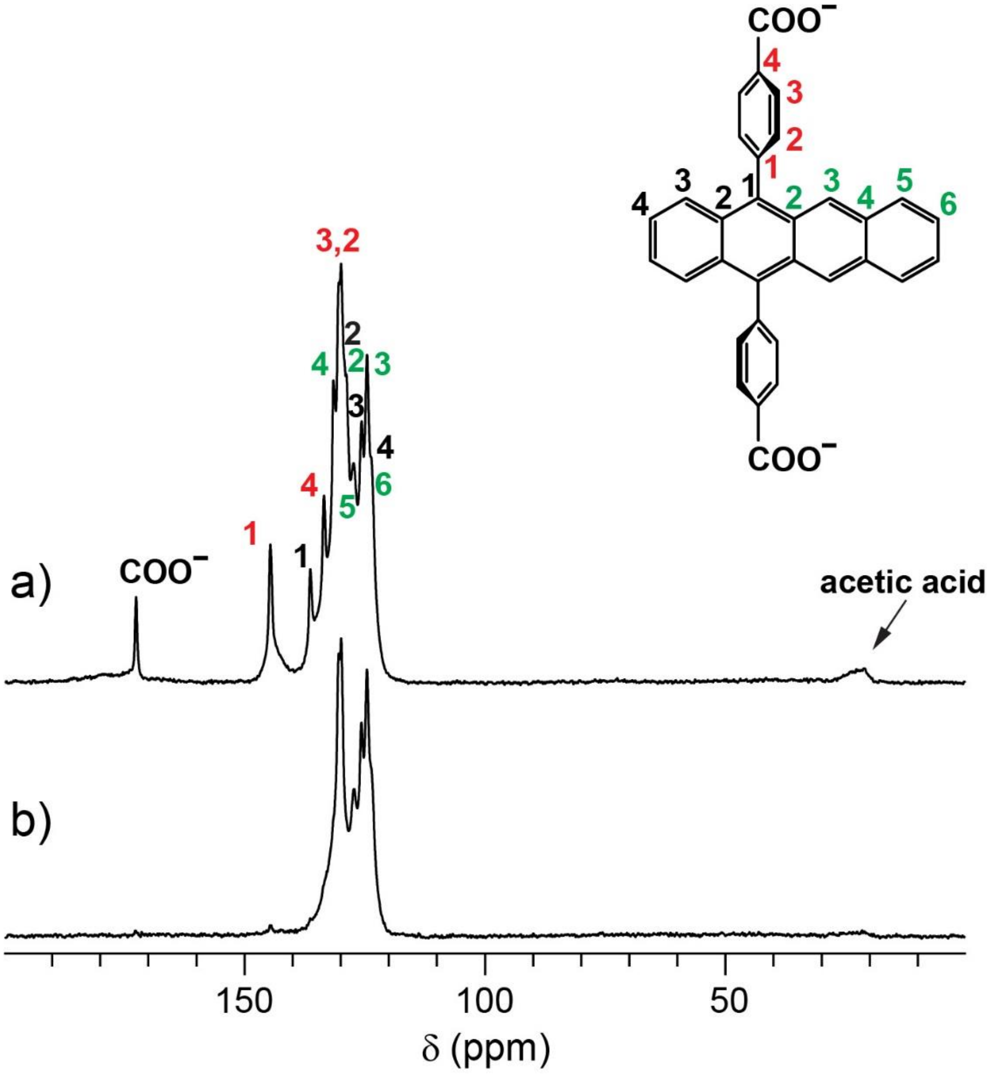

Supplementary Figure 9 | ^{13}C CP MAS spectra of Zr-DPT collected at 75 MHz, 298 K at a spinning speed of 12.5 kHz and contact time of a) 2 ms and b) 0.05 ms.

Supplementary Table 1 | ^{13}C and ^{1}H chemical shifts of Zr-DPT from solid state NMR spectra collected at 7.04 T.

**Zr-DPT**	**Assignment**	**δ (ppm)**
	**C_1**	136.44
	**C_2, C_2**	129.30; 128.92
	**C_3**	125.73
	**C_4, C_6**	123.77
	**C_1**	144.74

<table>
<tr><td rowspan="7">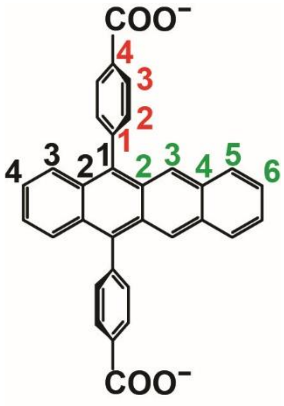</td><td>C_2, C_3</td><td>130.47; 130.03</td></tr>
<tr><td>C_4</td><td>133.57</td></tr>
<tr><td>C_3</td><td>124.61</td></tr>
<tr><td>C_4</td><td>131.62</td></tr>
<tr><td>C_5</td><td>127.41</td></tr>
<tr><td>COO^-</td><td>172.74</td></tr>
<tr><td>CH_3 (acetic acid)</td><td>20.89</td></tr>
<tr><td rowspan="4">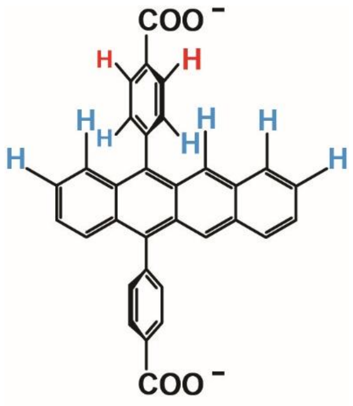</td><td colspan="2"></td></tr>
<tr><td>H</td><td>8.7</td></tr>
<tr><td>H</td><td>6.9</td></tr>
<tr><td colspan="2"></td></tr>
</table>

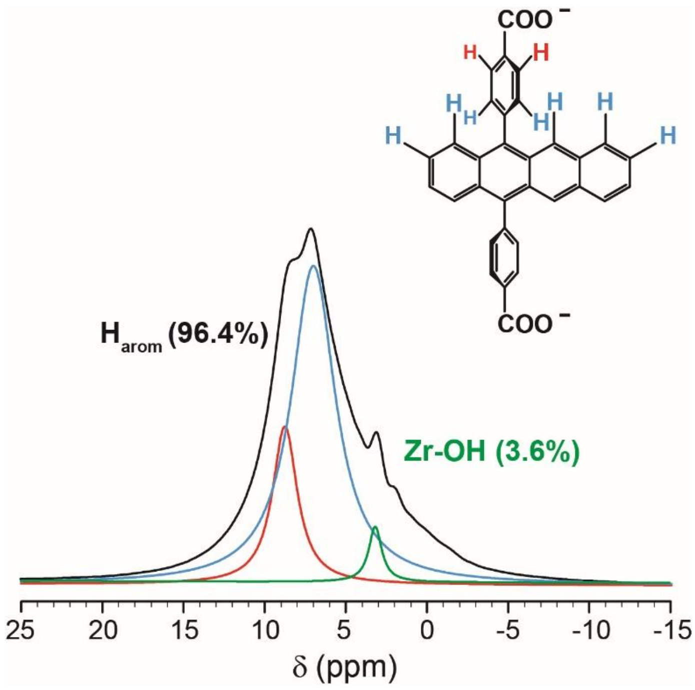

Supplementary Figure 10 | ^{1}H MAS spectrum of Zr-DPT collected at 300 MHz, 298 K at a spinning speed of 12.5 kHz and recycle delay of 20 s. It is possible to identify separately the signals of both aromatic and OH groups. The quantitative analysis is in agreement with the hydrogen content about the Zr cluster.

3.4 SEM images and particle size distribution

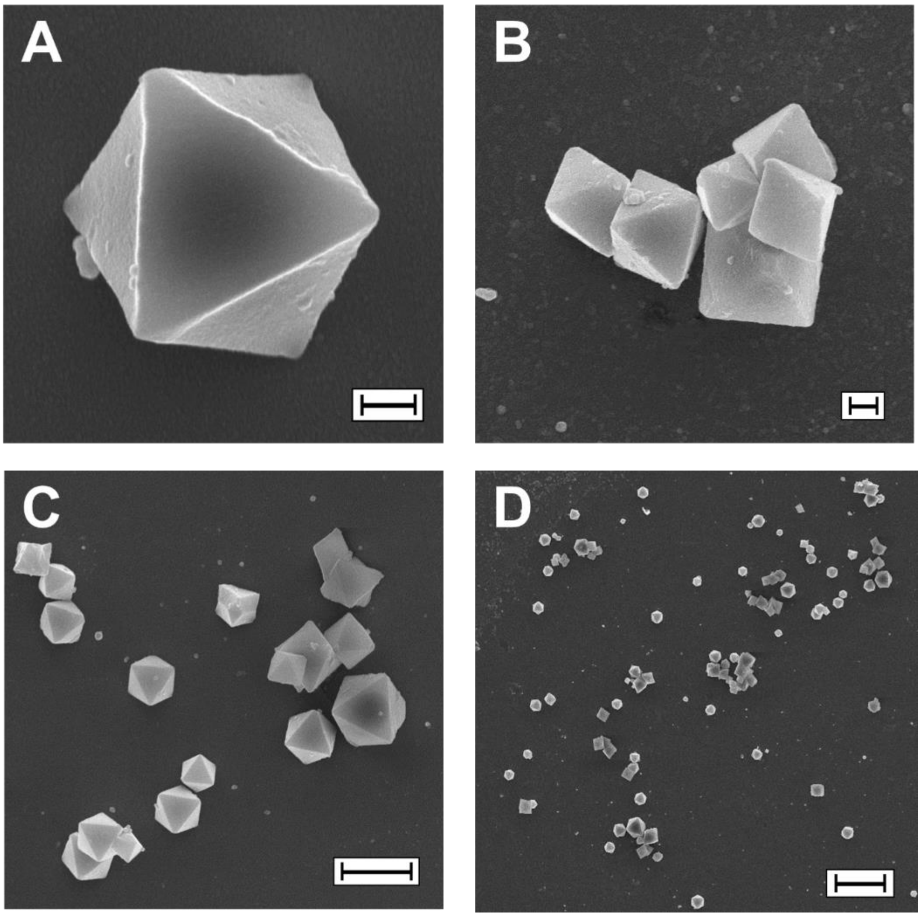

Supplementary Figure 11 | SEM images of Zr-DPT at different magnification. The scale bar is 200 nm (A, B), 1 µm (C) and 3 µm (D), respectively.

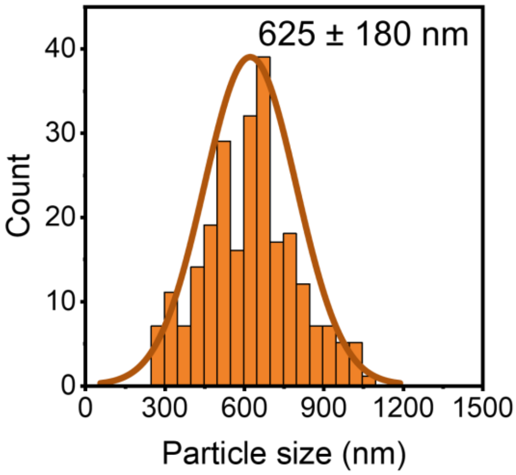

Supplementary Figure 12 | Particle size distribution measured from SEM images of Zr-DPT (orange).

3.5 Powder x-ray diffraction and crystal structure

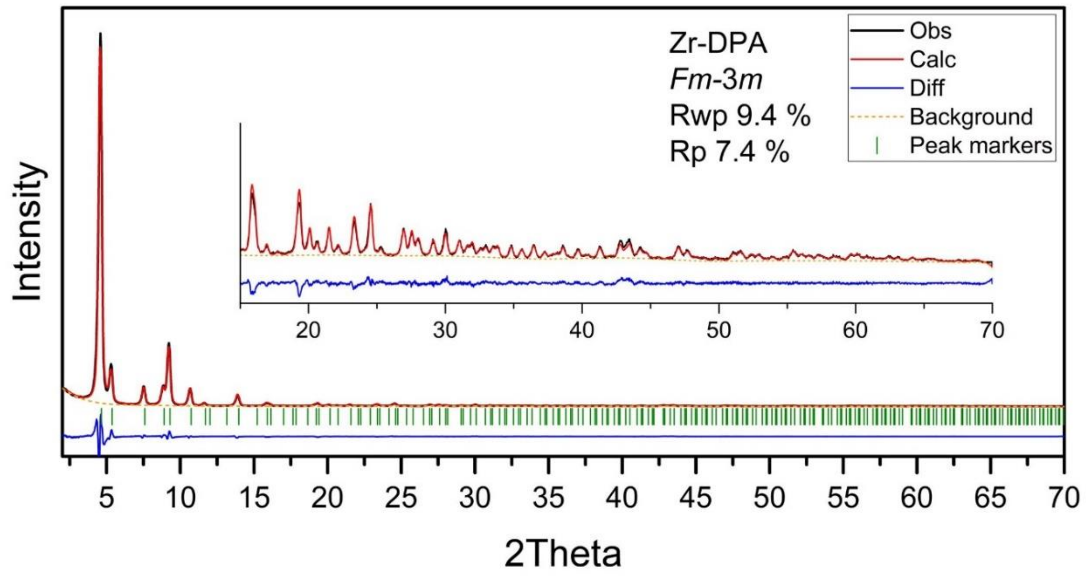

Supplementary Figure 13 | Rietveld fits of X-ray data for Zr-DPA using the TOPAS-Academic-64 V6 software package [S1]. The HKL indices are indicated as green markers and the difference plot are shown below. The black and red represent the observed and calculated traces respectively while the dotted yellow line represent the background plot.

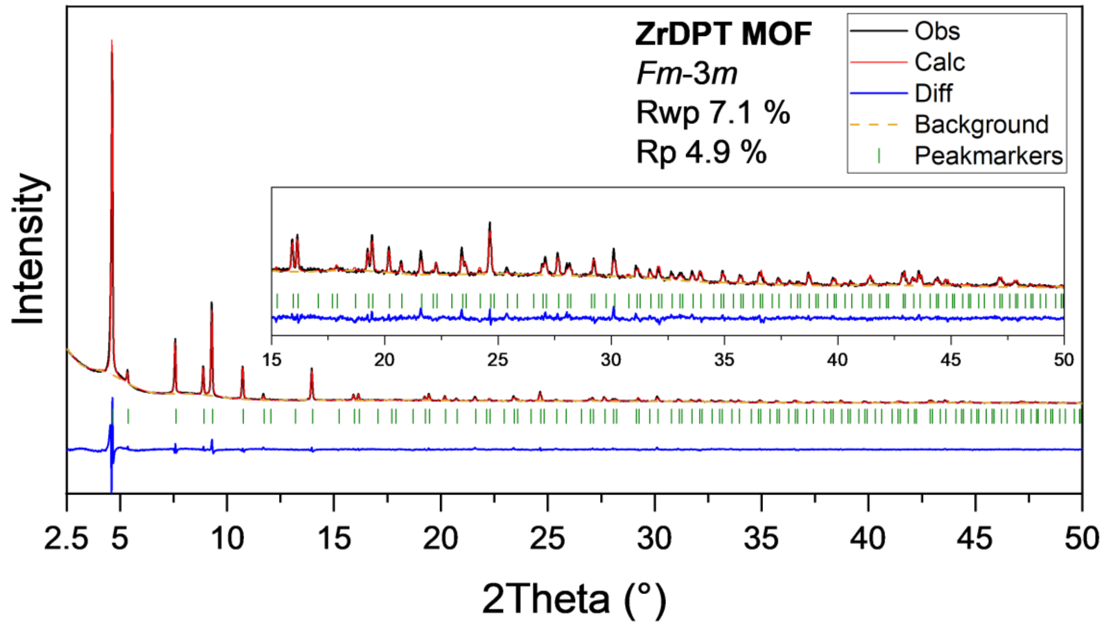

Supplementary Figure 14 | Rietveld fits of X-ray data for Zr-DPT using the TOPAS-Academic64 V6 software package.[2] [3]The HKL indices are indicated as green markers and the difference plot is shown below. The black and red profiles represent the observed and calculated traces, respectively, while the dotted yellow line represents the background plot.

Supplementary Table 2 | Crystallographic data for the PXRD Rietveld refinement of **Zr-DPT** and **Zr-DPA** MOFs.

**Identification code**	**Zr-DPT**	**Zr-DPA**[1]
Empirical formula	$C_{3.5625}H_{2.0625}O_{0.67}Zr_{0.125}$	$C_{3.5}H_{2}O_{0.67}Zr_{0.125}$
Formula weight	66.93	66.12
Temperature/K	RT	RT
Crystal system	cubic	cubic
Space group	*Fm*-3*m*	*Fm*-3*m*
a=b=c/Å	32.8447(17)	32.9126(14)
Volume/Å^3	35432(5)	35652(5)
Z	192	192
ρ_{calc}g/cm^3	0.64902(1)	0.59131(8)
Rp	4.9 %	7.3 %
Rwp	7.1 %	9.4 %

Powder X-ray diffraction (PXRD) patterns are consistent with highly ordered crystalline porous materials and the cubic crystal structures (space group *Fm-3m*) were refined by the Rietveld method combined with molecular mechanics and plane-wave DFT calculations. The Zr-based nodes ($Zr_6(\mu_3\text{-}O)_4(\mu_3\text{-}OH)_4(CO_2)_{12}$ clusters) have 12-coordinated DPT ligands which connect the nodes in the 3D framework while the DPT units are disordered over four positions with equal occupancy (0.25). The crystal structure of the MOFs contains interconnected octahedral cavities, connected through the tetrahedral spaces (the volumes were calculated by a sphere of 1.82 Å radius and a grid spacing of 0.15 Å.). The tetrahedral spaces do not form clear cavities due to the crowded arrangement of tetracene moieties occupying that space.

The **DPT** phenyl rings are disordered over two positions while the tetracene moieties are disordered over 4 positions due to their off-centered connectivity to the phenyl rings. The disordered position of tetracene moieties shows an angle of 37.27° with respect to one another and are related to each other by mirror symmetry.

The **DPA** units coordinated to Zr-based nodes are disordered over three positions of which one comprises an anthracene moiety that lies within a mirror plane. The remaining two positions exhibit the anthracene moieties tilted by ±18.6° and are related to each other by the mirror symmetry. The shortest center-to-center distance between the anthracene moieties can be measured from the crystal structure as 11.7 Å.

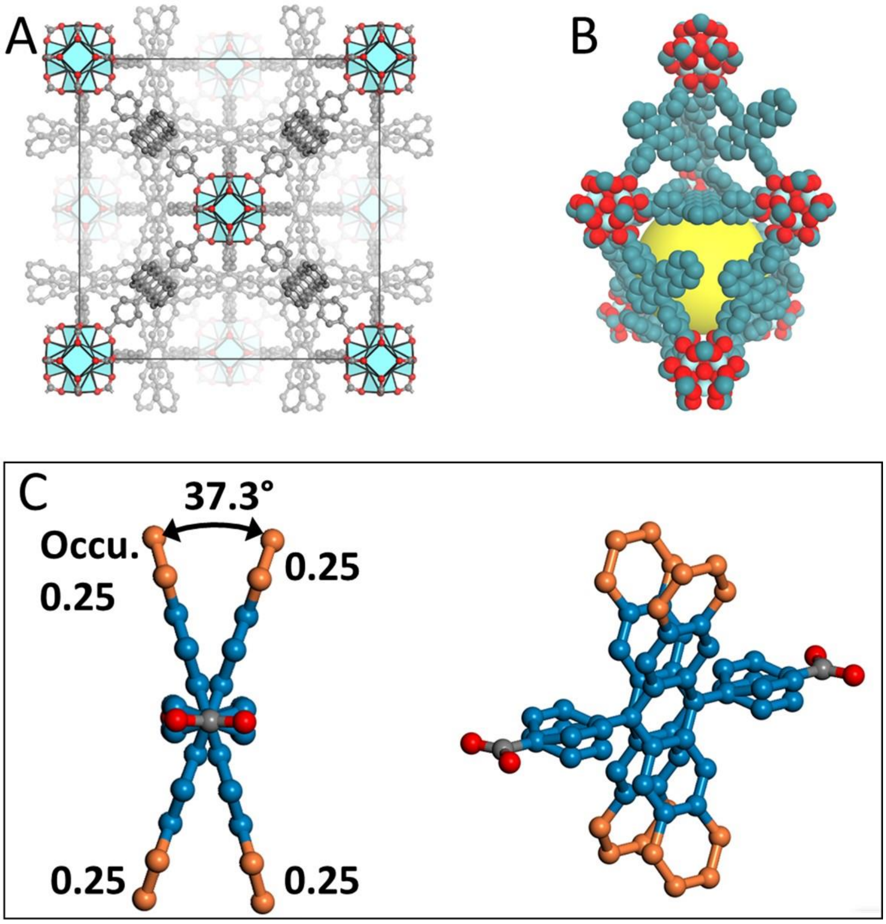

Supplementary Figure 15 | A) Crystal structure for the Zr-DPT, *Fm-3m*, disordered model. The ligand is disordered over four positions of which two for the phenyl rings are related by symmetry, reducing it to two unique orientations. B) Space filling representation of the tetrahedral and octahedral spaces of Zr-DPT. The yellow sphere indicates the cavity of the octahedral space while the tetrahedral space is occupied by a crowded arrangement of tetracene moieties. C) The disorder of the DPT ligand with the four carbon atoms of the tetracene moiety, disordered over four positions (0.25 occupancy), shown in orange. Hydrogen atoms have been omitted for clarity.

Supplementary Table 3 | Nominal fraction of DPT molecules with respect to DPA and number of ligands per unit cell.

Sample (nominal molar fraction%)	Number of DPT ligands per unit cell
**Zr-DPA:DPT-0.1%**	0.024
**Zr-DPA:DPT-0.5%**	0.12
**Zr-DPA:DPT-1%[a]**	0.24
**Zr-DPA:DPT-8%[a]**	1.92
**Zr-DPT**	24

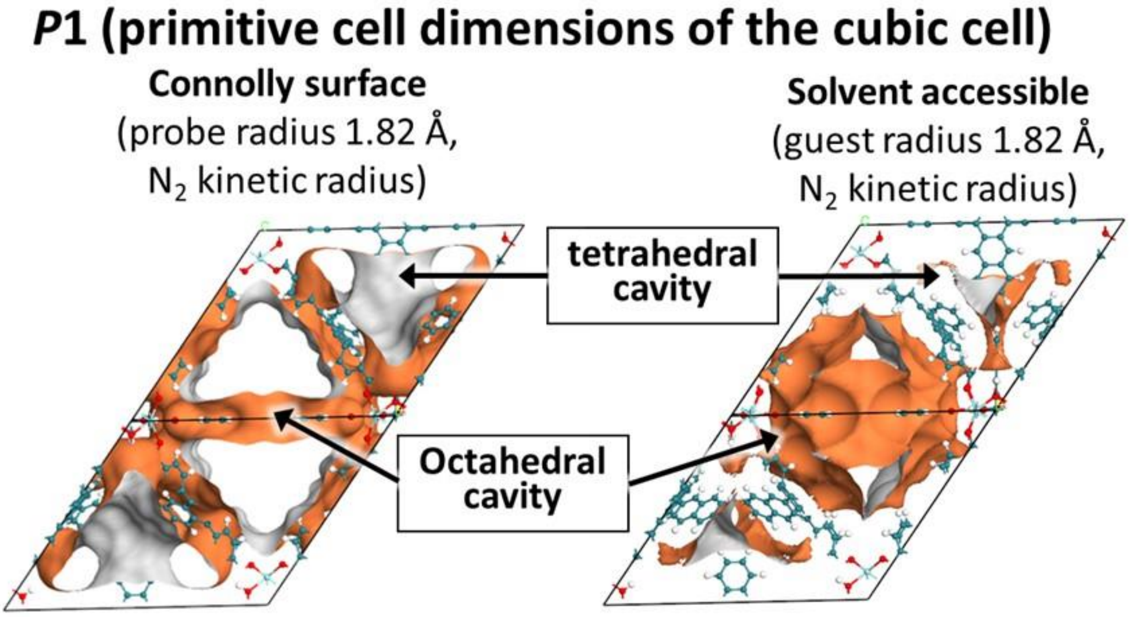

Supplementary Figure 16 | Crystal structure for the Zr-DPT, *P*1, ordered model. This model is a combination of the two unique DPT phenyl orientations and the four unique tetracene orientations while maintaining their respective occupancy ratios. The contact surface and solvent accessible volumes were calculated using Materials Studio software with a probe radius of 1.82 Å (shown in orange, N_2 kinetic diameter is 3.64 Å and solvent radius of 1.82 Å) and a grid spacing of 0.15 Å. The calculated pore volumes are 0.421 cm³ g^{-1} (solvent accessible volume) and 1.03 cm³ g^{-1} (contact surface volume). Hydrogen atoms have been omitted for clarity.

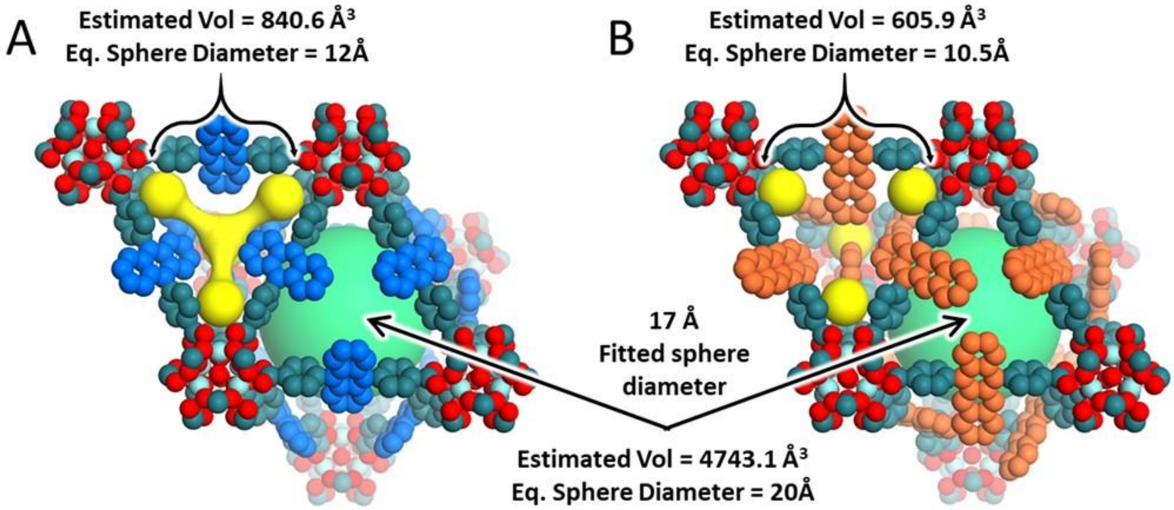

Supplementary Figure 17 | The space filling representation (0.6 vdW radius) of the tetrahedral and octahedral spaces of A) Zr-DPA and B) Zr-DPT with the DPA moieties shown in blue and the DPT units shown in orange. The yellow volumes indicate the illustrated cavities of the tetrahedral spaces while the green sphere occupies the octahedral space. For Zr-DPT the central space is occupied by a crowded arrangement of tetracene moieties reducing the pore volume with respect to the tetrahedral pore of Zr-DPA. From the estimated volumes for the octahedral and tetrahedral spaces, we can estimate the diameter of the spheres as indicated. These results agree with the pore size distribution data. All volumes were calculated using Materials Studio with a probe radius of 1.82 Å and hydrogen atoms have been omitted for clarity.

Supplementary Table 4 | The pore volumes and surface areas, determine using a probe radius of 1.82 Å, for **Zr-DPT** and **Zr-DPA**. The sphere diameters were calculated from spheres with volumes equivalent to those of V_{oct} and V_{tet}.

**Identification code**	**Zr-DPT**	**Zr-DPA**[1]
Total pore volume (Å^3)	23819.92	25697.32
Total pore volume (cm^3·g^{-1}) Connoly surface	1.03	1.22
Surface area (cm^2·g^{-1})	3800	3983
V(octahedral) (Å^3)	4743.1	4743.1
Sphere Diameter (Eq. to V_{oct})	20 Å	20 Å
V(tetrahedral) (Å^3)	605.9	840.6
Sphere Diameter (Eq. to V_{tet})	10.5 Å	12 Å

3.6 Nitrogen adsorption isotherm at 77 K and sorption properties

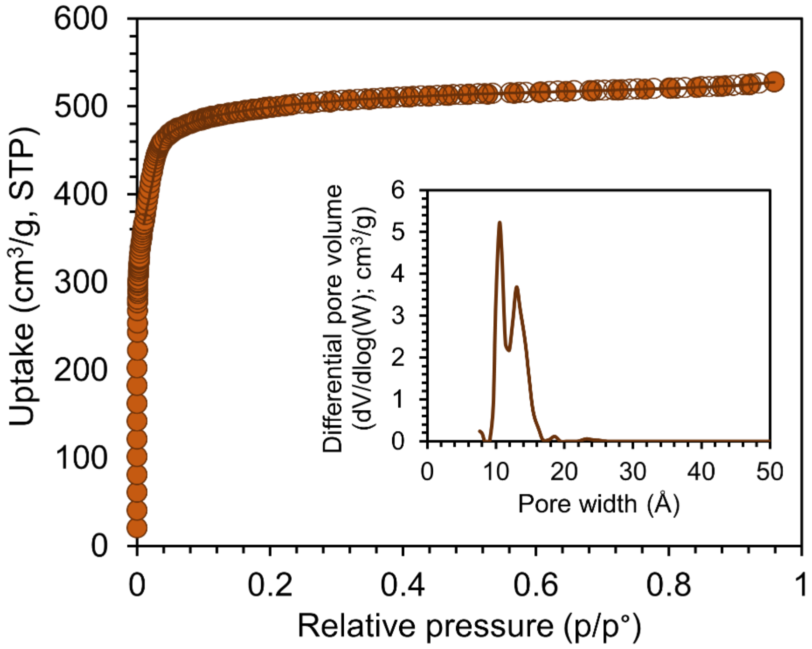

Supplementary Figure 18 | Nitrogen adsorption isotherm of Zr-DPT collected at 77 K. Inset: Pore size distribution calculated according to NLDFT theory (N_2 on carbon slit pore method). The pore size distribution featured 2 distinct micropores of diameter size of 10.8 Å and 14.5 Å.

4. *Hetero*-ligand MOF: Zr-DPT:DPA-x%

4.1 Synthetic procedure for Zr-DPT:DPA-x%

General procedure for mixed-ligand MOF nanocrystals (Zr-DPT:DPA-x%). Zirconium chloride (116.5 mg; 0.50 mmol), 9,10-bis(4-carboxyphenyl) anthracene and 5,12- bis(4-carboxyphenyl) tetracene (for the molar fraction of the two ligands, see Table S1) were dispersed in a mixture of dry DMF (50 mL) and deionized water (25 µL) in a 100 mL glass vial and a proper amount of acetic acid was added (see Table S1). A stream of dry nitrogen was bubbled through the reaction mixture for 10 minutes to remove air from the vials. The vial was closed under inert atmosphere and the mixture was sonicated for 1 minutes at room temperature and then heated at 120 °C for 22 hours in an oven. The closed vial was cooled down to room temperature and the powdery material was filtered on 0.1 µm PTFE membrane, repeatedly washed with a total amount of 50 mL DMF and 50 mL tetrahydrofuran. The sample was activated at 130°C overnight under high vacuum before further measurements.

Supplementary Table 5 | Reagents and solvents employed in MOF synthesis.

Sample (nominal molar fraction%)	$ZrCl_4$ (mmol)	$DPA(COOH)_2$ (mmol)	$DPT(COOH)_2$ (mmol)	Acetic acid (mmol)
**Zr-DPA:DPT-0.1%**	0.5	0.498	0.00049	25
**Zr-DPA:DPT-0.5%**	0.5	0.497	0.0025	25
**Zr-DPA:DPT-1%[a]**	0.5	0.494	0.0050	25
**Zr-DPA:DPT-8%[a]**	0.5	0.458	0.040	25
**Zr-DPT**	0.25	-	0.25	30

a) The content of DPT moieties in Zr-DPT:DPA-8% was confirmed by ^{1}H NMR on the digested sample.

4.2 Digital image of MOF samples

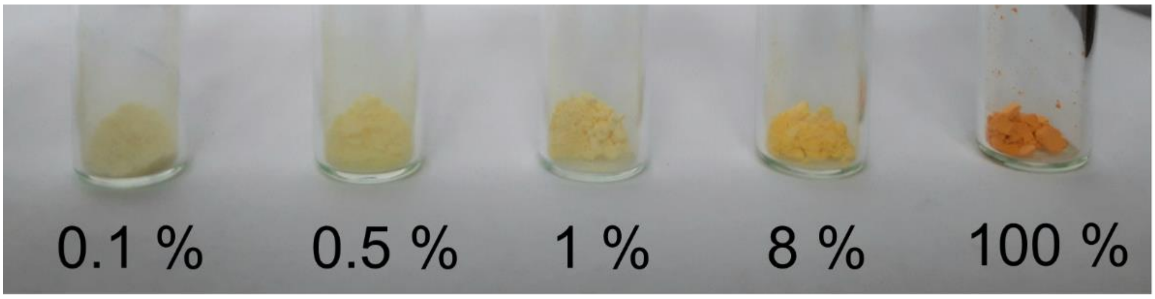

Supplementary Figure 19 | Digital image of zirconium-based MOFs (Zr-DPT:DPA-x%). The molar percentage of diphenyl-tetracenedicarboxylate moieties inside each sample is reported.

4.3 ^{1}H NMR of digested samples

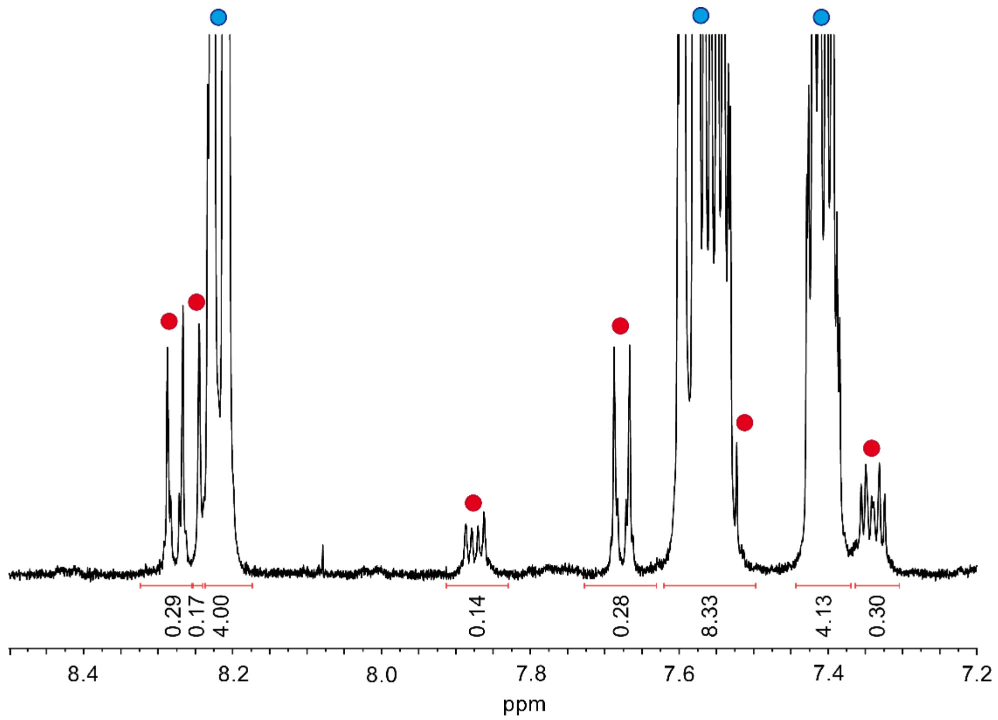

Supplementary Figure 20 | ^{1}H-NMR of digested Zr-DPT:DPA-8%. The peaks associated with the diphenyl-tetracene dicarboxylate and diphenyl-anthracene dicarboxylate moieties are highlighted by red and blue dots, respectively. Integration of the peaks allows to calculate the relative percentage of DPT and DPA in the MOF structure. The spectrum was collected on a AVANCE NEO Bruker instrument (400 MHz) at 298.1 K in DMSO_d_6 (0.8 mL)/Trifluoroacetic acid (0.15 mL) mixture.

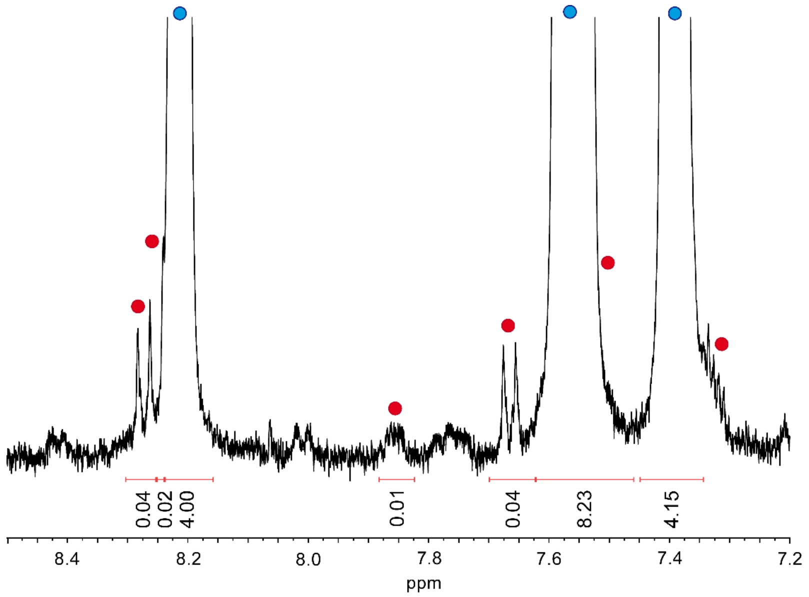

Supplementary Figure 21 | ^{1}H-NMR of digested Zr-DPT-DPA-1%. The peaks associated with the diphenyl-tetracene dicarboxylate and diphenyl-antracene dicarboxylate moieties are highlighted by red and blue dots, respectively. Integration of the peaks allows to calculate the relative percentage of DPT and DPA inside the MOF structure. . The spectrum was collected on a AVANCE NEO Bruker instrument (400 MHz) at 298.1 K in DMSO_d_6 (0.8 mL)/Trifluoroacetic acid (0.15 mL) mixture.

4.4 Infrared spectroscopy of Zr-DPT:DPA-X%

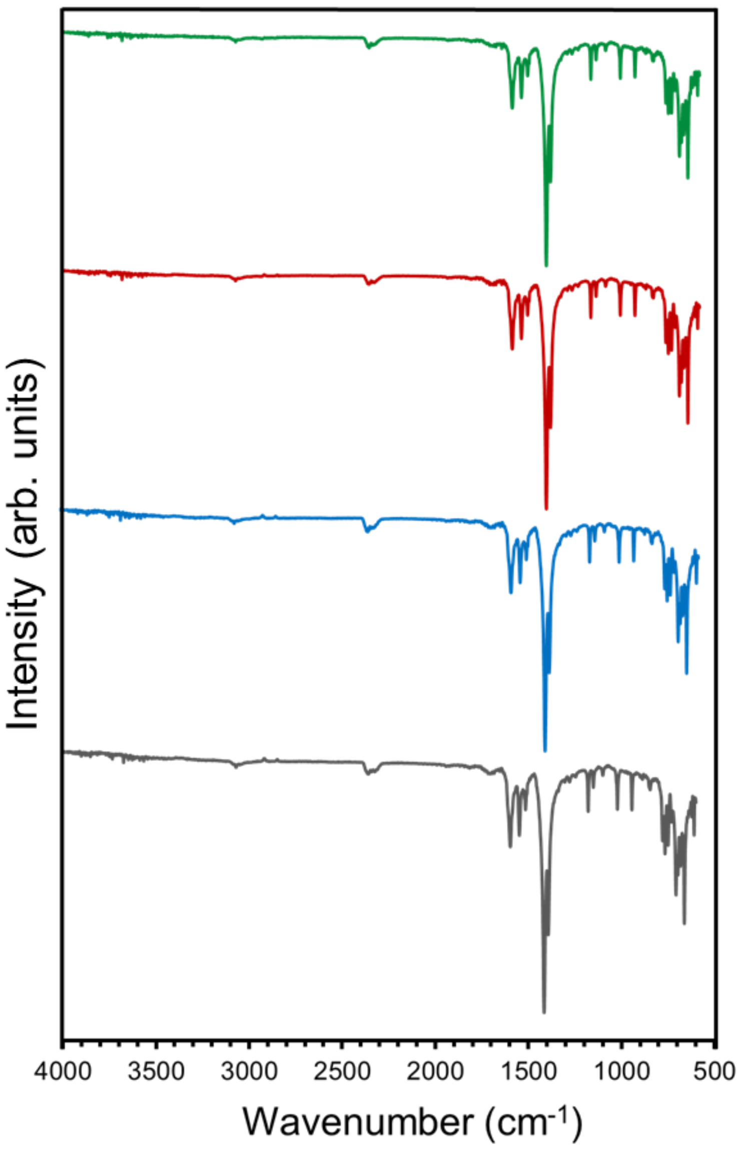

Supplementary Figure 22 | Infrared spectra of Zr-DPT:DPA-8% (dark green), Zr-DPT:DPA-1% (red), Zr-DPT:DPA-0.5% (light blue) and Zr-DPT:DPA-0.1% (grey).

4.5 ^{13}C CP MAS, ^{1}H MAS and 2D ^{1}H-^{13}C PMLG HETCOR solid state NMR

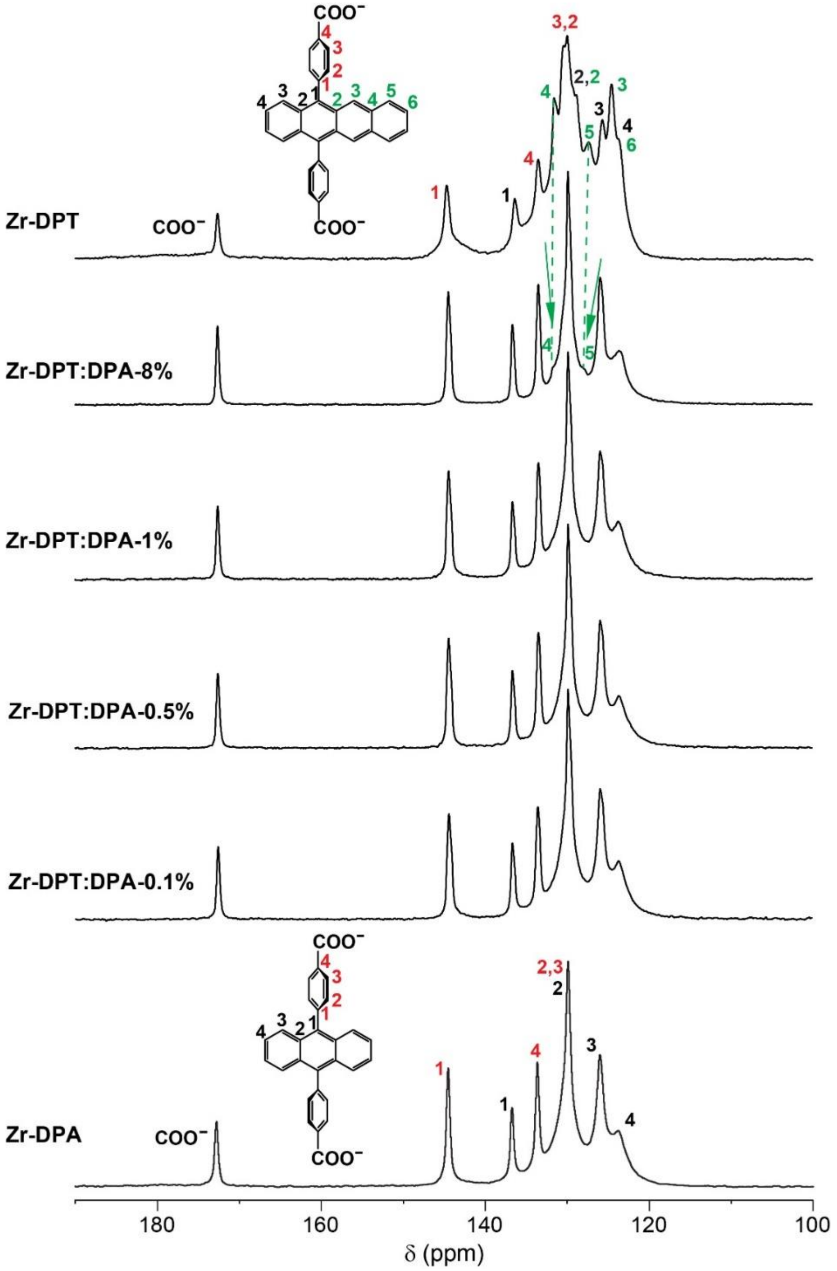

Supplementary Figure 23 | ^{13}C CP MAS spectra of Zr-DPT, Zr-DPT:DPA-8%, Zr-DPT:DPA-1%, Zr-DPT:DPA-0.5%, Zr-DPT:DPA-0.1% and Zr-DPA collected at 75 MHz, 298 K at a spinning speed of 12.5 kHz and contact time of 2 ms.

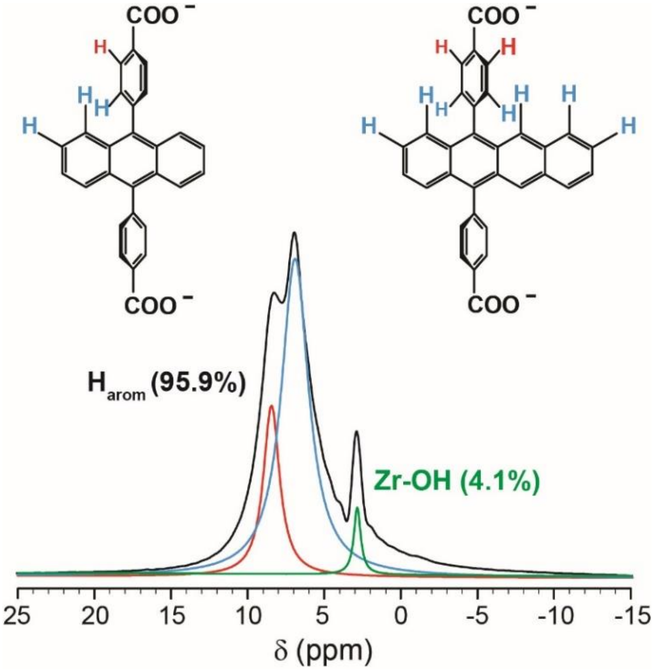

Supplementary Figure 24. ^{1}H MAS spectrum of Zr-DPT:DPA-8% collected at 300 MHz, 298 K at a spinning speed of 12.5 kHz and recycle delay of 20 s. It is possible to identify separately the signals of both aromatic and OH groups. The quantitative analysis agrees with the hydrogen content about the Zr cluster.

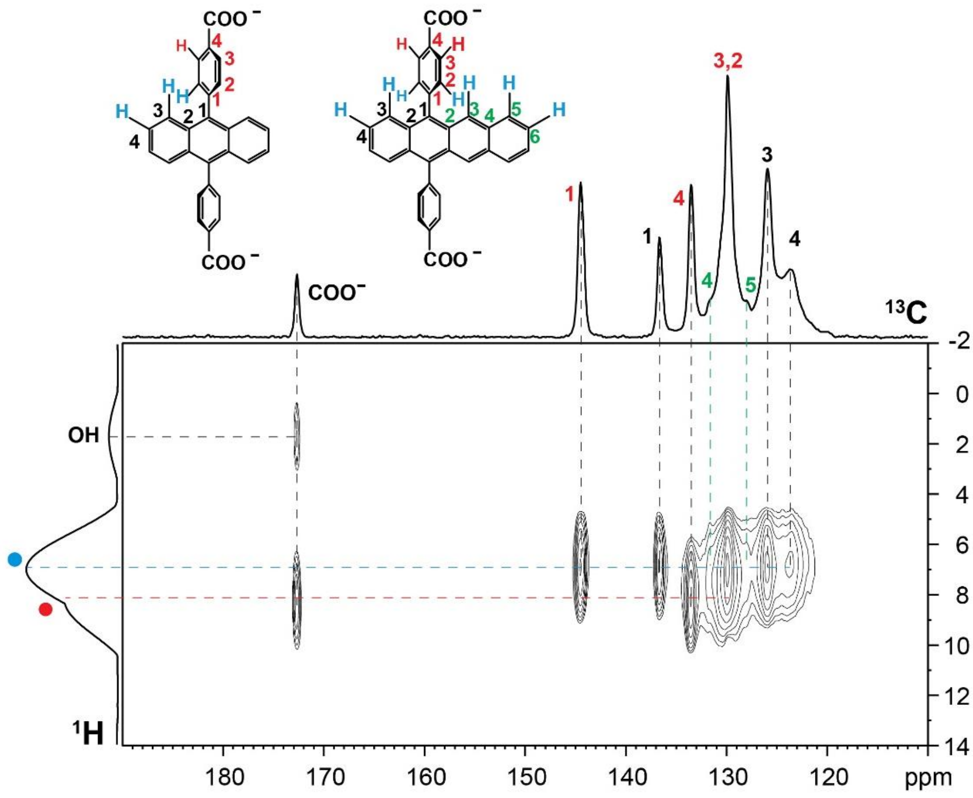

Supplementary Figure 25 | 2D ^{1}H-^{13}C PMLG HETCOR NMR spectrum of Zr-DPT:DPA 8% collected at 75 MHz, 298 K, at a spinning speed of 12.5 kHz and contact time of 2 ms.

4.6 Thermogravimetric analysis (TGA)

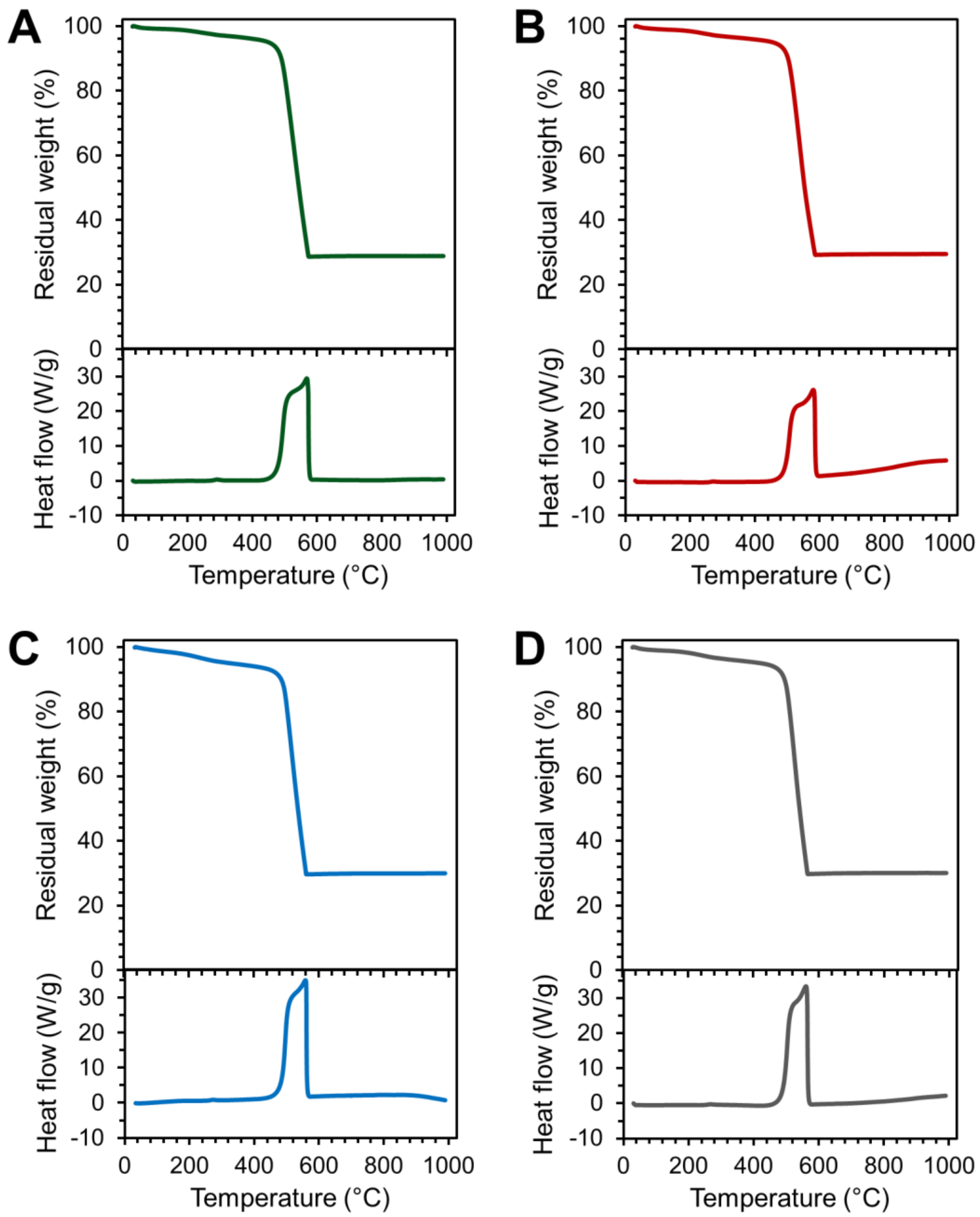

Supplementary Figure 26 | Thermogravimetric analysis and heat flow measurements during the thermal ramps of sample Zr-DPT:DPA-8% (A, green), Zr-DPT:DPA-1% (B, red), Zr-DPT:DPA-0.5% (C. light blue) and Zr-DPT:DPA-0.1% (D, grey) collected under oxydative conditions (dry air flow at 50 mL/min). All samples show a weight loss between 200°C and 300°C associated with water desorption and rearrangement of the zirconium. All samples display high thermal stability up to 450°C.

4.7 SEM images and particle size distribution

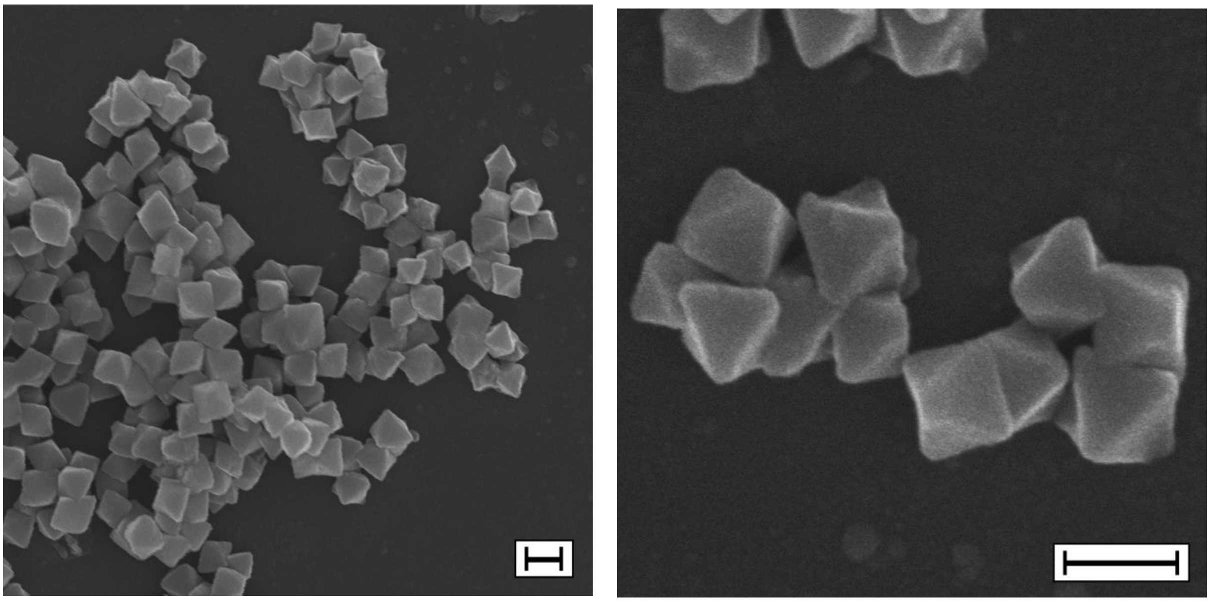

Supplementary Figure 27 | SEM images of Zr-DPT:DPA-8% at different magnification. The scale bar is 200 nm.

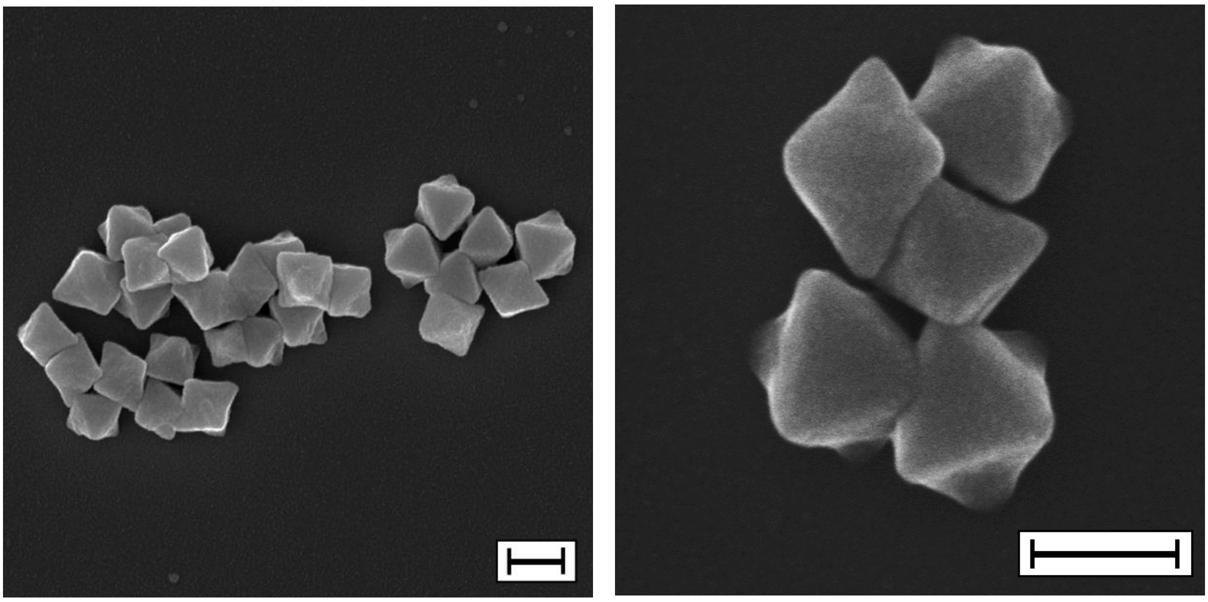

Supplementary Figure 28 | SEM images of Zr-DPT:DPA-1% at different magnification. The scale bar is 200 nm.

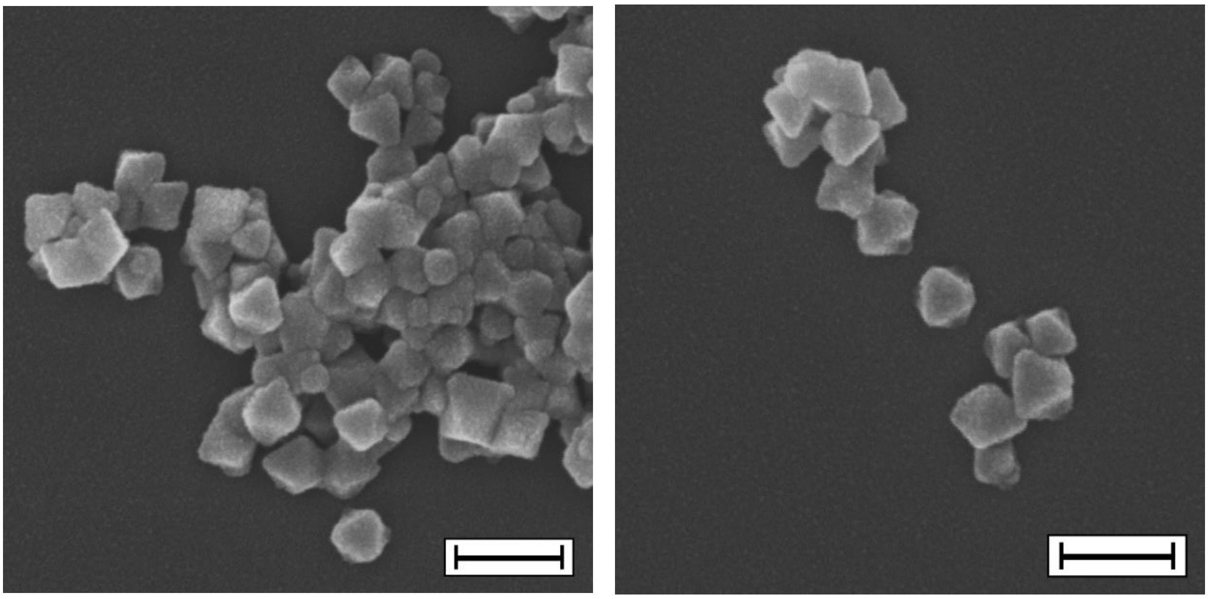

Supplementary Figure 29 | SEM images of Zr-DPT:DPA-0.5% at different magnification. The scale bar is 200 nm.

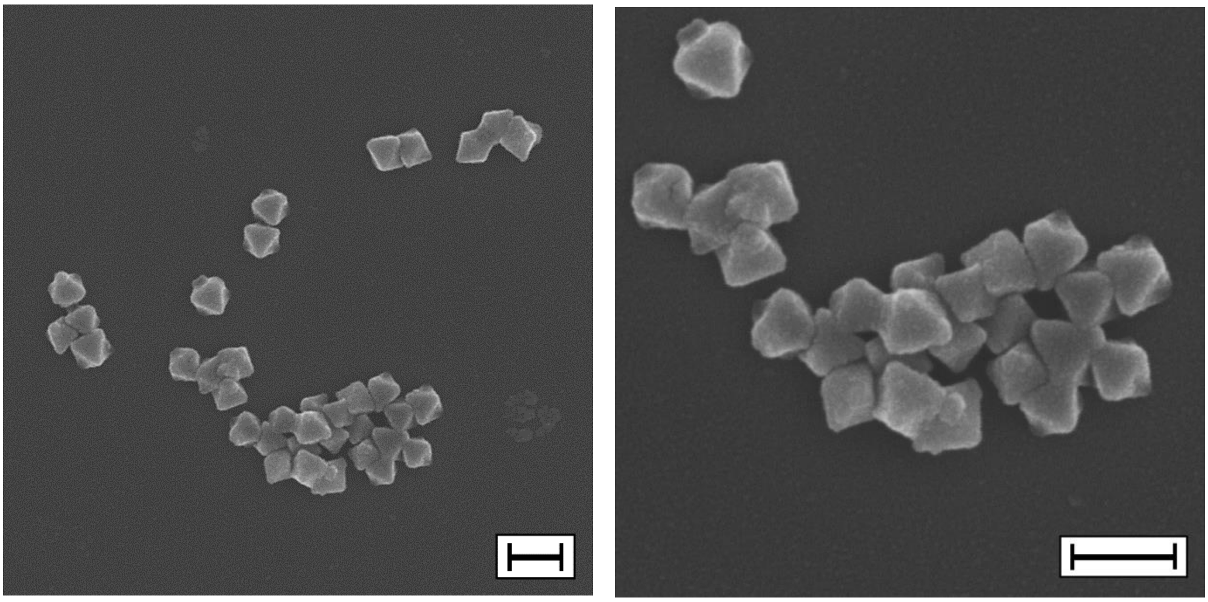

Supplementary Figure 30 | SEM images of Zr-DPT:DPA-0.1% at different magnification. The scale bar is 200 nm.

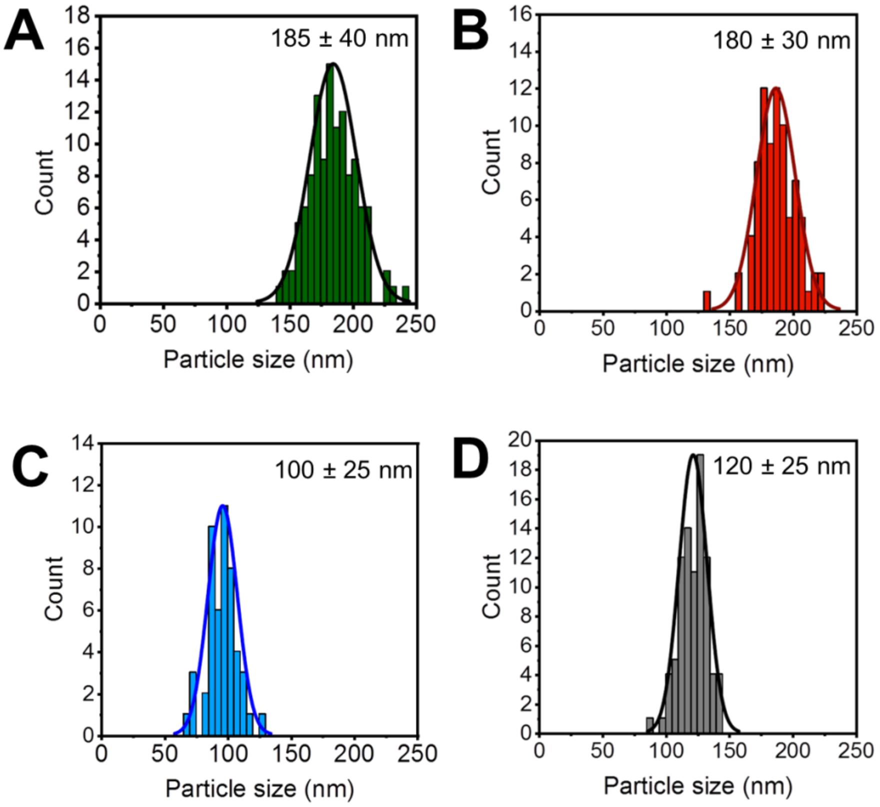

Supplementary Figure 31 | Particle size distribution measured from SEM images of Zr-DPT:DPA-8% (A, green), of Zr-DPT:DPA-1% (B, red), of Zr-DPT:DPA-0.5% (C, blue) and of Zr-DPT:DPA-0.1% (D, grey).

4.8 Powder x-ray diffraction and crystal structure of Zr-DPT:DPA-x%

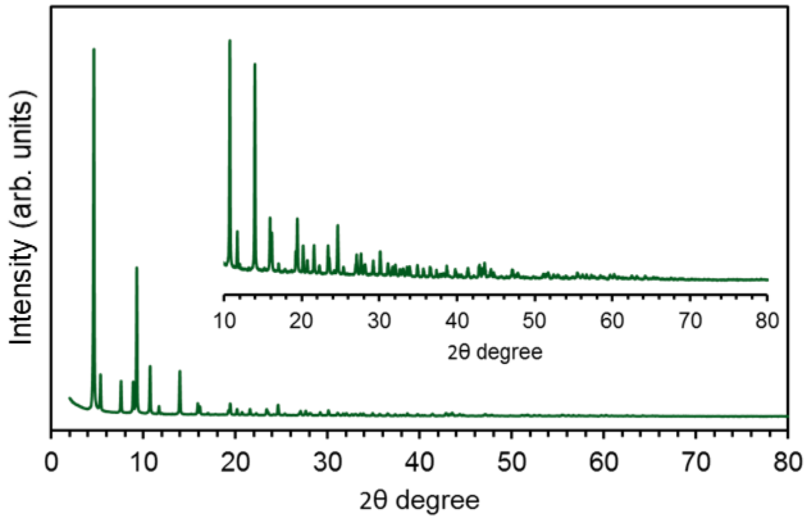

Supplementary Figure 32 | Powder X-ray diffraction pattern of Zr-DPT:DPA-8% collected from 2 to 80 2θ degrees. Inset: enlargement between 10 and 80 2θ degree.

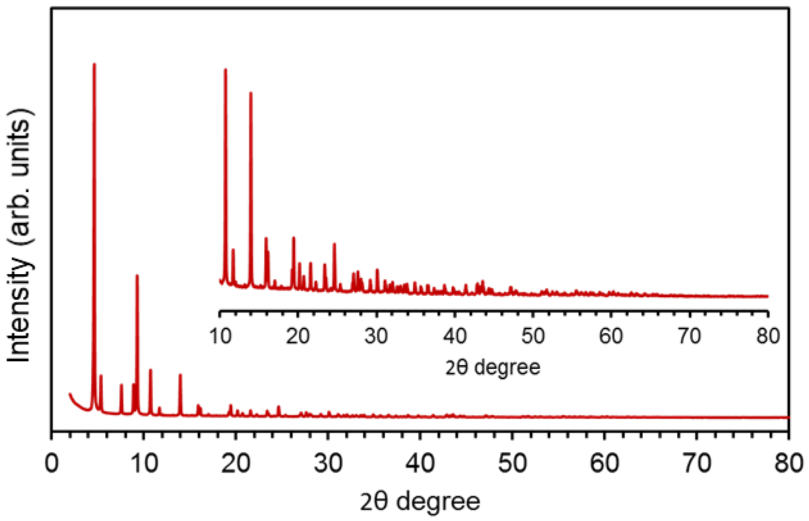

Supplementary Figure 33 |Powder X-ray diffraction pattern of Zr-DPT:DPA-1% collcted from 2 to 80 2θ degrees. Inset: enlargement between 10 and 80 2θ degree.

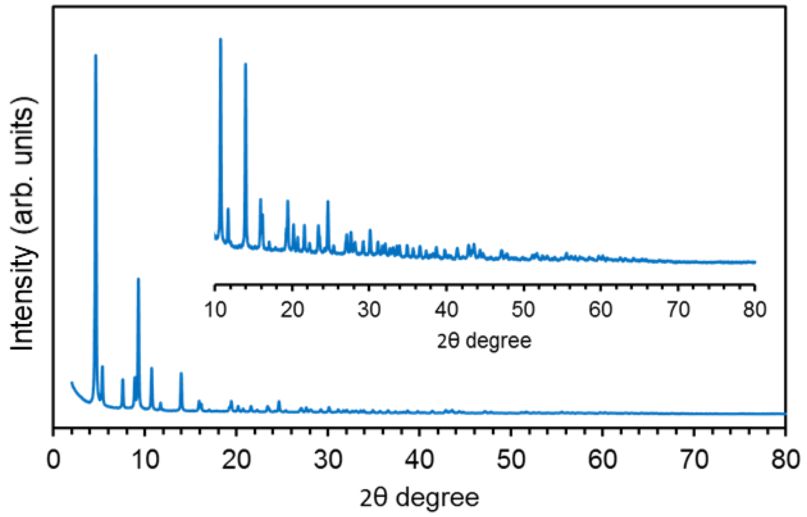

Supplementary Figure 34 |. Powder X-ray diffraction pattern of Zr-DPT:DPA-0.5% collected from 2 to 80 2θ degrees. Inset: enlargement between 10 and 80 2θ degree.

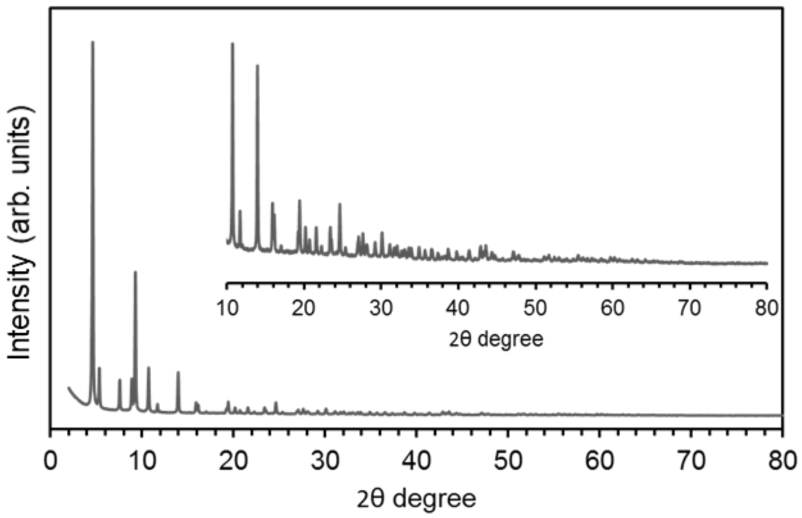

Supplementary Figure 35 |. Powder X-ray diffraction pattern of Zr-DPT:DPA-0.1% collcted from 2 to 80 2θ degrees. Inset: enlargement between 10 and 80 2θ degree.

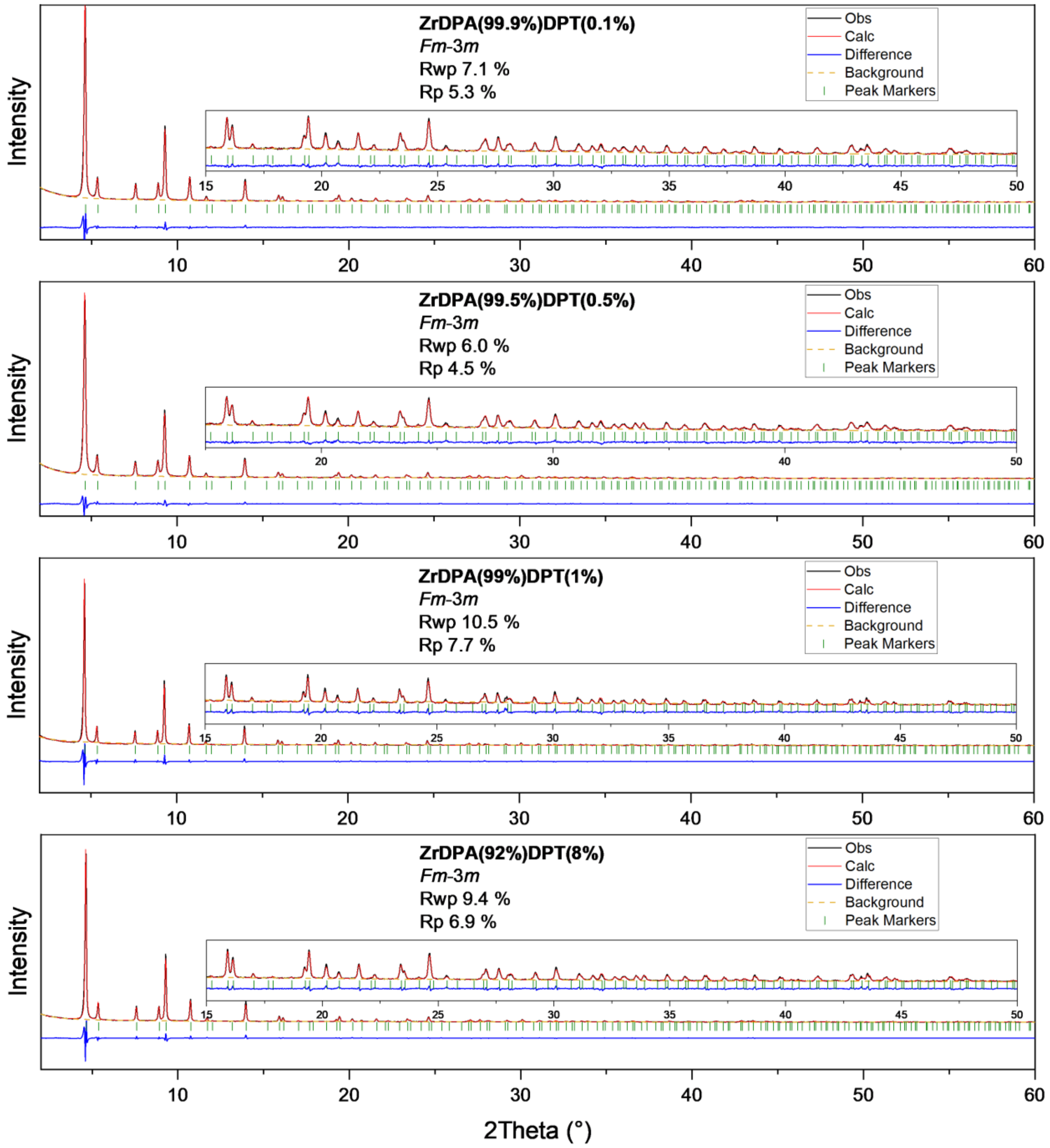

Supplementary Figure 36 | Rietveld fits of X-ray data for Zr-DPT:DPA-0.1%, Zr-DPT:DPA-0.5%, Zr-DPT:DPA-1% and Zr-DPT:DPA-8%, shown from top to bottom, refined using the TOPAS-Academic-64 V6 software package. [2] [3]The HKL indices are indicated as green markers and the difference plot is shown below. The 3black and red lines represent the observed and calculated traces respectively while the dotted yellow line represents the background plot.

4.9 Nitrogen adsorption isotherms at 77 K and sorption properties

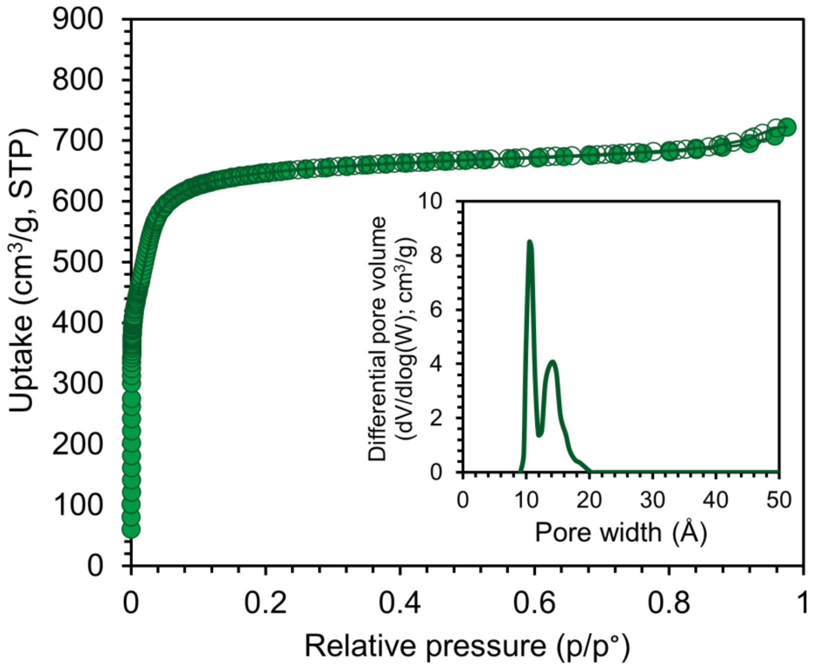

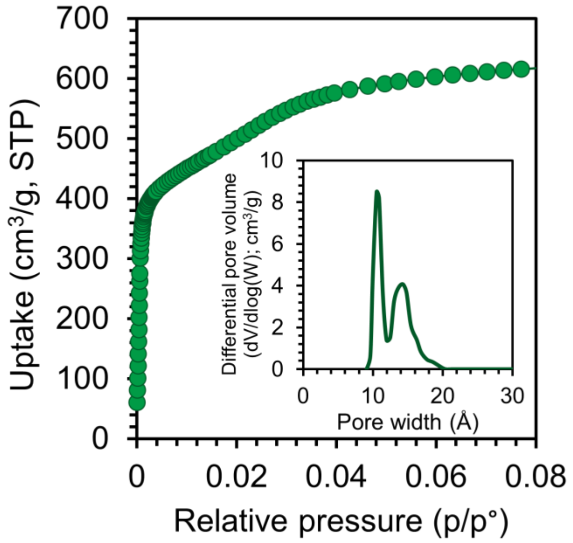

Supplementary Figure 37 | Nitrogen adsorption isotherm of Zr-DPT:DPA-8% collected at 77 K. Inset: Pore size distribution calculated according to NLDFT theory (N_2 on carbon slit pore method). The pore size distribution showed 2 distinct cavities of diameter 10.8 Å and 15 Å. Below: enlargement between 0 and 0.08 p/p° showing the change in adsorption isotherm slope related to the bimodal pore size distribution in the micropore region.

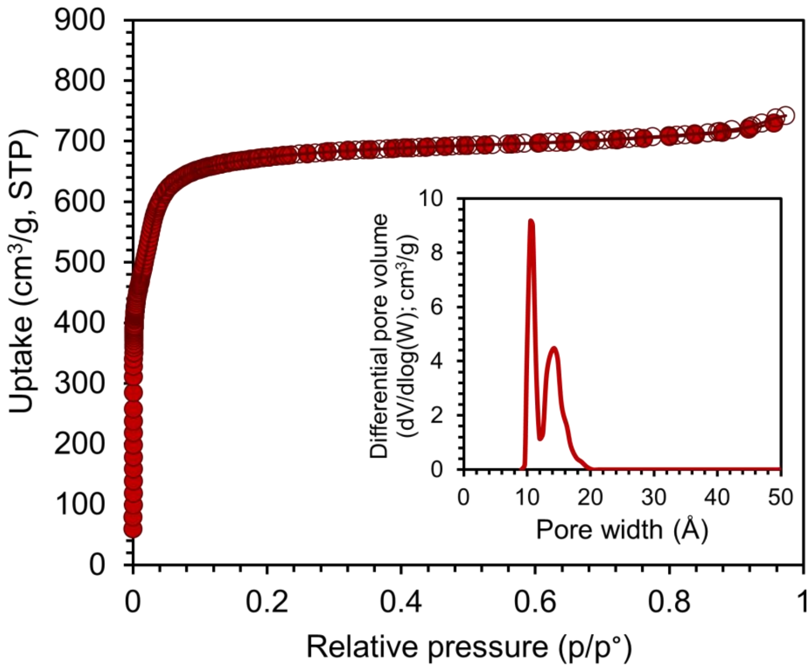

Supplementary Figure 38 | Nitrogen adsorption isotherm of Zr-DPT:DPA-1% collected at 77 K. Inset: Pore size distribution calculated according to NLDFT theory (N_2 on carbon slit pore method).

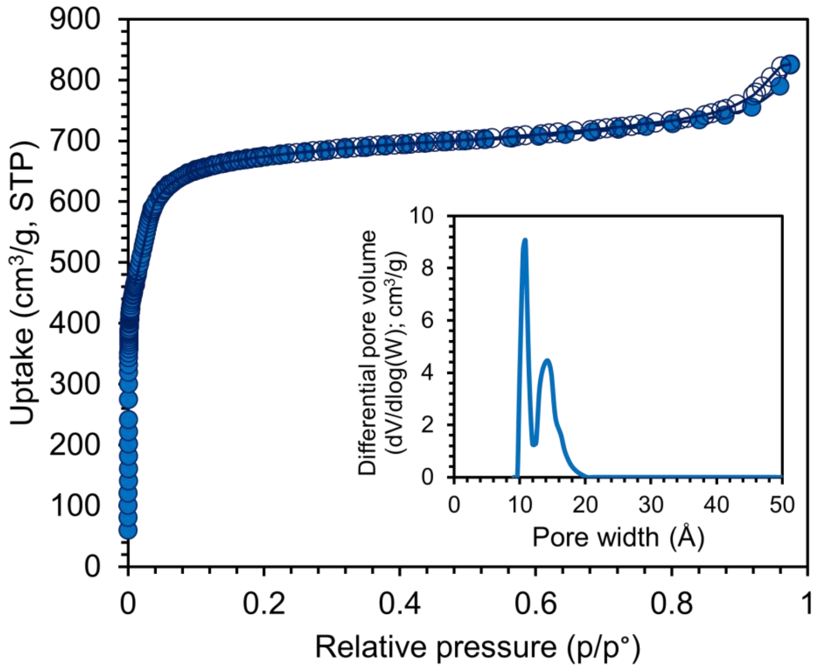

Supplementary Figure 39 | Nitrogen adsorption isotherm of Zr-DPT:DPA-0.5% collected at 77 K. Inset: Pore size distribution calculated according to NLDFT theory (N_2 on carbon slit pore method).

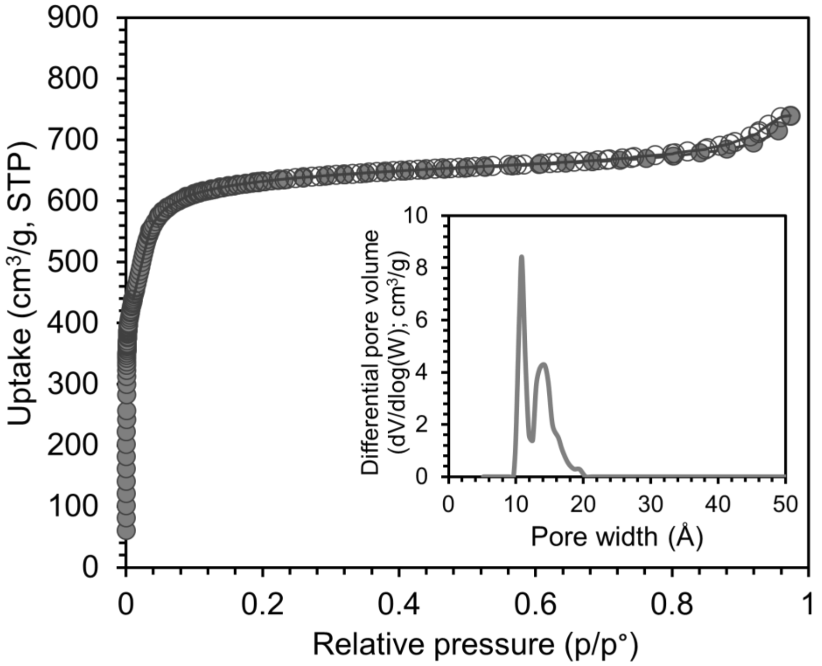

Supplementary Figure 40 | Nitrogen adsorption isotherm of Zr-DPT:DPA-0.1% collected at 77 K. Inset: Pore size distribution calculated according to NLDFT theory (N_2 on carbon slit pore method).

Supplementary Table 6 | Adsorptive properties of Zr-DPT:DPA-x% and Zr-DPT samples.

**Sample**	**Langmuir surface area* (m^2/g)**	**BET surface area* (m^2/g)**	**Micropore volume*' (cm^3/g)**	**Total pore volume (cm^3/g)**	**Micro- to total pore ratio (%)**
Zr-DPT:DPA-0.1%	2840	2500	0.89	0.96	93
Zr-DPT:DPA-0.5%	3035	2670	0.94	1.05	90
Zr-DPT:DPA-1%	3045	2680	0.95	1.00	95
Zr-DPT:DPA-8%	2910	2570	0.91	0.95	96
Zr-DPT	2240	1950	070	0.71	98.5

*Calculated between 0.015 and 0.1 p/p°: *' Calculated up to 2 nm, according to IUPAC recommendation.

5. EXCITON DIFFUSION/ENERGY TRANSFER MODELING AND SUPPLEMENTARY DATA

<u>*Time resolved data analysis.*</u>

The time resolved photoluminescence (PL) spectra and scintillation data discussed in the main text show in general a complex behavior. The signal decay has been reproduced with an analytical multi-exponential function

$$I_{PL}(t) \propto \sum_i A_i e^{-\left(t/\tau_i\right)} . \qquad \text{Eq. 1}$$

The parameters used for the fitting procedure are reported in Table 2. The characteristic emission lifetime τ_{avg} has been calculated as the weighted average of the characteristic decay time for each *i*-exponential function by

$$\tau_{avg} = \frac{\sum_i A_i \tau_i}{\sum_i A_i} . \qquad \text{Eq. 2}$$

Supplementary Table 7 | Fit parameters employed to analyze the time resolved photoluminescence recorded on co-assembled MOFs dispersions in THF and scintillation experiments on co-assembled MOFs nanocomposites.

**@430 nm**	**τ_1 (ns)**	**A_1**	**τ_2 (ns)**	**A_2**	**τ_{avg} (ns)**
**MOF**					
**0%**	4.40	1.00	-	-	4.40
**0.1%**	3.51	1.00	-	-	3.51<br>$\phi_{ET} = 0.48$
**0.5%**	0.25	0.50	1.30	0.50	0.78<br>$\phi_{ET} = 0.88$
**1%**	0.22	0.48	0.52	0.52	0.37<br>$\phi_{ET} = 0.94$
**8%**	0.12	0.67	0.56	0.33	0.26<br>$\phi_{ET} = 0.96$
**@540 nm**	**τ_1 (ns)**	**A_1**	**τ_2 (ns)**	**A_2**	**τ_{avg} (ns)**
**Zr-DPT/DPA-1%**					
**dilution 1:1**	10.97	1.00	-	-	10.97
dilution 1:5	11.04	1.00	-	-	11.04
dilution 1:10	11.01	1.00	-	-	11.01
dilution 1:20	10.95	1.00	-	-	10.95
**Zr-DPT**	3.10	0.49	10.50	0.51	7.71
**DPT**	11.52	1.00	-	-	11.52
**Zr-DPT/DPA-8%**	10.80	1.00	-	-	-
**Zr-DPT/DPA-8% composite small**	10.92	1.00	-	-	-
**Zr-DPT/DPA-8% composite big**	10.89	1.00	-	-	-
**Zr-DPT/DPA-8% composite big scintillation**	0.36	0.22	13.4	0.78	10.4

Singlet exciton diffusion in MOF nanocrystals and energy transfer.

The random diffusion of DPA singlet exciton within the MOF nanocrystals has been modelled as hopping-mediated process occurring between isoenergetic centers, i.e. the framed DPA ligands. Dealing with a 3D isotropic structure, the singlet diffusivity can be calculated as

$$D = mk_{hop}^{DPA-DPA}a^2, \qquad \text{Eq. 3}$$

where a = 1.2 nm is the center-to-center DPAs distance, $k_{hop}^{DPA-DPA}$ is the rate of the hopping process between a ligands pair and m is the number of equivalent nearest neighbor sites surrounding the exciton. Given that dipole permitted electronic transition involved and considering the short intermolecular distances involved, we assume that the hopping is ruled by Förster ET between DPA molecules, by neglecting exchange interaction–driven mechanism. Therefore, with this assumption k_{hop} equals the Forster ET rate $k_{ET}^{DPA-DPA}$between two DPA, which can been calculated referring to the Förster radius $R_{fs}^{DPA-DPA}$. This latter is defined as the distance at which the ET efficiency is 0.5, and it can be derived from experimental data on the molar extinction coefficient $\varepsilon(\lambda)$ and photoluminescence $PL(\lambda)$ of DPA (Supplementary Fig. 7). [7] Specifically, the radius value is given by

$$R_{fs}^{DPA-DPA} = 0.211[J(\lambda)\theta^2 n^{-4}\mathrm{QY_{DPA}}]^{1/6}, \qquad \text{Eq. 4}$$

where θ^2 is the orientation factor between the transition momentum of the involved electronic transitions on the donor and acceptor systems, n = 1.7 is the refraction index and $\mathrm{QY_{DPA}}$= 0.96 is the DPA photoluminescence quantum yield. The overlap integral $J(\lambda)$ is calculated form experimental data as

$$J(\lambda) = \frac{\int PL(\lambda)\varepsilon(\lambda)\lambda^4 d\lambda}{\int PL(\lambda)d\lambda} \ , \qquad \text{Eq. 5}$$

thus obtaining $R_{fs}^{DPA-DPA}$ = 2.28 nm (with λ expressed in nanometres). The total hopping rate calculated with m = 6 and a singlet lifetime τ_{DPA}= 6.7 ns is then

$$k_{hop}^{DPA-DPA} = 6k_{ET}^{DPA-DPA} = 6\frac{1}{\tau_{DPA}}\left(\frac{a}{R_{fs}^{DPA-DPA}}\right)^{-6} = 49 \text{ GHz}. \qquad \text{Eq. S6}$$

This result allows to calculate the total singlets excitons diffusivity D = 6.7×10^{-4} cm^2 s^{-1}. We employed the τ_{DPA}= 6.7 ns value because, we know that the DPA ligand is not affected by incorporation in the MOF architecture. The experiment performed on large MOF crystals indicates indeed the absence of intermolecular interactions or metal-related interactions that can affect it. [1,8] Thus, for our modeling it is correct to consider the DPA lifetime as individual ligand as reference parameter to evaluate both the hopping rate, that rules the exciton diffusion, and the ET rate to the embedded DPT molecules (vide infra). The ideal 3D diffusion length of singlets in isotropic systems such as the MOF, is calculated as large as $L = \sqrt{6D\tau_{DPA}}$ = 127 nm, which corresponds to a potential diffusion sphere of volume $V_{diff} = \frac{4\pi}{3}\left(\frac{L}{2}\right)^3$ that is explored by the singlet during its random walk before recombination. This means that, before recombination, the DPA singlet can ideally explore a sphere of volume V_{diff} = 1.1×10^6 nm^3. This value is comparable to the volume of the octahedral nanocrystals with average diameter 185 nm considered here, V_{MOF} ~ 10^6 nm^3. In other words, the DPA singlets can explore approximately the nanocrystal volume during their lifetime, thus encountering any of the incorporated DPT molecules with the same probability and therefore reaching the *rapid diffusion limit* regime. [9] Also by using the surface quenched lifetime of Zr-DPA MOF the diffusion volume remains ~ 0.8×10^6 nm^3, thus making reasonably valid the rapid diffusion assumption. Notably, the real value of the hopping rate can be only larger than the one considered here since also exchange-mediated transfer and the contribution of the second/third nearest neighbors must be taken in to account, considering the close-packed arrangement of DPA ligands in the framework. All these effects would of course increase the diffusion speed of the exciton, thus further supporting the achievement of the rapid diffusion condition for the process.

In the rapid diffusion limit the ET rate k_{ET} to dopants is calculated as

$$k_{ET} = \frac{4\pi C_{DPT} R_{fs}^6}{3\tau_{DPA} a^3} \qquad \text{Eq. 7,}$$

Where R_{fs} = 2.8 nm is the Förster radius that characterizes this donor/acceptor pair, calculated from the data in Fig. S42 and $C_{DPT}$is the acceptor concentration. The theoretical ET efficiency is estimated as a function of the DPT amount incorporated in the crystal by

$$\phi_{ET} = \frac{k_{ET}}{k_{ET}+k_{DPA}}, \qquad \text{Eq. 8,}$$

where $k_{DPA} = (\tau_{DPA})^{-1}$ = 0.15 GHz is the spontaneous decay rate of DPA luminescence (Supplementary Fig.42). The experimental ET yield ϕ_{ET} vs. C_{DPT} has been calculated from the time resolved data showed in Fig. 3 (Supplementary Table 7), by using the relationship

$$\phi_{ET} = 1 - \frac{\tau_{avg}}{\tau_{DPA}}. \qquad \text{Eq. 9,}$$

The efficiency values have been fitted with Eq. 9 combined with Eq. 8 and 7, thus resulting an experimental interaction radius between DPA and DPT molecules of R_{fs}^{*}= 3.0 nm.

Photoluminescence Quantum Yield Measurements.

The fluorescence quantum yield ϕ_{pl} of MOF nanocrystals dispersions has been determined by relative method for yield measurements. The ϕ_{pl} was determined relative to 10^{-5} solution of DPA in cycloexane (ϕ_{std}= 0.96),[10] which has used as secondary standard according to the following equation

$$\phi_{\mathrm{unk}} = \phi_{\mathrm{std}} \left(\frac{\mathrm{Ast_{std}}}{\mathrm{Ast_{unk}}}\right)\left(\frac{\mathrm{I_{unk}}}{\mathrm{I_{std}}}\right)\left(\frac{\mathrm{P_{std}}}{\mathrm{P_{unk}}}\right)\left(\frac{\eta_{\mathrm{unk}}}{\eta_{\mathrm{std}}}\right)^2, \qquad \text{Eq. 10}$$

where ϕ_{unk}, $\mathrm{Ast_{unk}}$, $\mathrm{I_{unk}}$, $\mathrm{P_{unk}}$ and η_{unk} represent the quantum yield, absorptance at the excitation wavelength of 370 nm, integrated photoluminescence spectral profile, excitation power density, and refractive index of the investigated material. The corresponding terms for the subscript "std" are for the reference quantum counter DPA at the identical excitation wavelength. To avoid any detrimental effect of the scattering of the excitation light that could avoid a proper determination of the samples absorptance, the absorption spectrum of MOF dispersion has been measured using an integrating sphere. The ϕ_{pl} of dicarboxyl-DPT in diluted solution has been measured using the Rhodamine 6G in ethanol (10^{-5} M, ϕ_{std}=0.95) as secondary standard. [11]

Estimation of the doping reaction yield and doped MOF 8% photoluminescence quantum yield.

According to the data in Fig. 3c and Supplementary Table 2, the time resolved analysis of the residual photoluminescence from DPA ligands in the MOF-8% suggests the presence of two subpopulation of nanocrystals. The emission intensity decay shows two components, with relative weight of 67% for the fast one and 33% for the slow one. The fast component mirrors the quick diffusion-mediated ET in effectively doped nanocrystals, while we ascribe the slow component to a subpopulation of less doped MOFs in the ensemble. The energy transfer rate in the fully active population is as large as $k_{fast} = (\tau_{fast})^{-1}$ = 8 GHz thus resulting in a complete ET ($k_{fast} \gg k_{DPA}$). Conversely, the partially active population is the main responsible of the residual blue emission observed in the steady-state PL spectrum reported in Fig.4A, that result a partial quenching of the DPA emission ($k_{slow} = (\tau_{slow})^{-1} =$ 1.8 GHz).

This latter population represent therefore a competitive recombination pathway for the molecular excitons generated in the nanocrystal ensemble, which lowers the global system photoluminescence quantum yield in the DPT emission spectral range. We can assume that the doping reaction has a yield of η = 67%, which corresponds to the fraction of fully active emitters with large Stokes shift. This allows us to estimate the expected photoluminescence QY in first approximation. Considering that the DPT related emission in doped crystals shows the same recombination dynamics that the single molecule in diluted solution (Fig. 41), we can assume that that no competitive mechanism affects heavily the emission yield of framed DPTs, which efficiency $QY_{DPT,framed}$ = 0.75±0.08 can be derived comparing the QY_{DPT} in solution (0.8±0.08) and the emission lifetimes in solution and in the MOFs (Fig. S41b,c). Therefore, the expected yield of the nanocrystals ensemble QY_{ex} can be calculated as

$$QY_{expected} = \eta * QY_{DPT,framed} * (\phi_{ET} = 1) + (1-\eta) * QY_{DPT,framed} * (\phi_{ET} = 0.9) = 0.72 \pm 0.08$$

Eq. 11

which, considering the typical uncertainty of the QY measurements, is in good agreement with the measured value of QY^{ens} = 0.56 ± 0.06. This demonstrate that the photoluminescence yield of doped nanocrystals does not have intrinsic limitation due to competitive fast recombination mechanisms but is actually set by the doping reaction yield.

The real photoluminescence yield of fully active nanocrystals QY can be therefore estimated from experimental parameters using

$$QY_{ens} = \eta * QY * (\phi_{ET} = 1) + (1-\eta) * QY * (\phi_{ET} = 0.9) = 0.59 \pm 0.07$$ Eq. 12

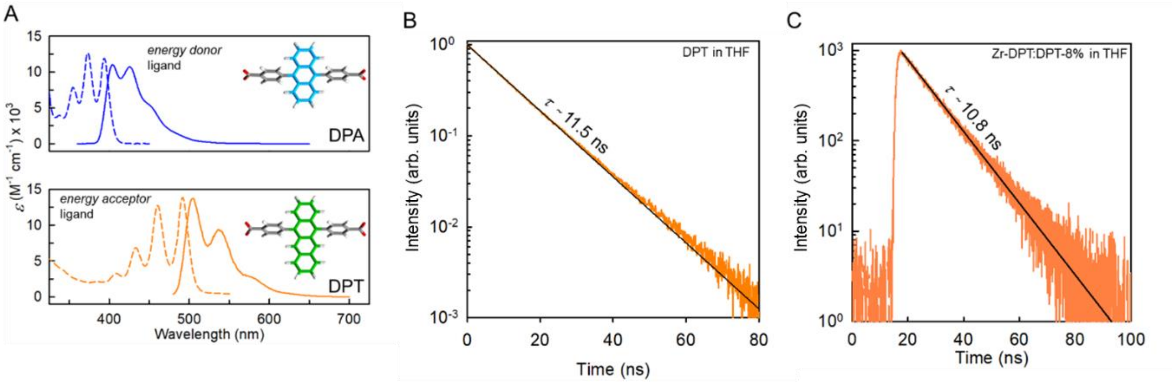

Supplementary Figure 41 | A. Molar extinction coefficient and PL spectrum in the UV-Vis spectral range of DPA and DPT in THF under excitation at 370 nm and 405 nm, respectively. **B.** Time resolved photoluminescence spectrum at of DPT ligands in THF diluted solution (10^{-7} M) under pulsed excitation at 405 nm. The solid line is the fit of data with a single exponential decay function with characteristic lifetime τ of 11.5 ns. **C.** Time resolved photoluminescence spectrum at ofZr-DPT:DPA-8% MOF nanocrystals in THF diluted dispersion under pulsed excitation at 405 nm. The solid line is the fit of data with a single exponential decay function with characteristic lifetime τ of 10.8 ns.

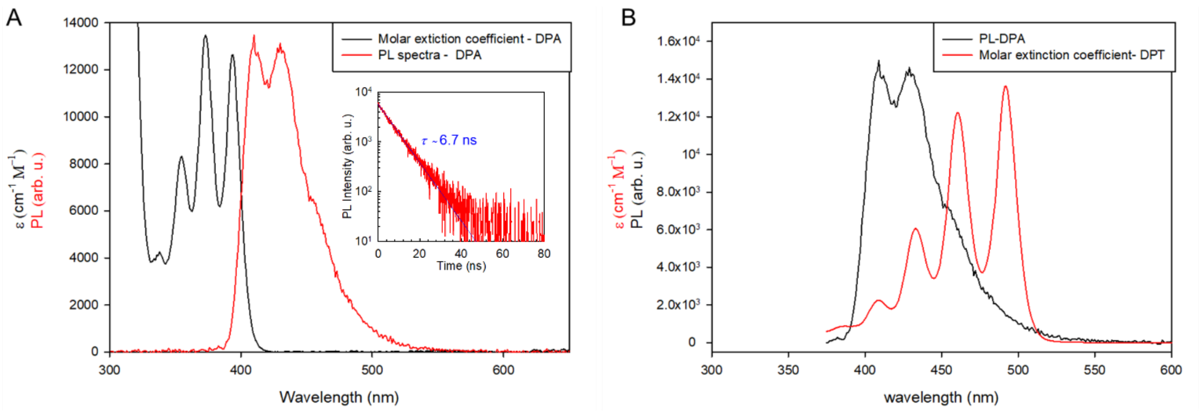

Supplementary Figure 42 | A. Molar extinction coefficient and PL spectrum in the UV-Vis spectral range of DPA in THF under excitation at 370 nm. The inset shows the PL intensity decay with time under pulsed excitation at 350 nm. The solid line is the fit with a single exponential decay function with a characteristic decay time of $\tau_{DPA} = 6.7$ ns, which corresponds to a spontaneous decay rate of $k_{DPA} = (\tau_{DPA})^{-1}$of 0.15 GHz. B. Energetic resonance between the molar extinction coefficient of dicarboxylic DPT and DPA PL spectrum in THF.

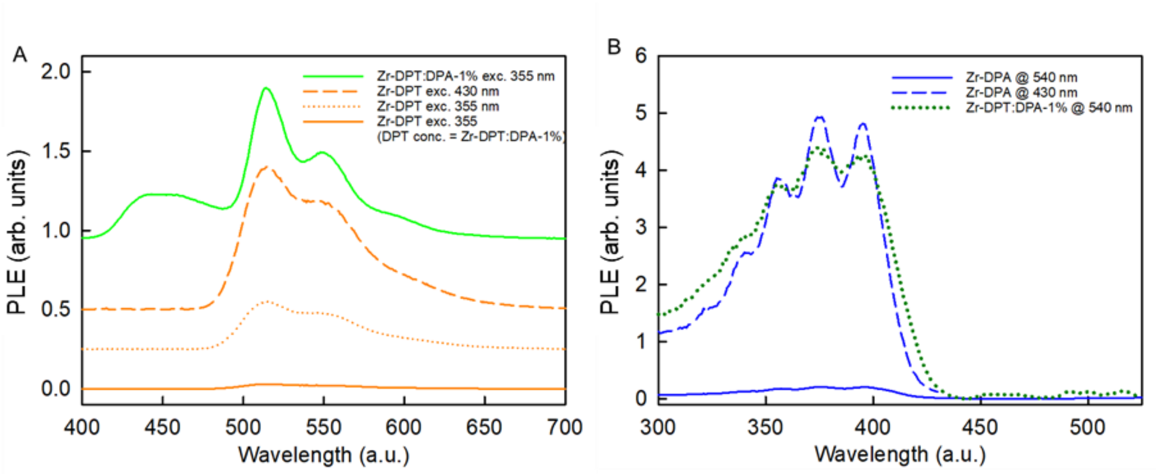

Supplementary Figure 43 | A, Photoluminescence (PL) spectra of the *hetero*-ligand Zr-DPT:DPA-1% nanocrystals (0.1 mg mL^{-1}) in tetrahydrofuran (THF) compared with *homo*-ligand Zr-DPT MOF dispersion (0.1 mg mL^{-1}). The excitation wavelength is reported in the legend. The solid line is PL spectrum of the Zr-DPT dispersion diluted ten times to have the same amount of DPT ligands as in the Zr-DPT:DPA-1% sample. **B,** Excitation PL (PLE) spectra of Zr-DPA and the Zr-DPT:DPA-1% dispersion recorded under the same experimental conditions. The detection wavelength is reported in the legend.

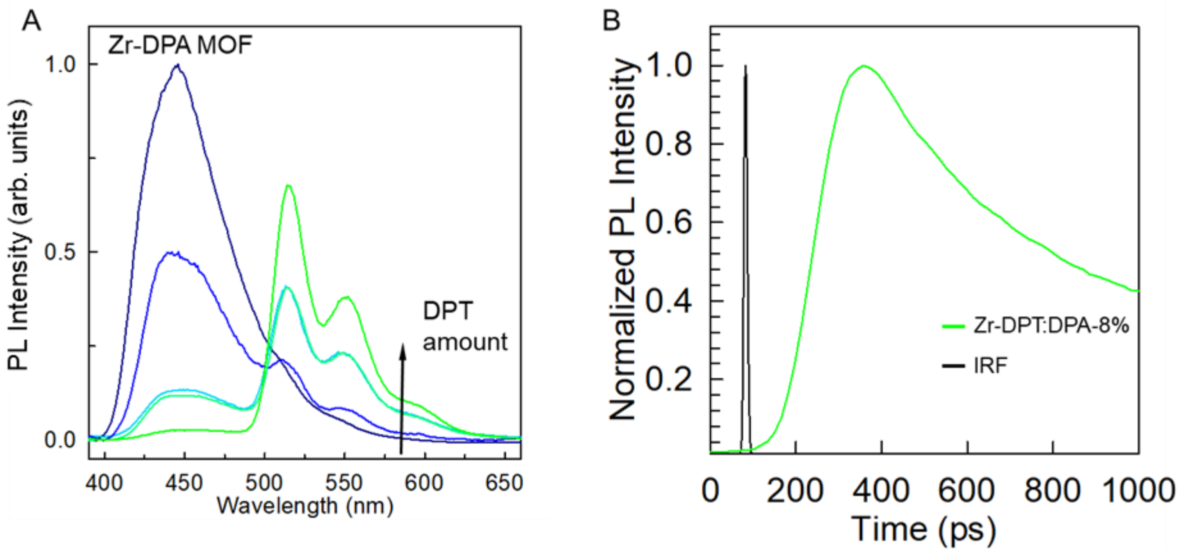

Supplementary Figure 44 | A Photoluminescence (PL) spectra of the *hetero*-ligand Zr-DPT:DPA-x% nanocrystals series investigated (0.1 mg mL^{-1}) in tetrahydrofuran (THF) compared with *homo*-ligand MOFs based on DPA (Zr-DPA, 0.1 mg mL^{-1}). The excitation wavelength is 355 nm. The spectra are recorded under the same experimental conditions. **B,** Time-resolved PL spectra recorded at 540 of the Zr-DPT:DPA 8% MOF dispersion under ultrafast pulsed excitation at 370 nm.

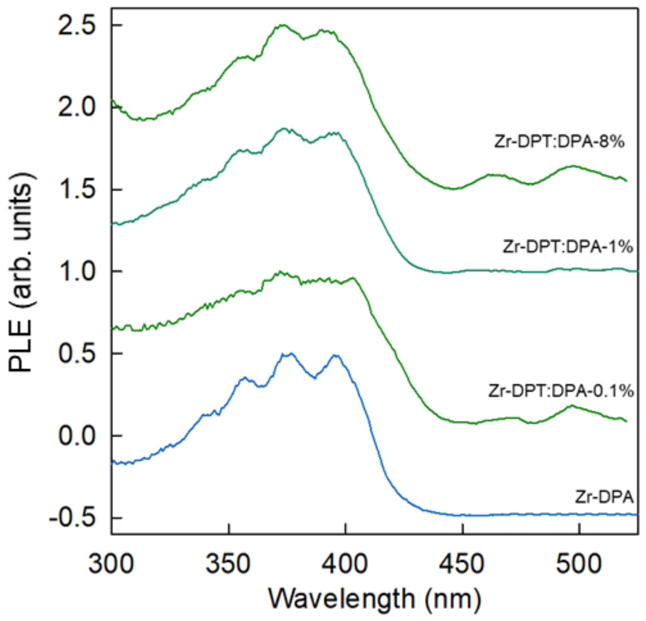

Supplementary Figure 45 | Excitation PL (PLE) spectra of *hetero*-ligand Zr-DPT:DPA-x% and *homo*-ligand Zr-DPA nanocrystals monitored at 540 nm.

6. Zr-DPT:DPA-8% MOF:PDMS nanocomposite

6.1 Synthetic procedure for Zr-DPT:DPA-8%:PDMS nanocomposite

General procedure for Zr-MOF:PDMS composite preparation (0.5% wt). PDMS prepolymer RTV 615 base (3.0 g) was poured in a 15 mL polypropylene tube and a 0.5 mL of a dispersion of Zr-DPT:DPA-8% MOFs (16.5 mg) in dry tetrahydrofuran was added. The mixture was stirred under vigorous stirring and successively RTV 615 curing agent (0.3 g) was added. After a careful mixing, the homogenous mixture was degassed under vacuum for 2 hours and then poured onto a proper glass mold. A second degassing procedure was performed, and the sample was heated under vacuum in an oven at 60 °C for 16 hours. The specimens were slowly cooled to room temperature and removed from the mold. ^{1}H NMR ($CDCl_3$), prepolymer base: δ (ppm) = 0.07 (s, dimethylsiloxane), 5.75-6.15 (m, vinyl). ^{1}H NMR ($CDCl_3$), prepolymer curing agent: δ (ppm) = 0.07 (s, dimethylsiloxane), 0.23 (m, terminal (C**H**$_3$)$_3$SiO-) 5.75-6.15 (m, methylhydrosiloxane).

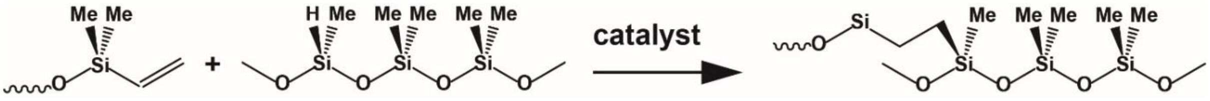

Chemical structures for the formation of cross-linked PDMS.

6.2 Digital image of samples under ambient and UV light

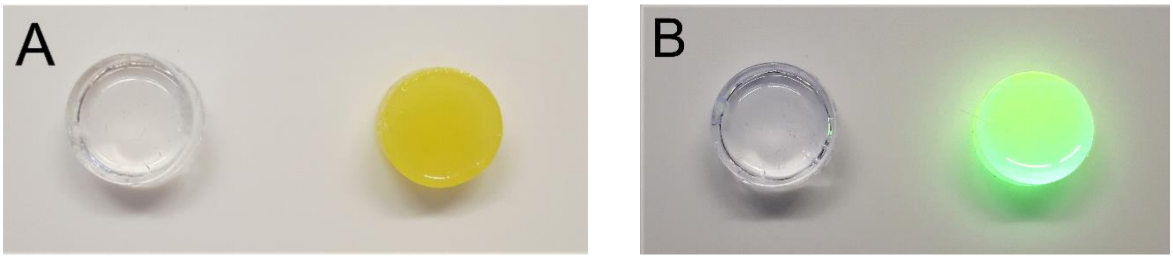

Supplementary Figure 46 | Digital image of blank PDMS (left) and Zr-DPT:DPA-8%:PDMS composite (right) under sunlight (A) and UV irradiation (wavelength: 365 nm, B).

6.3 Differential scanning calorimetry (DSC) of Zr-MOF:PDMS nanocomposite

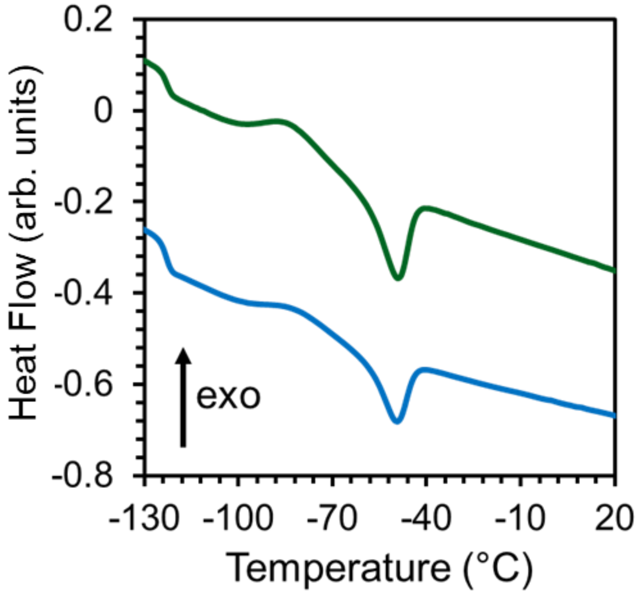

Supplementary Figure 47 | DSC thermogram of of Zr-DPT:DPA-8%:PDMS nanocomposite (top, green) and pure PDMS (Bottom, light blue). measured between -130°C and 20°C under nitrogen atmosphere.

6.4 Infrared spectroscopy of Zr-MOF:PDMS nanocomposite

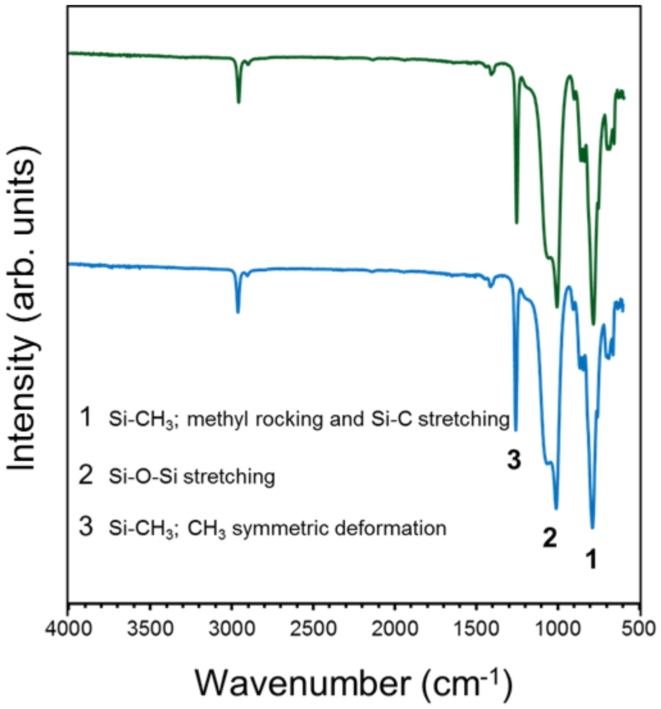

Supplementary Figure 48 | Infrared spectra of Zr-DPT:DPA-8%:PDMS nanocomposite (top, green) and pure PDMS (Bottom, light blue). The principal vibrational bands are highlighted in the spectrum.[12]

6.5 ^{13}C CP MAS spectrum of the Zr-MOF:PDMS nanocomposite

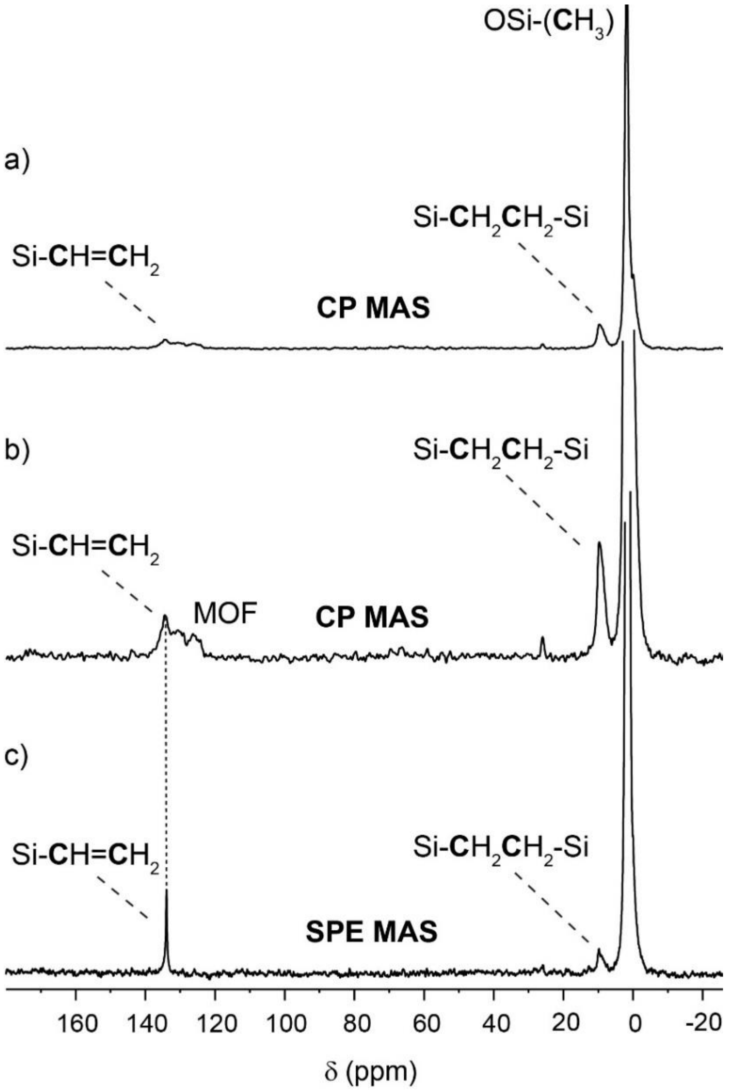

Supplementary Figure S49 | a) ^{13}C CP MAS spectrum of the nanocomposite Zr-DPT:DPA-8%:PDMS with a contact time of 2 ms. b) enhancement of ^{13}C CP MAS spectrum highlighting the cross-linking groups at 9.0 ppm and residual vinyl groups at 134.0 ppm. c) ^{13}C Single pulse excitation (SPE) MAS NMR spectrum with long relaxation delay (30 s). The spectra were collected at 75 MHz at 298 K.

7. NANOCOMPOSITES SCINTILLATION PROPERTIES: supplementary data

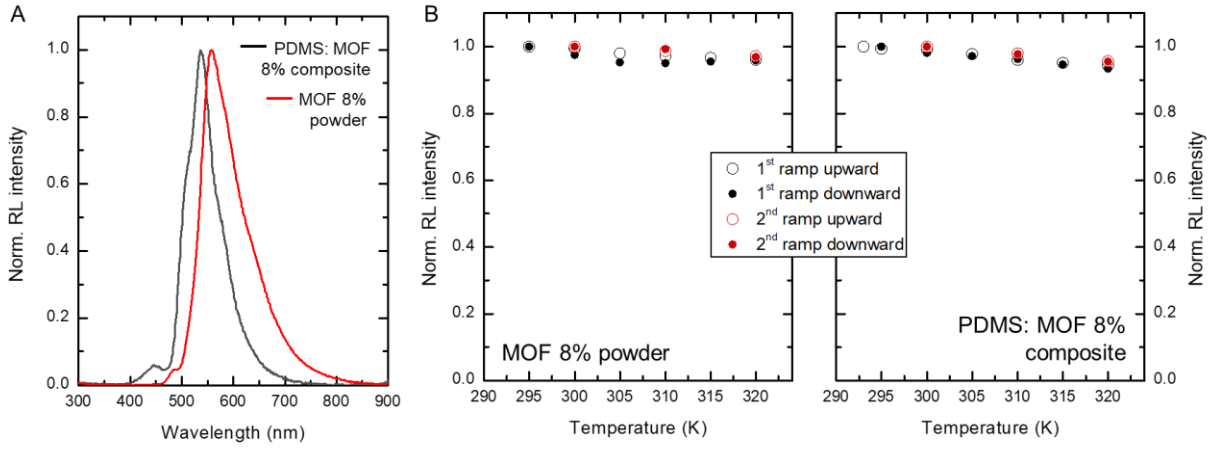

Supplementary Figure 50 | A. Steady-state radioluminescence (RL) spectrum and **B**, integrated RL intensity of bare doped MOF nanocrystals (MOF 8% powder) and nanocomposite (5% wt in PDMS, PDMS:MOF 8%) under X-rays exposure, as function of the temperature. The RL spectrum of the powder spear redshifted due to the partial reabsorption at the extremely high employed amount of nanocrystals (1 mm thick powder sample).

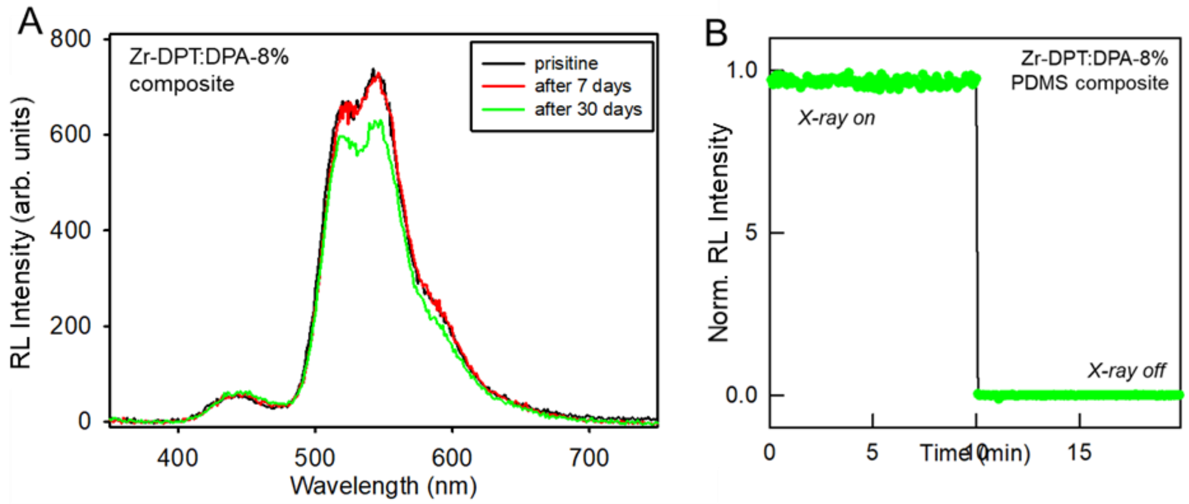

Supplementary Figure 51 | A, Steady-state radioluminescence (RL) spectrum under X-rays exposure of the nanocomposite Zr-DPT:DPA-8% in PDMS as function of the exposure time to the atmospheric moisture. **B**, RL intensity as a function of time (inset).

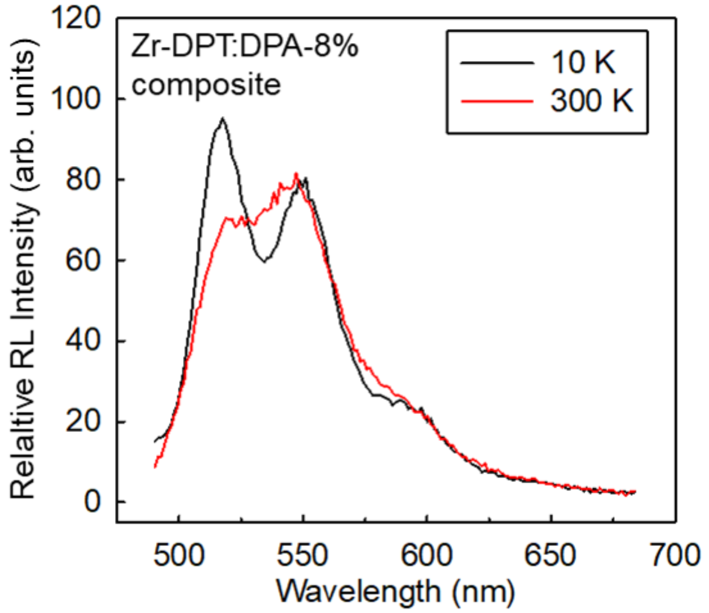

Supplementary Figure 52 | Steady-state radioluminescence (RL) spectrum of a Zr-DPT:DPA-8% composite in PDMS under x-ray exposure at different temperature normalized by the PL lifetime reported in the main text.

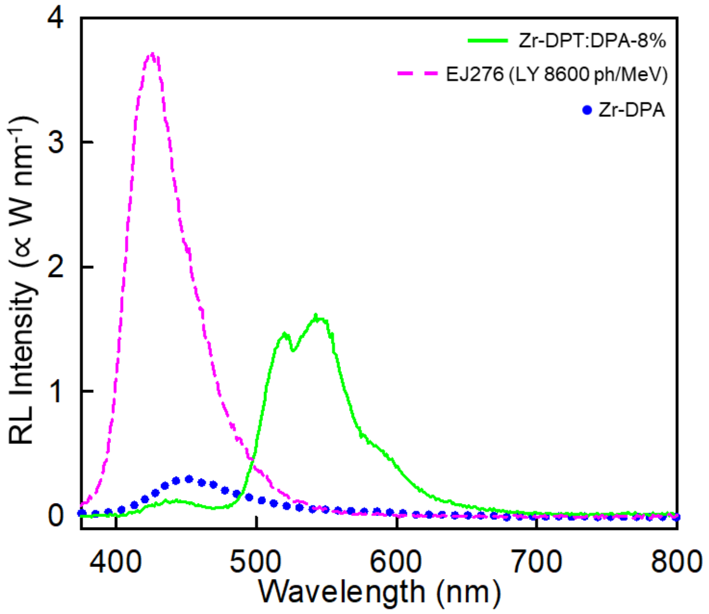

Supplementary Figure 53 | Steady-state radioluminescence (RL) spectrum of a Zr-DPT:DPA-8% composite in PDMS under x-ray exposure compared to the RL of reference Zr-DPA composite in PDMS and of the commercial plastic scintillator EJ276™ (LY 8600 ph MeV^{-1}).

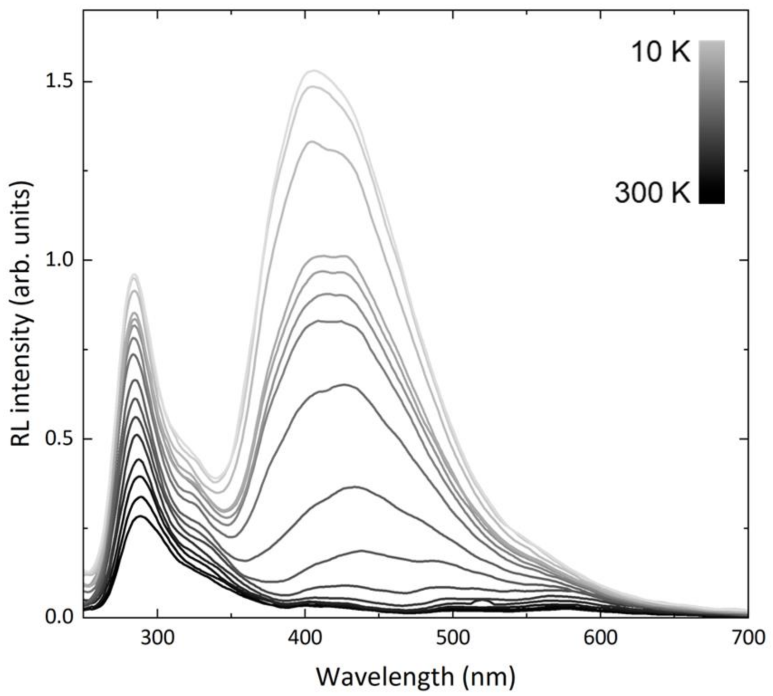

Supplementary Figure S54 | Steady-state radioluminescence (RL) spectrum of a blank PDMS sample under X-rays exposure as function of the temperature.

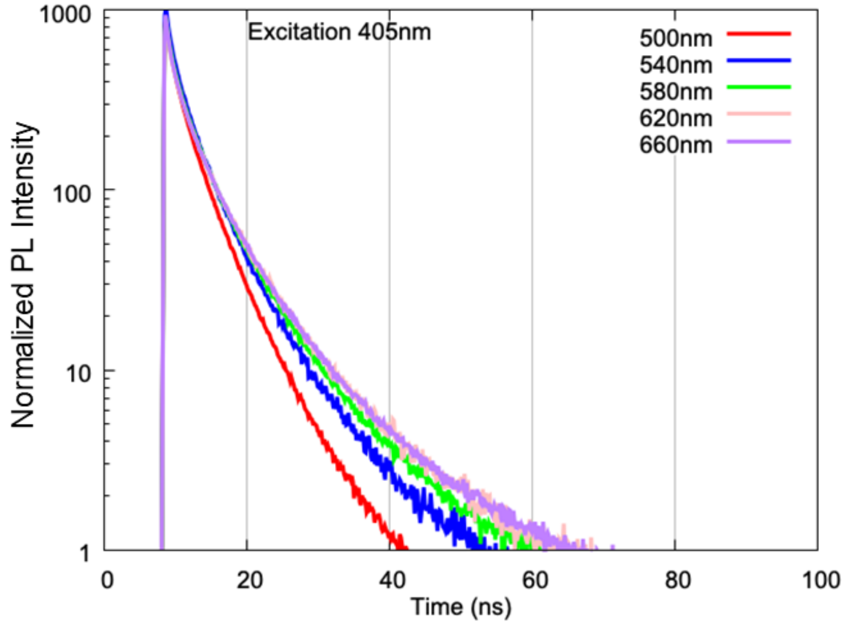

Supplementary Figure 55 | Time resolved photoluminescence spectrum at of nanocomposite sample of thickness 0.5 cm loaded with undoped MOF nanocrystals (MOF-DPA, 0.5% wt.). The decay in time of the emission intensity shows a multiexponential behavior as previously observed (see Ref.4), with an average lifetime of approx. τ =2.8 ns.

8. SUPPLEMENTARY REFERENCES

1 Perego, J. *et al.* Composite fast scintillators based on high-Z fluorescent metal–organic framework nanocrystals. *Nature Photonics* **15**, 393-400, doi:10.1038/s41566-021-00769-z (2021).

2 Coelho, A. Indexing of powder diffraction patterns by iterative use of singular value decomposition. *Journal of Applied Crystallography* **36**, 86-95 (2003).

3 Coelho, A. & Kern, A. Discussion of the indexing algorithms within TOPAS. *CPD Newslett* **32**, 43-45 (2005).

4 Materials Studio: Materials Studio Modeling Environment v7.0.0 (San Diego, 2015).

5 Hill, S. P., Banerjee, T., Dilbeck, T. & Hanson, K. Photon Upconversion and Photocurrent Generation via Self-Assembly at Organic–Inorganic Interfaces. *The Journal of Physical Chemistry Letters* **6**, 4510-4517, doi:10.1021/acs.jpclett.5b02120 (2015).

6 Montalti, M. C. A. P. L. G. M. T. M. J. B. V. *Handbook of photochemistry*. (2020).

7 Lakowicz, J. R. *Principles of fluorescence spectroscopy*. (Springer Science+Business Media, 2010).

8 Monguzzi, A. *et al.* Highly Fluorescent Metal–Organic-Framework Nanocomposites for Photonic Applications. *Nano Letters* **18**, 528-534, doi:10.1021/acs.nanolett.7b04536 (2018).

9 Stryer, L., Thomas, D. D. & Meares, C. F. Diffusion-enhanced fluorescence energy transfer. *Annual review of biophysics and bioengineering* **11**, 203-222 (1982).

10 Morris, J. V., Mahaney, M. A. & Huber, J. R. Fluorescence quantum yield determinations. 9,10-Diphenylanthracene as a reference standard in different solvents. *The Journal of Physical Chemistry* **80**, 969-974, doi:10.1021/j100550a010 (1976).

11 Kubin, R. F. & Fletcher, A. N. Fluorescence quantum yields of some rhodamine dyes. *Journal of Luminescence* **27**, 455-462 (1982).

12 Colthup, N. B. D. L. H. W. S. E. *Introduction to infrared and Raman spectroscopy*. (Academic Press, 1998).
