# Supplementary material for: Highly luminescent scintillating hetero-ligand MOF nanocrystals with engineered Stokes shift for photonic applications
Source: Nat Commun. 2022 Jun 17;13:3504. doi: 10.1038/s41467-022-31163-0 (PMC9205964; doi:10.1038/s41467-022-31163-0)
Supplement: Supplementary file 2 — Description of Additional Supplementary Files [file 41467_2022_31163_MOESM2_ESM.pdf]

Description of Additional Supplementary files

File name: Supplementary Data File 1
Description: Raw data for the Rietveld fits of X-ray data for Zr-DPT:DPA-0.x% structures
